# PEMA: a flexible Pipeline for Environmental DNA Metabarcoding Analysis of the 16S/18S ribosomal RNA, ITS, and COI marker genes

**DOI:** 10.1093/gigascience/giaa022

**Published:** 2020-03-12

**Authors:** Haris Zafeiropoulos, Ha Quoc Viet, Katerina Vasileiadou, Antonis Potirakis, Christos Arvanitidis, Pantelis Topalis, Christina Pavloudi, Evangelos Pafilis

**Affiliations:** Institute of Marine Biology, Biotechnology and Aquaculture (IMBBC), Hellenic Centre for Marine Research (HCMR), Former U.S. Base of Gournes P.O. Box 2214, 71003, Heraklion, Crete, Greece; Department of Biology, University of Crete, Voutes University Campus, Heraklion, Greece; Institute of Marine Biology, Biotechnology and Aquaculture (IMBBC), Hellenic Centre for Marine Research (HCMR), Former U.S. Base of Gournes P.O. Box 2214, 71003, Heraklion, Crete, Greece; Institute of Marine Biology, Biotechnology and Aquaculture (IMBBC), Hellenic Centre for Marine Research (HCMR), Former U.S. Base of Gournes P.O. Box 2214, 71003, Heraklion, Crete, Greece; Charles University, Department of Ecology, Faculty of Science, Viničná 7, CZ-12844, Prague, Czech Republic; Institute of Marine Biology, Biotechnology and Aquaculture (IMBBC), Hellenic Centre for Marine Research (HCMR), Former U.S. Base of Gournes P.O. Box 2214, 71003, Heraklion, Crete, Greece; Institute of Marine Biology, Biotechnology and Aquaculture (IMBBC), Hellenic Centre for Marine Research (HCMR), Former U.S. Base of Gournes P.O. Box 2214, 71003, Heraklion, Crete, Greece; LifeWatch ERIC, Plaza España SN, SECTOR II-III 41013, Seville, Spain; Institute of Molecular Biology and Biotechnology (IMBB), Foundation for Research and Technology (FORTH), Foundation for Research and Technology – Hellas, N. Plastira 100, GR-70013, Heraklion, Crete, Greece; Institute of Marine Biology, Biotechnology and Aquaculture (IMBBC), Hellenic Centre for Marine Research (HCMR), Former U.S. Base of Gournes P.O. Box 2214, 71003, Heraklion, Crete, Greece; Institute of Marine Biology, Biotechnology and Aquaculture (IMBBC), Hellenic Centre for Marine Research (HCMR), Former U.S. Base of Gournes P.O. Box 2214, 71003, Heraklion, Crete, Greece

**Keywords:** pipeline, container, Docker, singularity, high performance computing, HPC, eDNA, metabarcoding

## Abstract

**Background:**

Environmental DNA and metabarcoding allow the identification of a mixture of species and launch a new era in bio- and eco-assessment. Many steps are required to obtain taxonomically assigned matrices from raw data. For most of these, a plethora of tools are available; each tool's execution parameters need to be tailored to reflect each experiment's idiosyncrasy. Adding to this complexity, the computation capacity of high-performance computing systems is frequently required for such analyses. To address the difficulties, bioinformatic pipelines need to combine state-of-the art technologies and algorithms with an easy to get-set-use framework, allowing researchers to tune each study. Software containerization technologies ease the sharing and running of software packages across operating systems; thus, they strongly facilitate pipeline development and usage. Likewise programming languages specialized for big data pipelines incorporate features like roll-back checkpoints and on-demand partial pipeline execution.

**Findings:**

PEMA is a containerized assembly of key metabarcoding analysis tools that requires low effort in setting up, running, and customizing to researchers’ needs. Based on third-party tools, PEMA performs read pre-processing, (molecular) operational taxonomic unit clustering, amplicon sequence variant inference, and taxonomy assignment for 16S and 18S ribosomal RNA, as well as ITS and COI marker gene data. Owing to its simplified parameterization and checkpoint support, PEMA allows users to explore alternative algorithms for specific steps of the pipeline without the need of a complete re-execution. PEMA was evaluated against both mock communities and previously published datasets and achieved results of comparable quality.

**Conclusions:**

A high-performance computing–based approach was used to develop PEMA; however, it can be used in personal computers as well. PEMA's time-efficient performance and good results will allow it to be used for accurate environmental DNA metabarcoding analysis, thus enhancing the applicability of next-generation biodiversity assessment studies.

## Background

Environmental DNA (eDNA) metabarcoding inaugurates a new era in bio- and eco-monitoring [1]. eDNA refers to genetic material obtained directly from environmental samples (soil, sediment, water, etc.) without any obvious signs of biological source material [2]. Metabarcoding is the combination of DNA taxonomy, based on taxa-specific marker genes (e.g., 16S ribosomal RNA [rRNA] for Bacteria and Archaea, cytochrome oxidase subunit 1 [COI] and 18S rRNA for Metazoa, ITS for Fungi), and high-throughput DNA sequencing technologies; thus, simultaneous identification of a mixture of organisms is attainable [3]. eDNA metabarcoding attempts to turn the page on the way biodiversity is perceived and monitored [3]. This combination is considered to be a potential holistic approach that, once standardized, allows for higher detection capacity and at a lower cost compared to conventional methods of biodiversity assessment. However, from the raw read sequence files to an amplicon study's results, the bioinformatics analysis required can be troublesome for many researchers.

Well-established pipelines are available to process metabarcoding data for the case of 16S and 18S rRNA marker genes and bacterial communities (e.g., mothur [4], QIIME 2 [5], LotuS [6]). However, certain limitations accompany each of these and occasionally they can be far from easy-to-use software. Moreover, there is a great need for similarly straightforward and benchmarked approaches for the analysis of other marker genes. With respect to the COI and ITS marker genes, a number of pipelines have been implemented, e.g., Barque [7], ScreenForBio [8], and PIPITS [9]. However, there is still need for a fast, flexible, easy-to-install, and easy-to-use pipeline for both COI and ITS marker genes.

The pipelines mentioned above, although entrenched, are still hindered by a series of hurdles. Among the most prominent are technical difficulties in installation and use, strict limitations in setting parameters for the algorithms invoked, and incompetence in partial re-execution of an analysis.

Moreover, given the computational demands of such analyses, access to high-performance computing (HPC) systems might be mandatory, e.g., to process studies with a large number of samples. This is timely given the ongoing investment of national and international efforts (e.g., [10]) to serve the broad biological community via commonly accessible infrastructures.

PEMA (Pipeline for Environmental DNA Metabarcoding Analysis) is an open source pipeline that bundles state-of-the-art bioinformatic tools for all necessary steps of amplicon analysis and aims to address the aforementioned issues. It is designed for paired-end sequencing studies and is implemented in the BDS [11] programming language. BDS's ad hoc task parallelism and task synchronization supports heavyweight computation, which PEMA inherits. In addition, BDS supports "checkpoint" files that can be used for partial re-execution and crash recovery of the pipeline. PEMA builds on this feature to serve tool and parameter exploratory customization for optimal metabarcoding analysis fine tuning. Switching effortlessly between (molecular) operational taxonomic unit ([M]OTU) clustering and amplicon sequence variant (ASV) inference algorithms is a pertinent example. Finally, via software containerization technologies such as Docker [12] and Singularity [13], with the latter being HPC-centered, PEMA is distributed in an easy to download and install fashion on a range of systems, from regular computers to cloud or HPC environments.

From the biological perspective, monitoring biodiversity at all its different levels is of great importance. Because there is not a single marker gene to detect all taxa, researchers need to use different genes targeting each great taxonomy group separately [14]. To that end, PEMA supports the metabarcoding analysis of both prokaryotic communities, based on the 16S rRNA marker gene, and eukaryotic ones, based on the ITS (for Fungi) and COI and 18S rRNA (for Metazoa) marker genes [14].

As high-throughput sequencing (HTS) data become more and more accurate, ASVs, i.e., marker gene amplified sequence reads that differ in ≥1 nucleotide from each other, become easier to resolve [15]. The use of ASVs instead of OTUs has been suggested [15]; however, the choice of which approach to use should be based on each study's objective(s) [16].

PEMA supports both OTU clustering and ASV inference for all marker genes (see “OTU clustering vs ASV inference” in the “Results and Discussion” section). Two clustering algorithms, VSEARCH [17] and CROP [18], are used for the clustering of reads in (M)OTUs—the former for the case of the 16S/18S rRNA marker genes, the latter for the case of COI and ITS. Swarm v2 [19] allows ASV inference in all cases.

Taxonomic assignment is performed in an alignment-based approach, making use of the CREST LCAClassifier [20] and the Silva database [21] for the case of 16S and 18S rRNA marker genes; the Unite database [22] is used for the ITS gene. In the 16S marker gene case, phylogeny-based assignment is also supported, based on RAxML-ng [23], EPA-ng [24], and Silva [21]. For the COI marker gene, the RDPClassifier [25] and the MIDORI database [26] are used for the taxonomic assignment. In addition, ecological and phylogenetic analysis are facilitated via the “phyloseq” R package [27].

All the pipeline- and third-party module–controlling parameters are defined in a plain "parameter-value pair" text file. Its straightforward format eases the analysis fine tuning, complementary to the aforementioned checkpoint mechanism. A tutorial about PEMA and installation guidance can be found on PEMA's GitHub repository [28].

## Implementation

**Figure 1:**
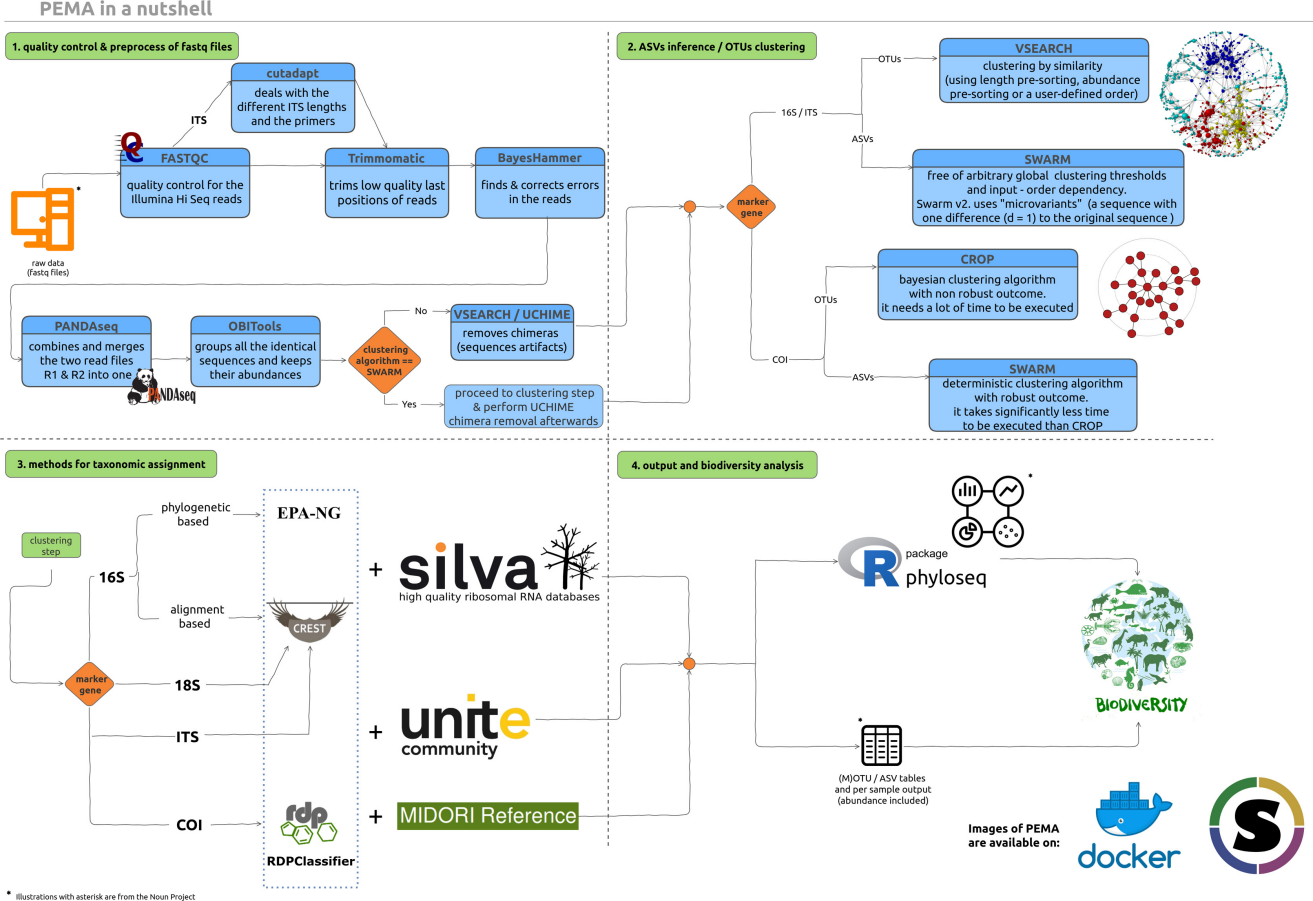
PEMA comprises 4 parts. The first step (top left) is the quality control and pre-processing of the Illumina sequencing reads. This step is common for both 16S rRNA and COI marker genes. The second step (top right) is the clustering of reads to (M)OTUs or their inferring to ASVs. The third step (bottom left) is the taxonomy assignment to the generated (M)OTUs/ASVs. In the fourth step (bottom right), the results of the metabarcoding analysis are provided to the user and visualized. *noun project icons by: ProSymbols (US), IconMark (PH), Nithinan Tatah (TH). clustering figure adapted from DOI: 10.7717/peerj.1420/fig-1.

PEMA's architecture comprises 4 main parts taking place in tandem (Fig. 1). A detailed description of the tools invoked by PEMA and their licenses is included in Additional File 1: Supplementary Methods.

### Part 1: Quality control and pre-processing of raw data

First, FastQC [29] is used to obtain an overall read-quality summary; visual inspection of each sample's quality may recommend removing those insufficient quality, as well as samples with a low number of reads, and rerunning the analysis. To correct errors produced by the sequencer, PEMA incorporates a number of tools. Trimmomatic [30] implements a series of trimming steps, which either remove parts of the sequences corresponding to the adapters or the primers, trim and crop parts of the reads, or even remove a read completely, when it fails to reach the quality-filtering standards set by the user. Cutadapt [31] is used additionally for the case of ITS to address the variability in length of this marker gene (see Additional File 1: Supplementary Methods). BayesHammer [32], an algorithm of the SPAdes assembly toolkit [33], revises incorrectly called bases. PANDAseq [34] assembles the overlapping paired-end reads, and then the “obiuniq” program of OBITools [35] groups all the identical sequences in every sample, keeping track of their abundances. The VSEARCH package [17] is then invoked for chimera removal; however, if the Swarm v2 algorithm is selected, this step will be performed after the ASV inference (see next section).

### Part 2: (M)OTU clustering and ASV inference

Quality-controlled and processed sequences are subsequently clustered into (M)OTUs or treated as input for inferring ASVs. For the case of 16S and 18S rRNA marker genes, VSEARCH [17] is used for OTU clustering, while ASVs can be identified by the Swarm v2 algorithm [19]. VSEARCH is an accurate and fast tool that can handle large datasets; at the same time it is a great alternative for USEARCH [36] because it is distributed under an open source license.

For the ITS and COI marker genes, CROP [18], an unsupervised probabilistic Bayesian clustering algorithm that models the clustering process using birth-death Markov chain Monte Carlo (MCMC), is used. The CROP clustering algorithm is adjusted by a series of parameters that need to be tuned by the user (namely, *b, e*, and *z*). These parameters depend on specific dataset properties such as the length and the number of reads. PEMA automatically adjusts *b, e*, and *z* by collecting such information and applying the CROP recommended parameter-setting rules [18]. ASV inference is conducted by Swarm v2 [19] in this case too.

Because the Swarm v2 algorithm is not affected by chimeras (F. Mahé, personal communication), when Swarm v2 is selected, chimera removal occurs after the clustering (see Additional File 1: Supplementary Methods: Swarm v2). This leads to a computational time gain as chimeras are sought among ASVs, instead of ungrouped reads.

Last, any singletons, i.e., sequences with only 1 read, occurring after the (M)OTU clustering or the ASV inference may be removed according to the user's parameter settings.

### Part 3: Taxonomy assignment

Alignment-based taxonomy assignment is supported for all marker gene analyses. In the case of the 16S/18S rRNA and ITS marker genes, the LCAClassifier algorithm of the CREST set of resources and tools [20] is used together with the Silva [21] and the Unite [22] database, respectively, to assign taxonomy to the OTUs. Two versions of Silva are included in PEMA: 128 (29 September 2016) and 132 (13 December 2017). Because classifiers need first to be trained for each database they use, for future Silva [21] versions new PEMA versions will be available.

For the COI marker gene, PEMA uses the RDPClassifier [25] and the MIDORI reference database [26] to assign taxonomy of the MOTUs. The MIDORI database contains quality-controlled metazoan mitochondrial gene sequences from GenBank [37].

**Figure 2:**
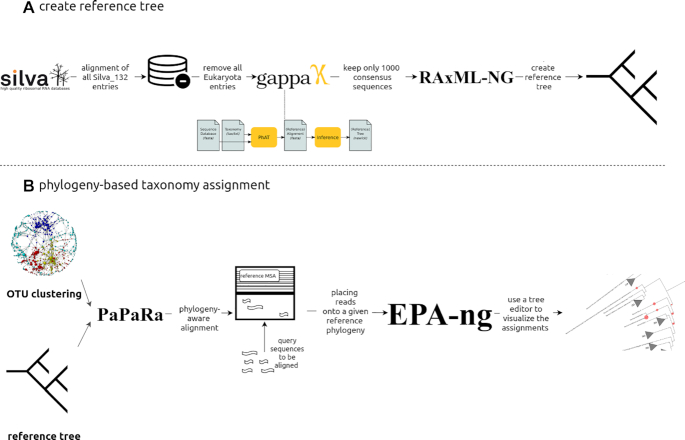
Phylogeny-based taxonomy assignment. A: Building a reference tree for the phylogeny-based taxonomy assignment to 16S rRNA marker gene OTUs: from the latest edition of Silva SSU, all entries referring to Bacteria and Archaea were used and using the “art” algorithm, 10,000 consensus taxa were kept. B: Using PaPaRa and the OTUs that come up from every analysis, an MSA was made and EPA-ng took over the phylogeny-based taxonomy assignment. *noun project icons by: Rockicon and A Beale.

Intended primarily for studies from less explored environments, phylogeny-based assignment is available for 16S rRNA marker gene data. PEMA maps OTUs to a custom reference tree of 1,000 Silva-derived consensus sequences (created using RAxML-ng [23] and gappa [phat algorithm] [38], Fig. 2A). PaPaRa [39] and EPA-ng [24] combine the OTU clustering output and the reference tree to produce a phylogeny-aware alignment and map the 16S rRNA OTUs to the custom reference tree. Beyond the context of PEMA, users may visualize the output with tree viewers such as iTOL [40] (Fig. 2B).

### Part 4: Ecological downstream analysis of the taxonomically assigned (M)OTU/ASV tables

PEMA's major output is either an (M)OTU or an ASV table with the assigned taxonomies and the abundances of each taxon in every sample. For each sample of the analysis, a subfolder containing statistics about the quality of its reads, as well as the taxonomies and their abundances, is also returned.

Via the “phyloseq” R package [27], downstream ecological analysis of the taxonomically assigned OTUs or ASVs is supported. This includes α- and β-diversity analysis, taxonomic composition, statistical comparisons, and calculation of correlations between samples.

When selected, in addition to the phyloseq [27] output, a multiple sequence alignment (MSA) and a phylogenetic tree of the OTU/ASVs retrieved can be returned; for the MSA, the MAFFT [41] aligner is invoked while the latter is built by RAxML-ng [23].

### PEMA container-based installation

An easy way of installing PEMA is via its containers. A Dockerized PEMA version is available [42]. Singularity users can "pull" the PEMA image from [43]. Between the 2 containers, the Singularity-based one is recommended for HPC environments owing to Singularity's improved security and file accessing properties [44]. PEMA can also be found in the bio.tools (id: PEMA) and SciCruch (PEMA, RRID:SCR_017676) databases. For detailed documentation, visit [28].

### PEMA output

All PEMA-related files (i.e., intermediate files, final output, checkpoint files, and per-analysis parameters) are grouped in distinct (self-explanatory) subfolders per major PEMA pipeline step. In the last subfolder, i.e., subfolder 8, the results are further split into folders per sample. This eases further analysis both within the PEMA framework (e.g., partial re-execution for parameter exploration) and beyond. An extra subfolder is created when an ecological analysis via the “phyloseq” package has been selected.

## Results and Discussion

### Evaluation

To evaluate PEMA, 2 approaches were followed. First, PEMA was benchmarked against mock community datasets. Second, PEMA was used to analyse previously published datasets. PEMA's output was then compared with the original study outcome, as well as with the output of QIIME2, LotuS, Mothur, and Barque (where applicable).

Four mock communities, 1 for each marker gene, were used. With respect to the 16S rRNA marker gene, a mock community of Gohl et al. [45] with 20 different bacterial species was studied. Correspondingly, in the case of the 18S rRNA marker gene, a mock community of Bradley et al. [46] with 12 algal species was used; for the ITS, one of Bakker [47] including 19 different fungal taxa; and for the case of the COI marker gene, a mock community of Bista et al. [48] containing 14 metazoan species. More information on the mock communities, their original studies, and the results of PEMA for various combinations of parameters can be found in Additional File 2: Mock Communities.

Complementary to the mock community evaluation, 2 publicly available datasets from published studies were investigated through PEMA. For the 16S rRNA marker gene, the dataset reported by Pavloudi et al. [49] was used; the original study aimed at investigating the sediment prokaryotic diversity along a transect river–lagoon–open sea. For the COI case, the dataset of Bista et al. [50] was used; this study investigated whether eDNA can be used for the accurate detection of chironomids (a taxonomic group of macroinvertebrates) in a freshwater habitat.

In both approaches, the respective .fastq files were downloaded from the European Nucleotide Archive (ENA) of the European Bioinformatics Institute ENA-(EBI) using “ENA File Downloader version 1.2” [51] and PEMA was run on the in-house HPC cluster.

All analyses were conducted on identical Dell M630 nodes (128 GB RAM, 20 physical Intel Xeon 2.60 GHz cores).

### Mock community evaluation

PEMA was tested against mock communities. An evaluation of its accuracy must capture (i) how many of PEMA's predictions are true (i.e., the percent of correctly assigned taxa among all predicted taxa) and (ii) how many of the taxa existing in the mock community were recovered successfully by PEMA. The precision statistical metric was used to assess the former, and recall, the latter. In addition, the F1-score was used as a combined metric of both precision and recall. Precision is calculated as the ratio of true-positive results (TP) over the total number of true- (TP) and false-positive results (FP) predicted by a model, as follows: precision = TP/(TP + FP); recall is the ratio of TP over the total number of TP and false-negative results (FN): recall = TP/(TP + FN). The F1-score is the precision and recall harmonic mean and is calculated by means of the following formula: F1 = 2 × (precision × recall)/(precision + recall) [52].

Adequate accuracy was achieved when PEMA was used to recover the marker gene–specific mock communities at the genus level. Precision and recall scores of ∼80% or more were observed, with 2 exceptions in precision but also 3 very high scores in recall. Overall the F1-scores ranged from 74% to 86%. A detailed description of the benchmark methodology and statistics analysis is given in Additional File 2: Mock Communities.

Detailed presentation of per-marker-gene–specific mock community recovery via PEMA is provided in the following sections. Several different sets of parameters were chosen for each marker gene. Each marker gene has special features (e.g., length variability, sequence variability), and each Illumina run has its own intrinsic biases (e.g., primers used, PCR protocol); thus, parameter tuning plays a crucial part in metabarcoding analyses.

In an attempt to thoroughly analyse the sequence data from the mock communities, various sets of parameters were tested on the basis of the experimental details of the published studies but also in an exploratory way. Many different parameter settings were tested, especially for the steps of quality trimming of the reads and the OTU clustering/ASV inference. The differences in their output indicate how sensitive this method is, as well as the great need of a mock community in every metabarcoding study—both as a control but also as a “tuning system” for the parameter setting of the pipeline used.

### 16S rRNA

When PEMA was performed with the Swarm v2 algorithm (*d* = 3, strictness = 0.6) without removal of singletons, 18 of the 20 taxa were identified to the genus level and 3 of these even to the species level. There were 2 species that were not found in any of the PEMA runs. According to Gohl et al. [45], there was a discrepancy in the identification of those 2 species that was dependent on the amplification protocol used. It is worth mentioning that as *d* increases, taxa cannot be identified to species level at all; however, FP assignments decrease. Thus, when *d* = 30 and strictness = 0.6 for the KAPA samples, *Enterococcus* was not identified at all; however, PEMA finds its greatest F1 value (at the genus level, see Table 1) as the FP assignments returned are minimized. When PEMA was run using the VSEARCH clustering algorithm, high precision values were returned in all cases (>0.79). However, the recall values were decreased when using Swarm v2 (0.65–0.68).

**Table 1:** Summary benchmark of PEMA marker-gene–specific mock community recovery (precision)

Marker gene	Precision	Recall	F1
16S rRNA	0.81	0.85	0.83
18S rRNA	0.75	0.90	0.82
ITS	0.79	0.94	0.86
COI	0.62	0.93	0.74

### 18S rRNA

When PEMA was performed using the Swarm v2 algorithm (*d* = 1, strictness = 0.5), 3 of 12 community members were identified to species level (*Isochrysis galbana, Nannochloropsis oculata*, and *Thalassiosira pseudonana*), 6 to genus, and the remaining 3 to class; the latter were all the green algae species (Chlorophyta) of the mock community. However, a better F1-score (0.82) was achieved when the class of Chlorophyceae was not found at all (*d* = 1, strictness = 0.3) because the FPs were decreased to only 1. When the VSEARCH algorithm was used, *I. galbana* was identified only to the genus level, the *Nannochloropsis* to the order level (Eustigmatales), and the *Poterioochromonas* genus to its class (Chrysophyceae).

### ITS

When PEMA was performed using the Swarm v2 algorithm (*d* = 20) and targeting the ITS2 region, ASVs from 5 of the 19 species of the mock community were assigned to species level, 10 to genus, 2 to family, and 2 to class level. Contrary to the study by Bakker [47], PEMA identified the genus *Chytriomyces* in all 3 samples, as well as the Ustilaginaceae family. Only 1 FP assignment was recorded. When the CROP algorithm was used, PEMA's output was less accurate; the *Fusarium* species contained in the mock community were not identified further than their family (Nectriaceae). As mentioned by Bakker [47], many reads deriving from the *Fusarium* spp. were not assigned to species level because of the quality-trimming step. In addition, a manually assembled reference database for the taxonomy assignment was used in the initial study, containing only sequences of the mock community species, which biased this step, making the results not directly comparable to our case.

### COI

When PEMA was performed on the *Bista* et al. dataset [48] and using Swarm v2 (*d* = 10), it identified 12 of the 14 species included in the mock community. The sole non-identified species were *Bithynia leachii* and *Anisus vortex*. For *B. leachii* no entry exists in the MIDORI database, version MIDORI_LONGEST_1.1. However, the existence of another species of the genus *Bithynia* was recorded. With respect to *A. vortex*, PEMA returned a high abundance ASV assigned to the *Anisus* genus but with a low confidence level. PEMA managed to identify all the members of the mock community. This includes *Physa fontinalis*, which was originally not designed to be a member of the mock community but, as Bista et al. [48] explain, was recorded owing to cross-contamination. In the case of the COI marker gene, unique sequences with low abundances (singletons or doubletons) often lead to spurious MOTUs/ASVs. Thus, as shown in Additional File 2: Mock Communities, the FP assignments are decreased when these low-abundant sequences are removed; also, the abundance of the assignments (i.e., read counts) retrieved can indicate FP assignments. Thus, TP assignments occur in greater abundance, with hundreds or even thousands of reads—contrary to most of the FP results, whose abundance is <10 read counts. That is mostly for the case of the COI marker gene because eukaryotes are under study; eukaryotes have a great number of copies of this marker gene—different numbers of copies among the different species—and not just a single one as is almost always the case in bacteria. Therefore, assignments with such low abundances should be doubted as TP results in analyses on real datasets.

### Comparison with existing software

PEMA's features were compared with those of mothur [4], QIIME 2 [5], LotuS [6], and Barque [7]. Table 2 presents a detailed comparison among the 4 tools' features in terms of marker gene support, diversity and phylogeny analysis capability, parameter setting and mode of execution, operation system availability, and HPC suitability. As shown, PEMA is equally feature-rich, if not richer in certain feature categories, compared with the other software packages. In particular, PEMA's support for COI marker gene studies is distinctive; 2 methods for taxonomy assignment are supported, and PEMA's easy parameter setting, step-by-step execution, and container distribution render it user and analysis friendly.

**Table 2:**
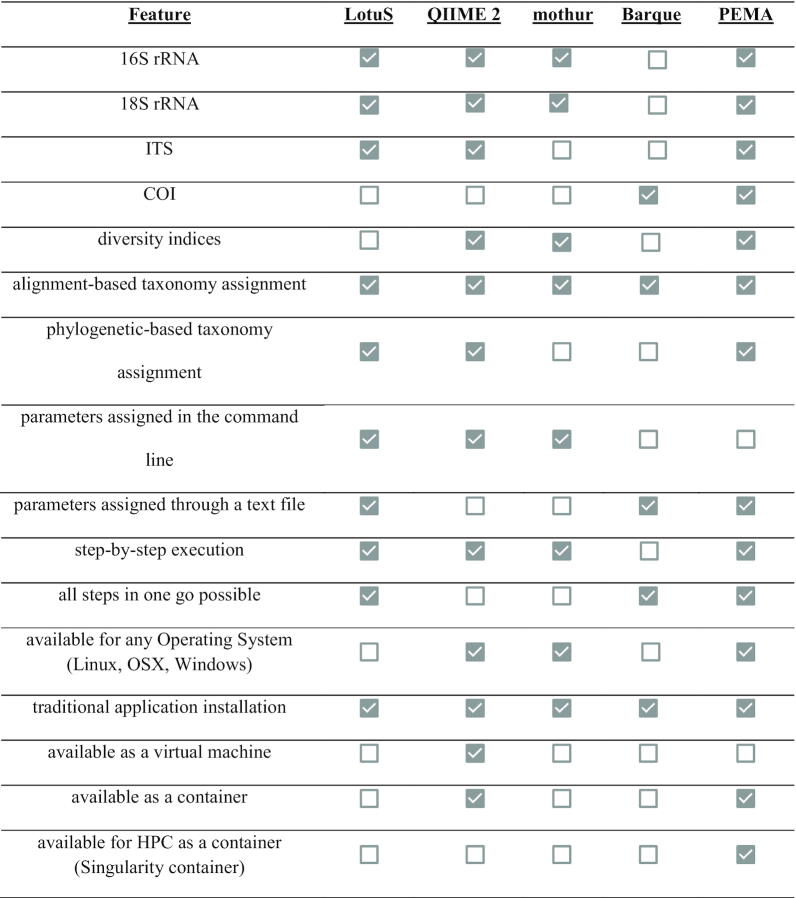
Comparison of the basic features of the different pipelines

### Evaluation on real datasets and against other tools

In the following sections, a comparative study on real datasets of the 16S rRNA and COI marker genes is presented. Analyses using PEMA and the pipelines mentioned above that support each of these 2 marker genes were performed, both with multiple sets of parameters. It is typical for pipelines to invoke a variety of established tools. In many cases, a number of tools are common among different pipelines. Therefore, it is important to stress that such comparisons should not be taken into account strictly; declaring that one pipeline is better than another is not trivial. Potentials and limitations of both the pipelines and the metabarcoding method, as well as the importance of the role of the pipeline user, are underlined in the following sections.

### 16S rRNA marker gene analysis evaluation

To evaluate PEMA's performance, a comparative analysis of the Pavloudi et al. [49] dataset with mothur [4], QIIME 2 [5], LotuS [6], and PEMA was conducted.

It is known that the choice of parameters affects the output of each analysis; therefore, it is expected that different user choices might distort the derived outputs. For this reason and for a direct comparison of the pipelines, we have included all the commands and parameters chosen in the framework of this study in Additional File 1: Supplementary Methods. The results of the processing of the sequences by PEMA are presented in Table S1. All analyses were conducted on identical Dell M630 nodes (128 GB RAM, 20 physical Intel Xeon 2.60 GHz cores). LotuS, mothur, and QIIME 2 operated in a single-thread (core) fashion. PEMA, given the BDS intrinsic parallelization [11], operated with up to the maximum number of node cores (in this case 20).

The execution time and the reported OTU number of each tool are presented in Table 3. LotuS and PEMA resulted in a final number of OTUs comparable to that of Pavloudi et. al [49]. Clearly, owing to PEMA's parallel execution support, the analysis time can be significantly reduced (∼1.5 hours in this case). The execution time depends on the parameters chosen for each software (see Additional File 1: Supplementary Methods).

**Table 3:** OTU predictions and execution time for the different pipelines

			QIIME 2			
Parameter	LotuS	mothur	Deblur	DADA2	PEMA	Pavloudi et al. [49]
No. of OTUs	9,849	142,669	517	1,023	6,028	7,050
Execution time (h)	∼9	∼67*	2.5	∼5	∼1.5	∼26

* (∼56 if the reference database is already built).

**Figure 3:**
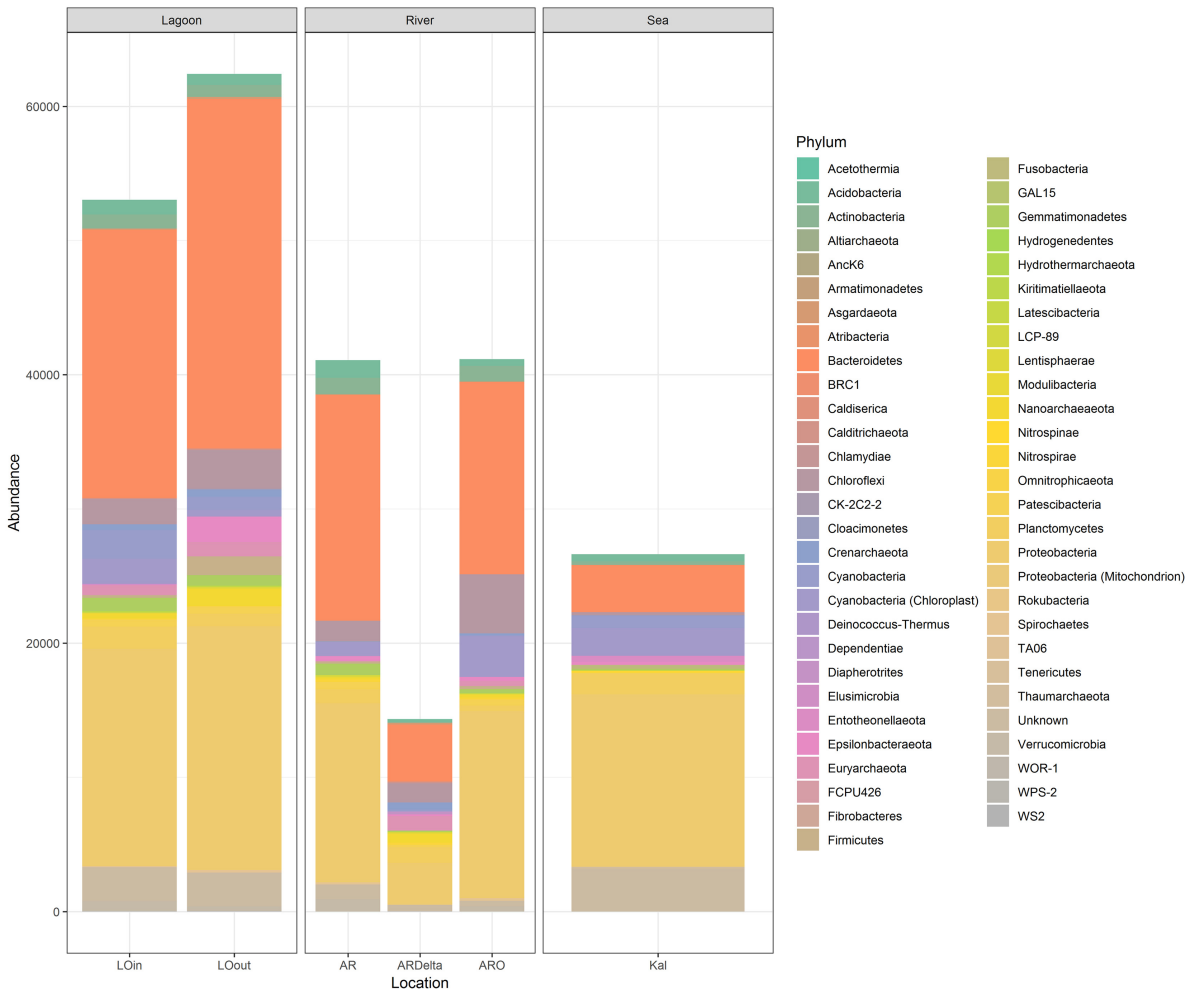
OTU bar plot at the phylum level. Bar plot depicting the taxonomy of the retrieved OTUs from PEMA for the dataset of Pavloudi et al. [49], at the phylum level for the case of the 16S marker gene. AR: Arachthos; ARO: Arachthos Neochori; ARDelta: Arachthos Delta; LOin: Logarou station inside the lagoon; LOout: Logarou station in the channel connecting the lagoon to the gulf; Kal: Kalamitsi.

Owing to the non-full overlap of the sequence reads, mothur resulted in an inflated number of OTUs; thus, it was excluded from further analyses. The results of all the pipelines were analysed with the phyloseq script that is provided with PEMA. The taxonomic assignment of the PEMA-retrieved OTUs is shown in Fig. 3. The phyla that were found in the samples are similar to the ones that were found in the original study [49]. Although the lowest number of OTUs was found in the marine station (Kal) (Supplementary Table S3), which is not in accordance with Pavloudi et. al [49], the general trend of a decreasing number of OTUs with increasing salinity was observed as in the original study (Supplementary Fig. S1). Notably, this result was not observed with the other tested pipelines (Supplementary Table S3). Furthermore, each of the pipelines resulted in a different taxonomic profile (Supplementary Figs S2–S4), with an extreme case of missing the order of Betaproteobacteriales (Supplementary Figs S5–S7).

Moreover, when the PERMANOVA analysis was run for the results of PEMA, LotuS, and DADA2, it was clear that the microbial community composition was significantly different in each of the 3 sampled habitats (i.e., river, lagoon, open sea) (PERMANOVA: F.Model = 7.0718, *P* < 0.001; F.Model = 6.5901, *P* < 0.001; F.Model = 2.2484, *P* < 0.05, respectively), which is in accordance with Pavloudi et al. [49]. However, this was not the case with Deblur (PERMANOVA: *P* > 0.05). Overall, PEMA's output is in accordance with the original study [49], and seen through this perspective PEMA performed equally well with the other tested pipelines, along with having the shortest execution time.

### COI marker gene analysis evaluation

Bista et al. [50] created 2 COI libraries of different sizes: COIS (235- bp amplicon size) and COIF (658 -bp amplicon size). The sequencing reads of COIS were selected for PEMA's evaluation; the COIF sequencing read pairs had no overlap so as to be merged and therefore were not considered appropriate for the analysis.

As previously, PEMA's performance was evaluated through a comparative analysis of the Bista et al. [50] dataset with Barque [7]; the commands and parameters chosen can be found in Additional File 1: Supplementary Methods. Regarding the creation of the MOTU table, in the Bista et al. [50] study VSEARCH [17] was used with a clustering at 97% similarity threshold. Afterwards, the BLAST+ (megablast) algorithm [53] was used against a manually created database including all NCBI GenBank COI sequences of length >100  bp (June 2015) while excluding environmental sequences and higher taxonomic level information [50]. As discussed in the publication, this approach resulted in 138 unique MOTUs of which 73 were assigned to species level. For PEMA's evaluation, the chosen clustering algorithm was Swarm v2, using different options for the cluster radius (*d*) parameter (Table 4); according to Mahé et al. [19], this is the most important parameter because it affects the number of MOTUs that are being created. The resulting MOTUs were classified against the MIDORI reference database [26] using RDPClassifier [25]. The results of the processing of the sequences are reported in Supplementary Table S3. For the case of Barque, the BOLD Database was used [54].

**Table 4:** PEMA's^a^ output and execution time

Parameter	*d* = 1	*d* = 2	*d* = 3	*d* = 10	*d* = 13
MOTUs after pre-process and clustering steps	83,791	59,833	33,227	7,384	4,829
MOTUs after chimera removal	80,347	57,863	32,539	7,339	4,796
Non-singleton MOTUs	6,381	4,947	2,658	1,914	1,634
Assigned species	62	83	86	86	84
Execution time (h)	2:01:35	2:09:49	1:51:44	2:17:26	2:31:15

a PEMA's output and execution time (using a 20-core node) for different values of Swarm's *d* parameter.

As shown in Table 4, PEMA resulted in 83 species-level MOTUs with a cluster radius (*d*) of 2, which is similar to the findings of the published study (i.e., 73 species). Although both the clustering algorithm and the taxonomy assignment methods were different between the original [50] and the present study, the results regarding the number of unique species present in the samples are in agreement to a considerable extent.

The computational time required by PEMA for the completion of the analysis is also reported in Table 4. Regardless of the value of the *d* parameter, all analyses were completed in ∼2 hours, i.e., fast enough to allow parameter testing and customization. Regarding Barque, the analysis resulted in the identification of 51 species-level MOTUs and was concluded in 15 minutes. This difference is due to the error correction step of PEMA (BayesHammer algorithm [32]), which plays an important part in the enhanced results that PEMA returns, but it also requires a certain computational time; Barque does not have an analogous step, and therefore its overall execution time is shorter.

PEMA performed better than Barque at identifying taxa that were included in the positive control contents of the published study (Table 5).

**Table 5:** Comparison of the taxonomy of retrieved MOTUs among PEMA, Barque, and the positive controls of Bista et al. [50]

Barque	PEMA	Bista et al. [50]
*Ablabesmyia monilis* *	*Ablabesmyia monilis* *	*Ablabesmyia monilis*
	*Crangonyx pseudogracilis* *	*Crangonyx pseudogracilis*
	*Radix* sp.*	*Radix* sp.
	Chironomidae sp.*	Chironomidae sp.
	*Ancylus* sp.**	*Ancylus fluviatilis*
	*Athripsodes aterrimus, Athripsodes cinereus* **	*Athripsodes albifrons*
*Chironomus anthracinus* **	*Chironomus* sp., Chironomus anthracinus, Chironomus pseudothummi, Chironomus riparius**	*Chironomus tentans*
*Polypedilum sordens* **		*Polypedilum nubeculosum*
*Athripsodes aterrimus* **		*Athripsodes albifrons*

* Taxonomies identical to the published study (species level).

** Taxonomies identical to the published study (genus level).

### OTU clustering vs ASV inference

There is an ongoing discussion about whether ASVs exceed OTUs. The strongest argument to this end is that ASVs are real biological sequences. Hence, they can be compared between different studies in a straightforward way; considered as consistent labels. In comparison, *de novo* OTUs are constructed, or “clustered,” with respect to the emergent features of each specific dataset. Therefore, OTUs defined in 2 different datasets cannot be directly compared.

However, the OTU concept is not compulsorily related to the clustering approach; it is widely used to describe results based on its biological meaning but it does not imply clustering. In addition, according to Callahan et al. [15], “ASV methods infer the biological sequences in the sample prior to the introduction of amplification and sequencing errors, and distinguish sequence variants differing by as little as one nucleotide.” As a result, ASVs could be considered as OTUs of higher resolution.

It is due to this concept confusion that algorithms whose rationale is considerably closer to the variant-based approach are still considered as OTU clustering algorithms [15]. Swarm v2 produces all possible “microvariants” of an amplicon to implement an exact-string comparison [19]. Furthermore, real biological sequences, “clouds of microvariants,” are produced as its output, which can be used for comparisons between different studies. Thus, Swarm v2 can be considered as an ASV-inferring algorithm.

Traditional clustering methods have certain limitations such as arbitrary global clustering thresholds and centroid selection because they depend on the input order and are time-consuming, etc. [55], which variant-based approaches manage to address. However certain algorithms for OTU clustering such as VSEARCH have been proven to be especially reliable, and they are widely used by many researchers. Furthermore, ASVs intend to improve taxonomic resolution; however, a vast number of inferred ASVs [56] can lead to inflation of diversity estimates, especially in the case of microbial communities, thus making the analysis even more complicated.

ASV or OTU approaches are supported by PEMA, although we have found that similar ecological results are produced by both these methods, as also suggested by Glassman and Martiny [57].

### Beyond environmental ecology, ongoing and future work

PEMA is mainly intended to support eDNA metabarcoding analysis and be directly applicable to next-generation biodiversity/ecological assessment studies. Given that community composition analysis may also serve additional research fields, e.g., microbial pathology, the potential impact of such pipelines is expected to be much higher. Ongoing PEMA work focuses on serving a wide scientific audience and on making it applicable to more types of studies. The easy set-up and execution of PEMA allows users to work closely with national and European HPC/e-infrastructures (e.g., ELIXIR Greece [58], LifeWatch ERIC [59], EMBRC ERIC [60]). To that end and in a mid-term perspective, a CWL version of PEMA will be explored. The aim of this effort is to reach out to a wider scientific audience and address both their ongoing as well as future analysis needs.

By supporting the analysis of the most commonly used marker genes for Bacteria and Archaea (16S rRNA), Fungi (ITS), and Metazoa (COI/18S rRNA), a holistic biodiversity assessment approach is now possible through PEMA and eDNA metabarcoding; although, from a mid-term perspective, it is our intention to allow ad hoc and in-house databases to be used as reference for the taxonomy assignment.

## Conclusions

PEMA is an accurate, execution-friendly and fast pipeline for eDNA metabarcoding analysis. It provides a per-sample analysis output, different taxonomy assignment methods, and graphics-based biodiversity/ecological analysis. This way, in addition to (M)OTU/ASV calling, it provides users with both an informative study overview and detailed result snapshots.

Thanks to a nominal number of installation and execution commands required for PEMA to be set and run, it is considered essentially user friendly. In addition, PEMA's strategic choice of a single parameter file, implementation programming language, and multiple container-type distribution grant it speed (running in parallel), on-demand partial pipeline enactment, and provision for HPC-system–based sharing.

All the aforementioned features render PEMA attractive for biodiversity/ecological assessment analyses. By supporting the analysis of the most commonly used marker genes for Prokaryotes (Bacteria and Archaea), as well as Eukaryotes (Fungi and Metazoa), PEMA allows assessment of biodiversity in different levels of biodiversity. Applications may mainly concern environmental ecology, with possible extensions to such fields as microbial pathology and gut microbiome, in line with modern research needs, from low volume to big data.

## Availability of Supporting Source Code and Requirements

Project name: PEMA

Project home page: https://github.com/hariszaf/pema

Dockerized version: https://hub.docker.com/r/hariszaf/pema

Singularity image: https://singularity-hub.org/collections/2295

Operating system(s): Platform independent

Programming language: BigDataScript

Other requirements: Singularity (in case of HPC use)

License: GNU GPLv3. For third-party components separate licenses apply. See Additional File 1 for a list of tools invoked by PEMA and their respective licenses.

bio.tools id: PEMA

RRID:SCR_017676

## Availability of Supporting Data and Materials

The sequence data that support the findings of this study, with respect to the mock community–based evaluation, are available in the European Nucleotide Archive (ENA) with the following study accession numbers—for the 16S, 18S rRNA, ITS, and COI marker genes, respectively:

PRJNA305443 (https://www.ebi.ac.uk/ena/browser/view/PRJNA305443),

PRJNA314977 (https://www.ebi.ac.uk/ena/browser/view/PRJNA314977),

PRJNA377530 (https://www.ebi.ac.uk/ena/browser/view/PRJNA377530), and

PRJEB23036 (https://www.ebi.ac.uk/ena/browser/view/PRJEB23036)

The real datasets used are also available in ENA:

PRJEB20211 (http://www.ebi.ac.uk/ena/data/view/PRJEB20211) and

PRJEB13009 (https://www.ebi.ac.uk/ena/data/view/PRJEB13009).

An archived version of the code and supporting data is also available via the *GigaScience* database GigaDB [61].

## Supplementary Material

giaa022_GIGA-D-19-00397_Original_Submission

giaa022_GIGA-D-19-00397_Revision_1

giaa022_Response_to_Reviewer_Comments_Original_Submission

giaa022_Reviewer_1_Report_Original_Submission Johan Andre Pansu -- 12/17/2019 Reviewed

giaa022_Supplement_Files

## Additional Files

**Additional File 1:** Supplementary Methods: Description of tools invoked by PEMA and their licences. Description of the commands, along with their parameters, used to run PEMA, mothur, LotuS, and QIIME 2.

**Additional File 2:** Mock Communities: Details about the mock communities chosen and their corresponding studies, as well as the returned output of PEMA for each for a number of sets of parameters.

**Supplementary Table S1:** Number of sequences after each pre-processing step for the case of 16S rRNA gene.

**Supplementary Table S2:** Diversity indices of the samples.

**Supplementary Figure S1:** Linear regression between the number of OTUs (averaged per sampling station) and the salinity of the sampling stations. L: Lagoon; S: Sea; R: River; AR: Arachthos; ARO: Arachthos Neochori; ARDelta: Arachthos Delta; LOin: Logarou station inside the lagoon; LOout: Logarou station in the channel connecting the lagoon to the gulf; Kal: Kalamitsi.

**Supplementary Figure S2:** Bar plot depicting the taxonomy of the retrieved OTUs from LotuS at the phylum level.

**Supplementary Figure S3:** Bar plot depicting the taxonomy of the retrieved OTUs from QIIME 2 using Deblur at the phylum level.

**Supplementary Figure S4:** Bar plot depicting the taxonomy of the retrieved OTUs from QIIME 2 using DADA2 at the phylum level.

**Supplementary Figure S5:** Bar plot depicting the taxonomy of the retrieved OTUs from LotuS at the class of Betaproteobacteriales.

**Supplementary Figure S6:** Bar plot depicting the taxonomy of the retrieved OTUs from QIIME 2 using Deblur at the class of Betaproteobacteriales.

**Supplementary Figure S7:** Bar plot depicting the taxonomy of the retrieved OTUs from PEMA at the class of Betaproteobacteriales.

**Supplementary Table S3:** Number of sequences after each pre-processing step for the case of COI, dataset from Bista et al. [50].

## Abbreviations

BDS: BigDataScript; bp: base pairs; COI: cytochrome oxidase subunit 1; CREST: Classification Resources for Environmental Sequence Tags; CROP: Clustering 16S rRNA for OTU Prediction; CWL: Common Workflow Language; eDNA: environmental DNA; FN: false negative; FP: false positive; HCMR: Hellenic Centre for Marine Research; HPC: high-performance computing; iTOL: Interactive Tree of Life; MAFFT: Multiple Alignment using Fast Fourier Transform; MCMC: Markov chain Monte Carlo; MOTU: molecular operational taxonomic unit (used for eukaryotes); MSA: multiple sequence alignment; NCBI: National Center for Biotechnology Information; OTU: operational taxonomic unit (used for prokaryotes); PaPaRa: Parsimony-based Phylogeny-Aware Read Alignment; PEMA: Pipeline for Environmental DNA Metabarcoding Analysis; PERMANOVA: permutational multivariate analysis of variance; RAM: random access memory; RAxML: Randomized Axelerated Maximum Likelihood; rRNA: ribosomal RNA; SPAdes: St. Petersburg genome assembler; SSU: small subunit; TP: true positive.
