# Corrigendum to: PEMA: a flexible pipeline for environmental DNA metabarcoding analysis of the 16S/18S ribosomal RNA, ITS, and COI marker genes

**DOI:** 10.1093/gigascience/giaa150

**Published:** 2020-12-21

**Authors:** Haris Zafeiropoulos, Ha Quoc Viet, Katerina Vasileiadou, Antonis Potirakis, Christos Arvanitidis, Pantelis Topalis, Christina Pavloudi, Evangelos Pafilis

**Affiliations:** Institute of Marine Biology, Biotechnology and Aquaculture (IMBBC), Hellenic Centre for Marine Research (HCMR), Former U.S. Base of Gournes P.O. Box 2214, 71003, Heraklion, Crete, Greece; Institute of Marine Biology, Biotechnology and Aquaculture (IMBBC), Hellenic Centre for Marine Research (HCMR), Former U.S. Base of Gournes P.O. Box 2214, 71003, Heraklion, Crete, Greece; Institute of Marine Biology, Biotechnology and Aquaculture (IMBBC), Hellenic Centre for Marine Research (HCMR), Former U.S. Base of Gournes P.O. Box 2214, 71003, Heraklion, Crete, Greece; Charles University, Department of Ecology, Faculty of Science, Viničná 7, CZ-12844, Prague, Czech Republic; Institute of Marine Biology, Biotechnology and Aquaculture (IMBBC), Hellenic Centre for Marine Research (HCMR), Former U.S. Base of Gournes P.O. Box 2214, 71003, Heraklion, Crete, Greece; Institute of Marine Biology, Biotechnology and Aquaculture (IMBBC), Hellenic Centre for Marine Research (HCMR), Former U.S. Base of Gournes P.O. Box 2214, 71003, Heraklion, Crete, Greece; LifeWatch ERIC, Plaza Espana SN, SECTOR II-III 41013, Seville, Spain; Institute of Molecular Biology and Biotechnology (IMBB), Foundation for Research and Technology (FORTH), Foundation for Research and Technology – Hellas, N. Plastira 100, GR-70013, Heraklion, Crete, Greece; Institute of Marine Biology, Biotechnology and Aquaculture (IMBBC), Hellenic Centre for Marine Research (HCMR), Former U.S. Base of Gournes P.O. Box 2214, 71003, Heraklion, Crete, Greece; Institute of Marine Biology, Biotechnology and Aquaculture (IMBBC), Hellenic Centre for Marine Research (HCMR), Former U.S. Base of Gournes P.O. Box 2214, 71003, Heraklion, Crete, Greece

In the originally published version of this manuscript, the following affiliation was omitted from first author Haris Zafeiropoulos's affiliation: “Department of Biology, University of Crete, Voutes University Campus, Heraklion, Greece.” This has now been corrected online.

