# Supplementary material for: PEMA: a flexible Pipeline for Environmental DNA Metabarcoding Analysis of the 16S/18S ribosomal RNA, ITS, and COI marker genes
Source: Gigascience. 2020 Mar 12;9(3):giaa022. doi: 10.1093/gigascience/giaa022 (PMC7066391; doi:10.1093/gigascience/giaa022)
Supplement: giaa022_GIGA-D-19-00397_Original_Submission [file giaa022_giga-d-19-00397_original_submission.pdf]

GigaScience

PEMA: a flexible Pipeline for Environmental DNA Metabarcoding Analysis of the 16S/18S rRNA, ITS and COI marker genes

--Manuscript Draft--

**Manuscript Number:**	GIGA-D-19-00397
**Full Title:**	PEMA: a flexible Pipeline for Environmental DNA Metabarcoding Analysis of the 16S/18S rRNA, ITS and COI marker genes
**Article Type:**	Technical Note
**Funding Information:**	General Secretariat for Research and Technology (241) — Dr Evangelos Pafilis
**Abstract:**	Background: Environmental DNA (eDNA) and metabarcoding allow the identification of a mixture of species individuals and launch a new era in bio- and eco-assessment. A great number of steps are required to obtain taxonomically assigned matrices from raw data. For most of these, a plethora of tools are available; each tool's execution parameters need to be tailored to reflect each experiment's idiosyncrasy. Adding to this complexity, the computation capacity of High Performance Computing systems is frequently required for such analyses. To address the aforementioned difficulties, bioinformatic pipelines need to combine state-of-the art technologies and algorithms with an easy to get-set-use framework, allowing researchers to tune each study. Software containerization technologies ease the sharing and running of software packages across operating systems; thus, they strongly facilitate pipeline development and usage. Likewise are programming languages specialized for big data pipelines, incorporating features like roll-back checkpoints and on-demand partial pipeline execution.<br>Findings: PEMA is a containerized assembly of key metabarcoding analysis tools with a low effort in setting up, running and customizing to researchers' needs. Based on third party tools, PEMA performs read pre-processing, (M)OTUs clustering, ASV inference, and taxonomy assignment for 16S and 18S rRNA as well as ITS and COI marker gene data. Due to its simplified parameterisation and checkpoint support, PEMA allows users to explore alternative algorithms for specific steps of the pipeline without the need of a complete re-execution. PEMA was evaluated against both mock communities and previously published datasets and achieved comparable quality results.<br>Conclusions: An HPC-based approach was used to develop PEMA, however it can be used in personal computers as well. Given its time-efficient performance and its quality results, it is suggested that PEMA can be used for accurate eDNA metabarcoding analysis, thus enhancing the applicability of next-generation biodiversity assessment studies.
**Corresponding Author:**	Haris Zafeiropoulos<br>Hellenic Centre for Marine Research<br>Heraklion, Irakleio GREECE
**Corresponding Author Secondary Information:**	
**Corresponding Author's Institution:**	Hellenic Centre for Marine Research
**Corresponding Author's Secondary Institution:**	
**First Author:**	Haris Zafeiropoulos
**First Author Secondary Information:**	
**Order of Authors:**	Haris Zafeiropoulos
	Viet Ha Quoc
	Katerina Vasileiadou
	Antonis Potirakis

Powered by Editorial Manager® and ProduXion Manager® from Aries Systems Corporation

	Christos Arvanitidis
	Pantelis Topalis
	Christina Pavloudi
	Evangelos Pafilis
**Order of Authors Secondary Information:**	
**Additional Information:**	
**Question**	**Response**
Are you submitting this manuscript to a special series or article collection?	No
**Experimental design and statistics**<br><br>Full details of the experimental design and statistical methods used should be given in the Methods section, as detailed in our Minimum Standards Reporting Checklist. Information essential to interpreting the data presented should be made available in the figure legends.<br><br>Have you included all the information requested in your manuscript?	Yes
**Resources**<br><br>A description of all resources used, including antibodies, cell lines, animals and software tools, with enough information to allow them to be uniquely identified, should be included in the Methods section. Authors are strongly encouraged to cite Research Resource Identifiers (RRIDs) for antibodies, model organisms and tools, where possible.<br><br>Have you included the information requested as detailed in our Minimum Standards Reporting Checklist?	Yes
**Availability of data and materials**<br><br>All datasets and code on which the conclusions of the paper rely must be either included in your submission or	Yes

deposited in publicly available repositories (where available and ethically appropriate), referencing such data using a unique identifier in the references and in the “Availability of Data and Materials” section of your manuscript.<br><br>Have you have met the above requirement as detailed in our Minimum Standards Reporting Checklist?	

Powered by Editorial Manager® and ProduXion Manager® from Aries Systems Corporation

Manuscript Click here to download Manuscript PEMA_manuscript .docx

PEMA: a flexible Pipeline for Environmental DNA Metabarcoding Analysis of the 16S/18S rRNA, ITS and COI marker genes

Haris Zafeiropoulos*
Institute of Marine Biology, Biotechnology and Aquaculture (IMBBC), Hellenic Centre for Marine Research (HCMR), Heraklion, Greece
e-mail: haris-zaf@hcmr.gr
*** Corresponding author**

Ha Quoc Viet
Institute of Marine Biology, Biotechnology and Aquaculture (IMBBC), Hellenic Centre for Marine Research (HCMR), Heraklion, Greece
e-mail: qvha@free.fr

Katerina Vasileiadou
Charles University, Prague, Czech Republic
Institute of Marine Biology, Biotechnology and Aquaculture (IMBBC), Hellenic Centre for Marine Research (HCMR), Heraklion, Greece
e-mail: kvasileiadou@hcmr.gr

Antonis Potirakis
Institute of Marine Biology, Biotechnology and Aquaculture (IMBBC), Hellenic Centre for Marine Research (HCMR), Heraklion, Greece
e-mail: potant@hcmr.gr

Christos Arvanitidis
Institute of Marine Biology, Biotechnology and Aquaculture (IMBBC), Hellenic Centre for Marine

Research (HCMR), Heraklion, Greece
e-mail: arvanitidis@hcmr.gr

Pantelis Topalis
Institute of Molecular Biology and Biotechnology (IMBB), Foundation for Research and Technology (FORTH), Heraklion, Greece
e-mail: topalis@imbb.forth.gr

Christina Pavloudi
Institute of Marine Biology, Biotechnology and Aquaculture (IMBBC), Hellenic Centre for Marine Research (HCMR), Heraklion, Greece
e-mail: cpavloud@hcmr.gr

Evangelos Pafilis
Institute of Marine Biology, Biotechnology and Aquaculture (IMBBC), Hellenic Centre for Marine Research (HCMR), Heraklion, Greece
e-mail: pafilis@hcmr.gr

Abstract

Background: Environmental DNA (eDNA) and metabarcoding allow the identification of a mixture of species individuals and launch a new era in bio- and eco-assessment. A great number of steps are required to obtain taxonomically assigned matrices from raw data. For most of these, a plethora of tools are available; each tool's execution parameters need to be tailored to reflect each experiment's idiosyncrasy. Adding to this complexity, the computation capacity of High Performance Computing systems is frequently required for such analyses. To address the aforementioned difficulties, bioinformatic pipelines need to combine state-of-the art technologies and algorithms with an easy to get-set-use framework, allowing researchers to tune each study. Software containerization technologies ease the sharing and

running of software packages across operating systems; thus, they strongly facilitate pipeline development and usage. Likewise are programming languages specialized for big data pipelines, incorporating features like roll-back checkpoints and on-demand partial pipeline execution.

Findings: PEMA is a containerized assembly of key metabarcoding analysis tools with a low effort in setting up, running and customizing to researchers' needs. Based on third party tools, PEMA performs read pre-processing, (M)OTUs clustering, ASV inference, and taxonomy assignment for 16S and 18S rRNA as well as ITS and COI marker gene data. Due to its simplified parameterisation and checkpoint support, PEMA allows users to explore alternative algorithms for specific steps of the pipeline without the need of a complete re-execution. PEMA was evaluated against both mock communities and previously published datasets and achieved comparable quality results.

Conclusions: An HPC-based approach was used to develop PEMA, however it can be used in personal computers as well. Given its time-efficient performance and its quality results, it is suggested that PEMA can be used for accurate eDNA metabarcoding analysis, thus enhancing the applicability of next-generation biodiversity assessment studies.

Keywords:

Pipeline, Container, Docker, Singularity, High Performance Computing, HPC, eDNA, metabarcoding

Background

Environmental DNA (eDNA) metabarcoding inaugurates a new era in bio- and eco-monitoring [1]. eDNA, i.e. genetic material obtained directly from environmental samples (soil, sediment, water, etc.) without any obvious signs of biological source material [2], and metabarcoding, a DNA barcoding method that allows the identification of a mixture of organisms, attempt to turn the page into the way biodiversity is perceived and monitored [3]. Their combination is considered to be a holistic approach that, once standardized, allows for higher detection capacity and at a lower cost compared to conventional methods of biodiversity assessment. However, from the raw reads sequence files to an amplicon study analysis results, the

bioinformatics analysis required can be troublesome for many researchers.

Well-established pipelines are available to process metabarcoding data for the case of 16S and 18S rRNA marker genes and bacterial communities (e.g. mothur [4], QIIME 2 [5], LotuS [6]). However, certain limitations accompany each of those and occasionally they can be far from easy-to-use pieces of software. Moreover, there is a great need for similarly straightforward and benchmarked approaches for the analysis of other marker genes. With respect to the COI marker gene, a number of pipelines have been implemented (e.g. Barque [7], ScreenForBio [8] etc.). However, there is still the need for a fast, easy-to-install and easy-to-use pipeline for the COI marker gene, as well as for the ITS.

The pipelines mentioned above, although entrenched, they still suffer from a series of hurdles: technical difficulties in installation and usage, strict limitations in setting parameters for the algorithms invoked, incompetence in partial re-execution of an analysis, are among the most prominent.

Moreover, given the computational demands of such analyses, access to High Performance Computing (HPC) systems might be mandatory, for example, to process studies with large number of samples. This is rather timely given the ongoing investment of national and international efforts (for example [9]) to serve the broad biological community via commonly accessible infrastructures.

PEMA is an open-source pipeline that bundles state-of-the-art bioinformatic tools for all necessary steps of amplicon analysis and aims to address the issues mentioned above. It is designed for paired-end sequencing studies and is implemented in the BigDataScript (BDS) [10] programming language. BDS's *ad hoc* task parallelism and task synchronization, supports heavyweight computation which PEMA inherits. In addition, BDS supports *checkpoint* files that can be used for partial re-execution and crash recovery of the pipeline. PEMA builds on this feature to serve tool and parameter exploratory customization for optimal metabarcoding analysis fine tuning. Switching effortlessly between (Molecular) Operational Taxonomic Units ((M)OTUs) clustering and Amplicon Sequence Variants (ASVs) inference algorithms is a pertinent example. Finally, via software containerization technologies such as Docker [11] and Singularity [12], with the latter being HPC-centered, PEMA is distributed in an easy to download and install fashion on a range of systems from regular computers, to cloud or HPC environments.

From the biology perspective, monitoring the whole biodiversity in general, in all its different levels, is of

great importance. As there is not a single marker gene to detect all taxa, researchers need to use different genes targeting each great taxonomy group separately [13]. To that end, PEMA supports the metabarcoding analysis of both prokaryotic communities, based on the 16S rRNA marker gene, and eukaryotic ones, based on the ITS (for Fungi), and COI and 18S rRNA (for Metazoa) marker genes [13]. As High Throughput Sequencing (HTS) data become more and more accurate, ASVs, i.e. marker gene amplified sequence reads that differ in at least one nucleotide to each other, become easier to resolve [14]. The use of ASVs instead of OTUs has been suggested [14], however the choice for which approach to use should rely on each study's objective(s) [15].

PEMA supports both OTU clustering and ASV inference for all marker genes (see "OTU clustering *vs* ASV inference" in the "Results and discussion" section). Two clustering algorithms, VSEARCH [16] and CROP [17], are employed for the clustering of reads in (M)OTUs; the former for the case of the 16S/18S rRNA marker genes, the latter for the case of COI and ITS. Swarm v2 [18] allows ASV inference in all cases.

Taxonomic assignment is performed in an alignment-based approach, making use of the CREST LCAClassifier [19] and the Silva database [20] for the case of 16S and 18S rRNA marker genes; the Unite database [21] is used for the ITS gene. In the 16S marker gene case, phylogeny-based assignment is also supported, based on RAxML-ng [22], EPA-ng [23] and Silva [20]. For the COI marker gene, the RDPClassifier [24] and the MIDORI database [25] are used for the taxonomic assignment. In addition, ecological and phylogenetic analysis are facilitated via the "phyloseq" R package [26].

All the pipeline- and third-party-module-controlling parameters are defined in a plain *parameter-value pair* text file. Its straightforward format eases the analysis fine tuning, complementary to the aforementioned *checkpoint* mechanism. A tutorial about PEMA and installation guidance can be found on PEMA's GitHub repository (https://github.com/hariszaf/pema).

Implementation

PEMA's architecture comprises four main parts taking place in tandem (Figure 1). Detailed description of the tools invoked by PEMA and their licences is included in Additional file 1: Supplementary Methods.

Part 1: Quality control and pre-processing of raw data

Before all else, FastQC [27] is used to obtain an overall read-quality summary; the visual inspection of each sample's quality may recommend to remove those with utterly poor quality and run again the analysis. To correct the errors produced by the sequencer, PEMA incorporates a number of tools. Trimmomatic [28] implements a series of trimming steps, namely: either to remove parts of the sequences corresponding to the adapters or the primers, or to trim and crop parts of the reads, or even remove a read completely, when it fails to reach the quality filtering standards set by the user. Cutadapt [29] is used additionally for the case of ITS to address the variability in length of this marker gene (see Additional file 1: Supplementary Methods). BayesHammer [30], an algorithm of the SPAdes assembly toolkit [31], revises incorrectly called bases. PANDAseq [32] assembles the overlapping paired-end reads and then the 'obiuniq' program of OBITools [33] groups all the identical sequences in every sample, keeping a track of their abundances. The VSEARCH package [16] is invoked for the chimera removal.

Part 2: (M)OTUs clustering and ASV inference

Quality controlled and processed sequences are subsequently clustered into (M)OTUs or treated as input for inferring ASVs. For the case of 16S and 18S rRNA marker genes, VSEARCH [16] is used for the case of OTU clustering, while ASVs can be identified by the Swarm v2 algorithm [18]. VSEARCH is an accurate and fast tool that can handle large datasets; at the same time it is a great alternative of USEARCH [34] as it is distributed under an open source license.

For the ITS and COI marker genes, CROP [17], an unsupervised probabilistic Bayesian clustering algorithm that models the clustering process using Birth-death Markov chain Monte Carlo (MCMC). The CROP clustering algorithm is adjusted by a series of parameters need to be tuned by the user (namely *b*, *e* and *z*). These parameters depend on specific dataset properties like the length and the number of reads. PEMA, automatically adjusts *b*, *e* and *z* by collecting such information and applying the CROP recommended parameter-setting rules [17]. ASV inference is conducted by Swarm v2 [18] in this case too.

As the Swarm v2 algorithm is not affected by chimeras (F. Mahé, personal communication), when Swarm v2 is selected, chimera removal occurs after the clustering (see Additional file 1: Supplementary Methods: Swarm v2). This leads to a computational time gain as chimeras are sought among ASVs, instead of ungrouped reads.

Last, any singletons, i.e. sequences with only one read, occurring after the (M)OTU clustering or the ASV inference, may be removed according to the user's parameter settings.

Part 3: Taxonomy assignment

Alignment-based taxonomy assignment is supported for all marker gene analyses. In the case of the 16S/18S rRNA and ITS marker genes, the LCAClassifier algorithm of the CREST set of resources and tools [19], is used together with the Silva [20] and the Unite [21] database, respectively, to assign taxonomy to the OTUs. Two versions of Silva are included in PEMA: 128 (Sept 29, 2016) and 132 (Dec 13, 2017). As classifiers need first to be trained for each database they use, for future Silva [20] versions new PEMA versions will be available.

For the COI marker gene, PEMA uses the RDPClassifier [24] and the MIDORI reference database [25] to assign taxonomy of the MOTUs. The MIDORI database contains quality controlled metazoan mitochondrial gene sequences from GenBank [35].

Intended primarily for studies from less explored environments, phylogeny-based assignment is available for 16S rRNA marker gene data. PEMA maps OTUs to a custom reference tree of 1000 Silva-derived consensus sequences (created using RAxML-ng [22] and gappa (phat algorithm) [36], Figure 2A). PaPaRa [37] and EPA-ng [23] combine the OTU clustering output and the reference tree to produce a phylogeny-aware alignment and map the 16S rRNA OTUs to the custom reference tree. Beyond the context of PEMA, users may visualize the output with tree viewers like iTOL [38] (Figure 2B).

Part 4: Ecological downstream analysis of the taxonomy assigned (M)OTU/ASV tables

PEMA's major output is either an (M)OTUor an ASV table with the assigned taxonomies and the abundances of each taxon in every sample. For each sample of the analysis, a subfolder containing statistics

about the quality of its reads, as well as the taxonomies and their abundances, is also returned. Via the "phyloseq" R package [26], downstream ecological analysis of the taxonomically assigned OTUs or ASVs is supported. This includes alpha- and beta-diversity analysis, taxonomic composition, statistical comparisons and calculation of correlations between samples.

When selected, in addition to the phyloseq's [26] output, a Multiple Sequence Alignment (MSA) and a phylogenetic tree of the OTU/ASVs retrieved can be returned; for the MSA, the MAFFT [39] aligner is invoked while the latter is being built by RAxML-ng [22].

PEMA container-based installation

An easy way of installing PEMA is via its containers. A dockerized PEMA version is available at https://hub.docker.com/r/hariszaf/pema. Singularity users can *pull* the PEMA image from https://singularity-hub.org/collections/2295. Between the two containers, the Singularity-based one is recommended for HPC environments due to Singularity's improved security and file accessing properties [40]. For detailed documentation, visit https://github.com/hariszaf/pema.

PEMA output

All PEMA-related files (i.e. intermediate files, final output, *checkpoint* files and per-analysis-parameters) are grouped in distinct (self-explanatory) subfolders per major PEMA pipeline step. In the last subfolder, i.e. subfolder 8, the results are further split in folders per sample. This eases further analysis both within the PEMA framework (like partial re-execution for parameter exploration) or beyond. An extra subfolder is created when an ecological analysis via the "phyloseq" package has been selected.

Results and discussion

Evaluation

To evaluate PEMA, two approaches were followed. First, PEMA was benchmarked against mock community datasets. Second, PEMA was used to analyse previously published datasets. PEMA's output was then compared with the original study outcome as well as with the output of QIIME2, Lotus, Mothur

and Barque (where applicable).

Four mock communities, one for each marker gene were used. With respect to the 16S rRNA marker gene, a mock community of *Gohl* et al. [41] with 20 different bacterial species was studied. Correspondingly, in the case of 18S rRNA marker gene, a mock community of *Bradley* et al. [42] with 12 algal species was used; for the ITS, one of *Bakker* [43] including 19 different fungal taxa and for the case of the COI marker gene, a mock community of *Bista* et al. [44] containing 14 metazoan species. More information on the mock communities, their original studies and the results of PEMA for various combinations of parameters can be found in Additional File 2: Mock Communities.

Complementary to the mock community evaluation, two publicly available datasets from published studies were investigated through PEMA. For the 16S rRNA marker gene, the dataset reported by *Pavloudi* et al. [45] was used; the original study aimed at investigating the sediment prokaryotic diversity along a transect river-lagoon-open sea. For the COI case, the one of *Bista* et al. [46] was used; in this study it was investigated whether eDNA can be used for the accurate detection of chironomids (a taxonomic group of macroinvertebrates) in a freshwater habitat.

In both approaches, the respective .fastq files were downloaded from the European Nucleotide Archive (ENA) of the European Bioinformatics Institute ENA-(EBI) using 'ENA File Downloader version 1.2' [47] and PEMA was run on the in-house HPC cluster.

All analyses were conducted on identical Dell M630 nodes (128GB RAM, 20 physical Intel Xeon 2.60GHz cores).

Mock community evaluation

PEMA was tested against mock communities and 3 statistical metrics were calculated to estimate the accuracy of its output. Thus, the precision, i.e the ratio of true positives (TP) over the total number of true (TP) and false positives (FP) predicted by a model (precision = TP / (TP + FP)), the recall, i.e the ratio of TP over the total number of TP and false negatives (FN) (recall = TP / (TP + FN)) and the F1-score, i.e the harmonic mean of precision and recall indices were estimated [48] to that end.

Table 1: Summary benchmark of PEMA marker-gene specific mock community recovery

(precision)

**marker gene**	**precision**	**recall**	**F1**
16S rRNA	0.81	0.85	0.83
18S rRNA	0.75	0.90	0.82
ITS	0.79	0.94	0.86
COI	0.62	0.93	0.74

Adequate accuracy was achieved when PEMA was used to recover the marker gene specific mock communities at the genus level. Precision and recall scores of ~80% or more are observed with two exceptions in precision but also three very high scores in recall. Overall the precision and recall harmonic mean (F1) scores range from 74% to 86%. A detailed description of the benchmark methodology and statistics analysis is given in the Additional file 2: Mock Communities.

Detailed presentation of per-marker-gene-specific mock community recovery via PEMA is provided in the following sections. A number of different sets of parameters was chosen for each marker gene. Each marker gene has special features (length variability, sequence variability etc.) and each Illumina run has its own intrinsic biases (primers used, PCR protocol etc.); thus, parameters' tuning plays a crucial part in metabarcoding analyses. In an attempt to analyze thoroughly the sequence data from the mock communities, various sets of parameters were tested based on the experimental details of the published studies but also in an exploratory way.

16S rRNA

When PEMA was performed with the Swarm v2 algorithm (d = 3, strictness = 0.6) and the singletons were not removed, 18 out of the 20 taxa were identified to the genus level; 3 of them even to the species level. There were 2 species, *Deinococcus radiodurans* and *Propionibacterium acnes*, that were not found in any of the PEMA runs. According to *Gohl* et al. [41], there was a discrepancy in the identification of those two species which was dependent on the amplification protocol used, i.e. EMP (Taq) or DI (KAPA) protocol. It is worth mentioning that as d increases, taxa cannot be identified to species level at all; however, there is a great shrinkage of the false positive assignments. Thus, when d = 30 and strictness = 0.6 for the KAPA

samples, *Enterococcus* is not identified at all, however PEMA gets its greatest F1 value (at the genus level, see Table 1), as the false positive assignments returned are minimized. When PEMA was run using the VSEARCH clustering algorithm, high precision values were returned in all cases (>0.79). However, the recall values were decreased when using Swarm v2 (0.65 - 0.68).

A great number of different parameter settings were tested, especially for the steps of quality trimming of the reads and the OTU clustering / ASV inference. The differences of their output indicate how sensitive this method is, as well as the great need of a mock community in every metabarcoding study; both as a control but also as a "tuning system" for the parameter setting of the pipeline used.

18S rRNA

When PEMA was performed using Swarm v2 algorithm (*d* = 1 , strictness = 0.5), 3 out of 12 community members were identified to species level (*Isochrysis galbana*, *Nannochloropsis oculata* and *Thalassiosira pseudonana*), 6 to genus and the rest 3 to class; the latter were all the green algae species (Chlorophyta) of the mock community. However, a better F1 score (0.82) was achieved when the class of Chlorophyceae was not found at all (*d* = 1, strictness = 0.3) as the false positives were decreased to only 1. When the VSEARCH algorithm was used, *Isochrysis galbana* was identified only to the genus level, the *Nannochloropsis* to the order level (Eustigmatales) and the *Poterioochromonas* genus to its class (Chrysophyceae). Furthermore, as *Bradley* et al. [42] support, the 2 marine haptophytes of the mock community (*Prymnesium* and *Isochrysis*) are significantly underrepresented in the samples of the V4 region.

ITS

Running PEMA by making use of the Swarm v2 algorithm (*d* = 20) and targeting the ITS2 region, ASVs from 5 of the 19 species of the mock community were assigned to species level, 10 to genus, 2 to family and 2 to class level. Contrary to Bakker's study [43], PEMA identified the genus *Chytriomyces* in all three samples, as well as the Ustilaginaceae family. Only one false positive assignment was recorded. When the CROP algorithm was used, PEMA's output was less accurate; the *Fusarium* species contained in the mock

community were not identified further than their family (Nectriaceae). As mentioned in Bakker's study [43], many reads deriving from the *Fusarium* spp. were not assigned to species level because of the quality trimming step. In addition, a manually assemble reference database for the taxonomy assignment was used in the initial study, containing only sequences of the mock community species, which biased this step and cannot be directly comparable to our case.

COI

Running PEMA on *Bista* et al. dataset [44] and using Swarm v2 (d = 10) identified 12 out of the 14 species included in the mock community. The sole non-identified species were *Bithynia leachii* and Anisus vortex. For *B. leachii* no entry exists in the MIDORI database, version MIDORI_LONGEST_1.1. However, the existence of another species of the genus *Bithynia* was recorded. With respect to *A. vortex*, PEMA returned a high abundance ASV assigned to the *Anisus* genus but with a low confidence level. PEMA managed to identify all the members of the mock community. This includes *Physa fontinalis*, originally not designed to be a member of the mock community but as *Bista* et al. [44] explain, was recorded due to cross-contamination. In the case of COI marker gene, unique sequences with low abundances (singletons or doubletons) often lead to spurious MOTUs/ASVs. Thus, as shown in the Additional file 2: Mock Communities, the false positives assignments are decreased when these low abundant sequences are removed.

In addition, as shown in Additional file 2: Mock Communities, the abundance of the assignments retrieved can indicate false positive assignments. Thus, true positive assignments occur with abundances of hundreds or even thousands of reads. Contrary to most of the false positives whose abundance is less than 10. That is mostly for the case of the COI marker gene, as Eukaryotes are under study; Eukaryotes have a great number of copies of this marker gene - different number of copies among the different species - and not just a single one as it is almost always the case in Bacteria. Therefore, assignments with such low abundances should be doubted as true positives in analyses on real datasets.

Comparison to existing software

By the means of evaluation, PEMA’s features were compared with those of mothur [4], QIIME 2 [5], LotuS [6] and Barque [7]. Table 2 presents a detailed comparison among the four tool features in terms of marker gene support, diversity and phylogeny analysis capability, parameter setting and mode of execution, operation system availability and HPC suitability. As shown, PEMA is equally feature-rich, if not richer in certain feature categories, to the other software packages. In particular, PEMA’s support for COI marker gene studies is distinctive; two methods for the taxonomy assignment are supported and PEMA’s easy-parameter setting, step-by-step execution and container distribution render it user and analysis friendly.

Table 2: Pipeline comparison.

**Feature**	**LotuS**	**QIIME 2**	**mothur**	**Barque**	**PEMA**
16S rRNA	☑	☑	☑	☐	☑
18S rRNA	☑	☑	☑	☐	☑
ITS	☑	☑	☐	☐	☑
COI	☐	☐	☐	☑	☑
diversity indices	☐	☑	☑	☐	☑
alignment-based taxonomy assignment	☑	☑	☑	☑	☑
phylogenetic-based taxonomy assignment	☑	☑	☐	☐	☑
parameters assigned in the command line	☑	☑	☑	☐	☐
parameters assigned through a text file	☑	☐	☐	☑	☑
step-by-step execution	☑	☑	☑	☐	☑
all steps in one go possible	☑	☐	☐	☑	☑
available for any Operating System (Linux, OSX, Windows)	☐	☑	☑	☐	☑

traditional application installation	☑	☑	☑	☑	☑
available as a virtual machine	☐	☑	☐	☐	☐
available as a container	☐	☑	☐	☐	☑
available for HPC as a container (Singularity container)	☐	☐	☐	☐	☑

Comparison of the basic features of the different pipelines.

Evaluation on real datasets and against other tools

16S rRNA marker gene analysis evaluation

To evaluate PEMA's performance, a comparative analysis of the *Pavloudi* et al. [45] dataset with mothur [4], QIIME 2 [5], LotuS [6] and PEMA was conducted.

It is known that the choice of parameters affects the output of each analysis; therefore, it is expected that different user choices might distort the derived outputs. For this reason and for a direct comparison of the pipelines, we have included all the commands and parameters chosen in the framework of this study in the Additional file 1: Supplementary Methods. The results of the processing of the sequences by PEMA are shown in Additional file 3: Table S1. All analyses were conducted on identical Dell M630 nodes (128GB RAM, 20 physical Intel Xeon 2.60GHz cores). LotuS, mothur and QIIME 2 operated in a single thread (core) fashion. PEMA, given the BDS intrinsic parallelization [10], operated with up to the maximum number of node cores (in this case 20).

The execution time and the reported OTU number of each tool are presented in Table 3. LotuS and PEMA resulted in a final number of OTUs comparable to that of *Pavloudi* et. al [45]. Clearly, due to PEMA's parallel-execution support, the analysis time can be significantly reduced (~1.5 hours in this case). The executional time is depending on the parameters chosen for each software (see Additional file 1: Supplementary Methods).

Table 3: OTU predictions and executional time for the different pipelines.

	LotuS	mothur	QIIME 2	PEMA	*Pavloudi* et al. [45]

			Deblur	DADA2		
Number of OTUs	9849	142669	517	1023	6028	7050
Executional time (h)	~9	~67 (~56 if the reference database is already built)	~2.5	~5	~1.5	~26

Due to the non-full overlap of the sequence reads, mothur resulted in an inflated number of OTUs; thus, is was excluded from further analyses. The results of all the pipelines were analyzed with the phyloseq script that is provided with PEMA. The taxonomic assignment of the PEMA retrieved OTUs is shown in Figure 5. The phyla that were found in the samples are similar to the ones that were found in the original study [45]. Although the lowest number of OTUs was found in the marine station (Kal) (Additional file 4: Table S3), which is not in accordance with *Pavloudi* et. al [45], the general trend of the decreasing number of OTUs with the increasing salinity was observed as it was in the original study (Additional file 5: Figure S1). Notably, this result was not observed with the other tested pipelines (Additional file 4: Table S3). Furthermore, each of the pipelines resulted in a different taxonomic profile (Additional files 6-8: Figure S2-4) with an extreme case of missing the Order of Betaproteobacteriales (Additional files 9-11: Figure S5-7).

Moreover, when the PERMANOVA analysis was run for the results of PEMA, LotuS and DADA2, it was clear that the microbial community composition was significantly different in each of the three sampled habitats (i.e. River, Lagoon, Sea) (PERMANOVA: F.Model = 7.0718, $p < 0.001$; F.Model = 6.5901, $p < 0.001$, F.Model = 2.2484, $p < 0.05$, respectively), which is in accordance with *Pavloudi* et. al [45]. However, this was not the case with Deblur (PERMANOVA: $p > 0.05$).

Overall, PEMA's output is in accordance with the original study [45]. PEMA performed equally well with

the other tested pipelines, if not better, in capturing the microbial community diversity and composition of the samples; simultaneously, it had the shortest executional time.

COI marker gene analysis evaluation

Bista et al. [46] created two COI libraries of different sizes: COIS (235 bp amplicon size) and COIF (658 bp amplicon size). The sequencing reads of COIS were selected for PEMA's evaluation; the COIF sequencing read pairs had no overlap so as to be merged and therefore were not considered appropriate for the analysis.

As previously, PEMA's performance was evaluated through a comparative analysis of the *Bista* et al. [46] dataset with Barque [7]; the commands and parameters chosen can be found in the Additional file 1: Supplementary Methods. Regarding the creation of the MOTU table, in the *Bista* et al. [46] study VSEARCH [16] was used with a clustering at 97% similarity threshold. Afterwards, the BLAST+ (megablast) algorithm [49] was used against a manually created database including all NCBI GenBank COI sequences of length >100 bp (June 2015) while excluding environmental sequences and higher taxonomic level information [46]. As discussed in the publication, this approach resulted in 138 unique MOTUs out of which 73 were assigned to species level. For PEMA's evaluation, the chosen clustering algorithm was Swarm v2, using different options for the cluster radius (*d*) parameter (Table 4); according to *Mahé* et al. [18], this is the most important parameter as it affects the number of MOTUs that are being created. The resulting MOTUs were classified against the MIDORI reference database [25] using RDPClassifier [24]. The results of the processing of the sequences are shown in Additional file 12: Table S3. For the case of Barque, the BOLD Database was used [50].

As shown in Table 4, PEMA resulted in 83 species level MOTUs with a cluster radius (*d*) of 2, which is very similar to that of the published study (i.e. 73 species). Although both the clustering algorithm and the taxonomy assignment methods were different between the original [46] and the present study, the results regarding the number of unique species present in the samples are in agreement to a considerable extent.

The computational time required by PEMA for the completion of the analysis is also shown in Table 4. Regardless of the value of the *d* parameter, all analyses were completed in about 2 hours, ie. adequately

fast to allow parameter testing and customization. Regarding Barque, the analysis resulted in the identification of 51 species level MOTUs and was concluded in 15 minutes. This difference is due to the error correction step of PEMA (BayesHammer algorithm [30]) which plays an important part in the enhanced results PEMA returns but it also requires a certain computational time; Barque does not have an analogous step, therefore its overall executional time is shorter.

Table 4: PEMA's output and executional time.

	***d* = 1**	***d* = 2**	***d* = 3**	***d* = 10**	***d* = 13**
MOTUs after preprocess and clustering steps	83791	59833	33227	7384	4829
MOTUs after chimera removal	80347	57863	32539	7339	4796
Non singletons MOTUs	6381	4947	2658	1914	1634
Assigned species	62	83	86	86	84
Executional time (h)	02:01:35	02:09:49	01:51:44	02:17:26	02:31:15

PEMA's output and executional time (using a 20 core node) for different values of Swarm's *d* parameter.

The taxonomic assignment of the retrieved MOTUs is shown in Figures 3-4. Certain .fastq files contained very few reads, such as those for sample ERR1308241, and therefore resulted in zero MOTUs upon the completion of PEMA; thus, these samples are not included in Figure 3. It is worth mentioning that PEMA performed better in identifying taxa that were included in the positive control contents of the published study than Barque (Table 5).

Table 5: Comparison of the taxonomy of retrieved MOTUs among PEMA, Barque and the positive controls of *Bista* et al. [46].

**Barque**	**PEMA**	***Bista* et al.** [46]
*Ablabesmyia monilis**	*Ablabesmyia monilis**	*Ablabesmyia monilis*

	*Crangonyx pseudogracilis**	*Crangonyx pseudogracilis*
	Radix sp.*	*Radix* sp.
	Chironomidae sp.*	Chironomidae sp.
	Ancylus sp.**	*Ancylus fluviatilis*
	Athripsodes aterrimus, *Athripsodes cinereus***	*Athripsodes albifrons*
*Chironomus anthracinus***	*Chironomus* sp., *Chironomus anthracinus*, *Chironomus pseudothummi*, *Chironomus riparius***	*Chironomus tentans*
*Polypedilum sordens***		*Polypedilum nubeculosum*
*Athripsodes aterrimus***		*Athripsodes albifrons*

*: Taxonomies identical to the published study (species level). **: Taxonomies identical to the published study (genus level).

OTU clustering *vs* ASV inference

There is an ongoing discussion about whether ASVs exceed OTUs. The strongest argument to this end, is that ASVs are real biological sequences. Hence, they can be compared between different studies in a straight-forward way; considered as consistent labels. In comparison, *de novo* OTUs are constructed, or "clustered", with respect to the emergent features of each specific dataset. Therefore, OTUs defined in two different data sets cannot be directly compared.

However, the OTU concept is not compulsory related to the clustering approach; it is widely used to describe results based on its biological meaning but it does not imply clustering. In addition, according to *Callahan* et al. [14] "ASV methods infer the biological sequences in the sample prior to the introduction of amplification and sequencing errors, and distinguish sequence variants differing by as little as one

nucleotide". As a result, ASVs could be considered as OTUs of higher resolution.

It is due to this concept confusion that algorithms whose rationele is considerably closer to the variant-based approach, are still considered as OTU clustering algorithms. Swarm v2 produces all possible "microvariants" of an amplicon to implement an exact-string comparison [18]. Furthermore, real biological sequences, "clouds of microvariants", are produced as its output, which can be used for comparisons between different studies. Thus, Swarm v2 can be considered as an ASV inferring algorithm. Traditional clustering methods have certain limitations such as arbitrary global clustering thresholds, centroid selection, as it depends on the input order, time-consuming etc, that variant-based approaches manage to address. However certain algorithms for OTU clustering as VSEARCH have been proven to be especially reliable and they are widely used by a great number of researchers. Furthermore, ASVs intend to improve taxonomic resolution; however, too much diversity often leads to a vast number of inferred ASVs. Thus, the statistical analyses get more complicated and the identification of sets of taxa whom abundances fluctuate across gradients or sample categories becomes even harder.

ESV or OTU approaches are supported by PEMA, though we support that similar ecological results are produced by both these methods, as also suggested by *Glassman* et al. [51].

Beyond environmental ecology, on-going and future work

PEMA is mainly intended to support eDNA metabarcoding analysis and be directly applicable to next-generation biodiversity/ecological assessment studies. Given that community composition analysis may also serve additional research fields, eg. microbial pathology, the potential impact of such pipelines is expected to be much higher. On-going PEMA work focuses on serving a wide scientific audience and on making it applicable to more types of studies. The easy set up and execution of PEMA, allows users to work closely with national and European HPC/e-infrastructures (e.g. ELIXIR Greece [52], LifeWatch ERIC [53], EMBRC ERIC [54]). To that end and in a mid-term perspective, a Common Workflow Language (CWL) version of PEMA will be explored. The aim of this effort is to reach out to a wider scientific audience and address both their ongoing as well as future analysis needs.

By supporting the analysis of the most commonly used marker genes for Bacteria and Archaea (16S

rRNA), Fungi (ITS) and Metazoa (COI/18S rRNA), a holistic biodiversity assessment approach is now possible through PEMA and eDNA metabarcoding; Though, in a mid term perspective, it is our intention to allow *ad hoc* and in-house databases to be used as reference for the taxonomy assignment.

Conclusions

PEMA is an accurate, execution friendly and fast pipeline for eDNA metabarcoding analysis. It provides a per-sample analysis output, different taxonomy assignment methods and graphics-based biodiversity/ecological analysis. This way, in addition to (M)OTU/ASV calling, it provides users with both an informative study overview and detailed result snapshots.

Thanks to a nominal number of installation and execution commands required for PEMA to be set and run, it is considered essentially user friendly. In addition, PEMA's strategic choice of a single parameter file, implementation programming language, and multiple container-type distribution, grant it with speed (running in parallel), on-demand partial pipeline enactment, and provision for HPC-system-based sharing. All the aforementioned features, render PEMA attractive for biodiversity/ecological assessment analyses. By supporting the analysis of the most commonly used marker genes for Prokaryotes (Bacteria and Archaea), as well as Eukaryotes (Fungi and Metazoa), PEMA allows assessment of biodiversity in different levels of biodiversity. Applications may mainly concern environmental ecology with possible extensions to fields like microbial pathology and gut microbiome, inline with modern research needs, from low volume to big data.

Availability of supporting source code and requirements

Project name: PEMA

Project home page: https://github.com/hariszaf/pema

Archived version: see project home page (github repository)

Operating system(s): Platform independent

Programming language: BigDataScript

Other requirements: Singularity (in case of HPC usage)

License: GNU GPLv3 (for 3rd party components separate licenses apply)

Any restrictions to use by non-academics: licence needed

Availability of supporting data

The sequence data that support the findings of this study, with respect to the mock-community-based evaluation, are available in European Nucleotide Archive (ENA) with the following study accession numbers - for the 16S, 18S rRNA, ITS and COI marker genes respectively:

PRJNA305443 (https://www.ebi.ac.uk/ena/browser/view/PRJNA305443),

PRJNA314977 (https://www.ebi.ac.uk/ena/browser/view/PRJNA314977),

PRJNA377530 (https://www.ebi.ac.uk/ena/browser/view/PRJNA377530) and

PRJEB23036 (https://www.ebi.ac.uk/ena/browser/view/PRJEB23036)

The real datasets used are also available in ENA:

PRJEB20211 (http://www.ebi.ac.uk/ena/data/view/PRJEB20211) and

PRJEB13009 (https://www.ebi.ac.uk/ena/data/view/PRJEB13009).

Declarations

List of abbreviations

BDS: BigDataScript

COI: Cytochrome Oxidase Subunit 1

eDNA: Environmental DNA

MOTU: Molecular Operational Taxonomic Unit (used for Eukaryotes)

HPC: High Performance Computing

MCMC: Markov chain Monte Carlo

MSA: Multiple Sequence Alignment

OTU: Operational Taxonomic Unit (used for prokaryotes)

PEMA: a Pipeline for Environmental DNA Metabarcoding Analysis

SSU: Small Subunit

CWL: Common Workflow Language

Ethics approval and consent to participate

Not applicable

Consent for publication

Not applicable

Competing interests

The authors declare that they have no competing interests

Funding

This project has received funding from the Hellenic Foundation for Research and Innovation (HFRI) and the General Secretariat for Research and Technology (GSRT), under grant agreement No 241 (PREGO project). There was no additional external funding received for this study. The funders had no role in study design, data collection and analysis, decision to publish, or preparation of the manuscript.

Authors' contributions

HZ conceived and designed the pipeline, performed its containerization, analyzed and interpreted the data, wrote the paper, prepared figures and/or tables, reviewed drafts of the paper. HQV offered support in the HPC preparation and setup and in 3rd party component usage. KV and CA conceived the idea and reviewed drafts of the paper. PT conceived the idea, proposed the usage of the programming language and reviewed drafts of the paper. CP conceived the idea, prepared figures and/or table and reviewed drafts of the paper. AP offered support in HPC and in 3rd party components. EP conceived the idea, assisted with programming and setup and reviewed drafts of the paper. All authors read and approved the final manuscript.

Acknowledgements

The authors would like to thank: a. the Information technology (IT) group of HCMR and especially Mr Stelios Ninidakis, Mr Georgios Tsamis and Mr Dimitris Sidirokastritis for their help and support during cluster maintenance and installation of third party software. b. Dr. Christos A. Christakis (ORCID iD: 0000-0002-7075-0996) for his valuable feedback on ecological analysis usefulness aspects.

This research was supported in part through computational resources provided by IMBBC (Institute of Marine Biology, Biotechnology and Aquaculture) of the HCMR (Hellenic Centre for Marine Research). Funding for establishing the IMBBC HPC has been received by the MARBIGEN (EU Regpot) project, LifeWatchGreece RI and the CMBR (Centre for the study and sustainable exploitation of Marine Biological Resources) RI.

References

[1] Pavan-Kumar A, Gireesh-Babu P, Lakra WS. DNA metabarcoding: a new approach for rapid biodiversity assessment. J Cell Sci Mol Biol. 2015;2(1):111.

[2] Thomsen PF and Willerslev E. Environmental dna–an emerging tool in conservation for monitoring past and present biodiversity. Biological Conservation. 2015; 183:.4–18.

[3] Ji Y, Ashton L, Pedley SM, Edwards DP, Tang Y, Nakamura A, Kitching R, Dolman PM, Woodcock P, Edwards FA, Larsen TH. Reliable, verifiable and efficient monitoring of biodiversity via metabarcoding. Ecology letters. 2013 Oct;16(10):1245-57.

[4] Schloss PD, Westcott SL, Ryabin T, Hall JR, Hartmann M, Hollister EB, et al. Introducing mothur: open-source, platform-independent, community-supported software for describing and comparing microbial communities. Appl. Environ. Microbiol. 2009; 75:7537-41.

[5] Bolyen E, Rideout JR, Dillon MR, Bokulich NA, Abnet C, Al-Ghalith GA, et al. QIIME 2: Reproducible, interactive, scalable, and extensible microbiome data science. PeerJ Preprints. 2018; 6:e27295v2.

[6] Hildebrand F, Tadeo R, Voigt AY, Bork P, Raes J. LotuS: an efficient and user-friendly OTU

processing pipeline. Microbiome. 2014; 2:30.

[7] Normandeau E. Environmental DNA metabarcoding analysis. https://github.com/enormandeau/barque. Accessed 10 November 2019.

[8] Axtner J, Crampton-Platt A, Hoerig LA, Mohamed A, Xu CC, Yu DW, Wilting A. An efficient and robust laboratory workflow and tetrapod database for larger scale environmental DNA studies. GigaScience. 2019 Apr 13;8(4):giz029.

[9] European Strategy Forum on Research Infrastructures Innovation Working Group. Innovation-oriented cooperation of Research Infrastructures. Vol.3. ESFRI Scripta. 2018.

[10] Cingolani P, Sladek R, Blanchette M. BigDataScript: a scripting language for data pipelines. Bioinformatics. 2014; 31:10-16.

[11] Rad BB, Bhatti HJ, Ahmadi M. An introduction to docker and analysis of its performance. International Journal of Computer Science and Network Security (IJCSNS). 2017; 17:228.

[12] Kurtzer GM, Sochat V, Bauer MW. Singularity: Scientific containers for mobility of compute. PloS one. 2017; 12:e0177459.

[13] Coissac E, Riaz T, Puillandre N. Bioinformatic challenges for DNA metabarcoding of plants and animals. Molecular ecology. 2012 Apr;21(8):1834-47.

[14] Callahan BJ, McMurdie PJ, Holmes SP. Exact sequence variants should replace operational taxonomic units in marker-gene data analysis. The ISME journal. 2017 Dec;11(12):2639.

[15] Pauvert C, Buée M, Laval V, Edel-Hermann V, Fauchery L, Gautier A, Lesur I, Vallance J, Vacher C. Bioinformatics matters: the accuracy of plant and soil fungal community data is highly dependent on the metabarcoding pipeline. Fungal Ecology. 2019 Oct 1;41:23-33.

[16] Rognes T, Flouri T, Nichols B, Quince C, Mahé F. VSEARCH: a versatile open source tool for metagenomics. PeerJ. 2016; 4:e2584.

[17] Hao X, Jiang R, Chen T. Clustering 16S rRNA for OTU prediction: a method of unsupervised Bayesian clustering. Bioinformatics. 2011; 27:611-8.

[18] Mahé F, Rognes T, Quince C, de Vargas C, Dunthorn M. Swarm v2: highly-scalable and high-resolution amplicon clustering. PeerJ. 2015; 3:e1420.

[19] Lanzén A, Jørgensen SL, Huson DH, Gorfer M, Grindhaug SH, Jonassen I, et al. CREST–classification resources for environmental sequence tags. PloS one. 2012; 7:e49334.

[20] Quast C, Pruesse E, Yilmaz P, Gerken J, Schweer T, Yarza P, Peplies J, Glöckner FO. The SILVA ribosomal RNA gene database project: improved data processing and web-based tools. Nucl. Acids Res. 2013; 41:D590-6.

[21] Nilsson RH, Larsson KH, Taylor AF, Bengtsson-Palme J, Jeppesen TS, Schigel D, Kennedy P, Picard K, Glöckner FO, Tedersoo L, Saar I. The UNITE database for molecular identification of fungi: handling dark taxa and parallel taxonomic classifications. Nucleic acids research. 2018 Oct 29;47(D1):D259-64.

[22] Kozlov AM, Darriba D, Flouri T, Morel B, Stamatakis A. RAxML-NG: a fast, scalable and user-friendly tool for maximum likelihood phylogenetic inference. Bioinformatics. 2019; btz305.

[23] Barbera P, Kozlov AM, Czech L, Morel B, Darriba D, Flouri T, Stamatakis A. EPA-ng: massively parallel evolutionary placement of genetic sequences. Systematic biology. 2018; 68:365-9.

[24] Wang Q, Garrity GM, Tiedje JM, Cole JR. Naive Bayesian classifier for rapid assignment of rRNA sequences into the new bacterial taxonomy. Appl. Environ. Microbiol. 2007; 73:5261-7.

[25] Machida RJ, Leray M, Ho SL, Knowlton N. Metazoan mitochondrial gene sequence reference datasets for taxonomic assignment of environmental samples. Scientific data. 2017; 4:170027.

[26] McMurdie JP, Holmes S. phyloseq: an R package for reproducible interactive analysis and graphics of microbiome census data. PloS one. 2013; 8:e61217.

[27] Andrews S. FastQC. http://www.bioinformatics.babraham.ac.uk/projects/fastqc/. Accessed 08 July 2019.

[28] Bolger AM, Lohse M, Usadel B. Trimmomatic: a flexible trimmer for illumina sequence data. Bioinformatics. 2014; 30:2114-20.

[29] Martin M. Cutadapt removes adapter sequences from high-throughput sequencing reads. EMBnet. journal. 2011 May 2;17(1):10-2.

[30] Nikolenko SI, Korobeynikov AI, Alekseyev MA. Bayeshammer: Bayesian clustering for error correction in single-cell sequencing. BMC genomics. 2013; S7.

[31] Bankevich A, Nurk S, Antipov D, Gurevich AA, Dvorkin M, Kulikov AS, et al. Spades: a new

genome assembly algorithm and its applications to single-cell sequencing. Journal of computational biology. 2012; 19:455-77.

[32] Masella AP, Bartram AK, Truszkowski JM, Brown DG, Neufeld JD. PANDAseq: paired-end assembler for illumina sequences. BMC bioinformatics. 2012; 13:31.

[33] Boyer F, Mercier C, Bonin A, Le Bras Y, Taberlet P, Coissac E. OBITools: a unix-inspired software package for dna metabarcoding. Molecular ecology resources. 2016; 16:176-82.

[34] Edgar RC. Search and clustering orders of magnitude faster than BLAST. Bioinformatics. 2010 Aug 12;26(19):2460-1.

[35] Benson DA, Cavanaugh M, Clark K, Karsch-Mizrachi I, Ostell J, Pruitt KD, Sayers EW.GenBank. Nucleic acids research 2018; 46:D41-47.

[36] Czech L, Barbera P, Stamatakis A. Methods for automatic reference trees and multilevel phylogenetic placement. Bioinformatics. 2018; 35:1151-8.

[37] Berger SA, Stamatakis A. PaPaRa 2.0: a vectorized algorithm for probabilistic phylogeny-aware alignment extension. Heidelberg Institute for Theoretical Studies. 2012.

[38] Letunic I, Bork P. Interactive tree of life (itol): an online tool for phylogenetic tree display and annotation. Bioinformatics. 2006; 23:127-8.

[39] Katoh K, Misawa K, Kuma KI, Miyata T. Mafft: a novel method for rapid multiple sequence alignment based on fast fourier transform. Nucleic acids research. 2002; 30:3059-66.

[40] Chavez J. Singularity: a "Docker" for HPC environments. https://dev.to/grokcode/singularity--a-docker-for-hpc-environments-i6p. Accessed 08 Jul 2019.

[41] Gohl DM, Vangay P, Garbe J, MacLean A, Hauge A, Becker A, Gould TJ, Clayton JB, Johnson TJ, Hunter R, Knights D. Systematic improvement of amplicon marker gene methods for increased accuracy in microbiome studies. Nature biotechnology. 2016 Sep;34(9):942.

[42] Bradley IM, Pinto AJ, Guest JS. Design and evaluation of Illumina MiSeq-compatible, 18S rRNA gene-specific primers for improved characterization of mixed phototrophic communities. Appl. Environ. Microbiol.. 2016 Oct 1;82(19):5878-91.

[43] Bakker MG. A fungal mock community control for amplicon sequencing experiments. Molecular

ecology resources. 2018 May;18(3):541-56.

[44] Bista I, Carvalho GR, Tang M, Walsh K, Zhou X, Hajibabaei M, Shokralla S, Seymour M, Bradley D, Liu S, Christmas M. Performance of amplicon and shotgun sequencing for accurate biomass estimation in invertebrate community samples. Molecular ecology resources. 2018 Sep;18(5):1020-34.

[45] Pavloudi C, Kristoffersen JB, Oulas A, De Troch M, Arvanitidis C. Sediment microbial taxonomic and functional diversity in a natural salinity gradient challenge Remane's "species minimum" concept. PeerJ. 2017; 5:e3687.

[46] Bista I, Carvalho GR, Walsh K, Seymour M, Hajibabaei M, Lallias D, et al. Annual time-series analysis of aqueous edna reveals ecologically relevant dynamics of lake ecosystem biodiversity. Nature communications. 2017; 8:14087.

[47] Harrison PW, Alako B, Amid C, Cerdeño-Tárraga A, Cleland I, Holt S, et al. The European Nucleotide Archive in 2018. Nucleic acids research. 2018; 47:D84-8.

[48] Ting K.M. Precision and Recall. In: Sammut C., Webb G.I. (eds) Encyclopedia of Machine Learning. Springer, Boston, MA. 2011.

[49] Camacho C, Coulouris G, Avagyan V, Ma N, Papadopoulos J, Bealer K, Madden TL BLAST+: architecture and applications. BMC bioinformatics. 2009; 10:421.

[50] Ratnasingham S, Hebert PD. BOLD: The Barcode of Life Data System (http://www. barcodinglife. org). Molecular ecology notes. 2007 May;7(3):355-64.

[51] Glassman SI, Martiny JB. Broadscale ecological patterns are robust to use of exact sequence variants versus operational taxonomic units. MSphere. 2018 Aug 29;3(4):e00148-18.

[52] ELIXIR-GR. https://www.elixir-greece.org/ Accessed 08 July 2019.

[53] LifeWatch-ERIC. https://www.lifewatch.eu/ Accessed 08 July 2019.

[54] EMBRC. http://www.embrc.eu/ Accessed 08 July 2019.

Figure legends

Figure 1: PEMA comprises four parts. The first step (top left) is the quality control and pre-processing of the Illumina sequencing reads. This step is common for both 16S rRNA and COI marker genes. The second step (top right) is the clustering of reads to (M)OTUs or their inferring to ASVs. The third step (bottom left) is the taxonomy assignment to the generated (M)OTUs/ASVs. In the fourth step (bottom right), the results of the metabarcoding analysis are provided to the user and visualized.

Figure 2: Phylogeny-based taxonomy assignment. A: Building a reference tree for the phylogeny-based taxonomy assignment to 16S rRNA marker gene OTUs: from the latest edition of Silva SSU, all entries referring to Bacteria and Archaea were used and using "art" algorithm, 10000 consensus taxa were kept. B: Using PaPaRa and the OTUs that come up from every analysis, an MSA was made and EPA-ng took over the phylogeny based taxonomy assignment.

Figure 3: ASVs bar plot at the lowest possible taxonomic level for the *Bista* et al. dataset [46]. Bar plot depicting the taxonomy of the retrieved ASVs with confidence estimate equal or higher than 0.97 at the lowest possible taxonomic level.

Figure 4: ASVs bar plot at the species level for the *Bista* et al. dataset [46]. Bar plot depicting the taxonomy of the retrieved ASVs with confidence estimate equal or higher than 0.97 at the species level.

Figure 5: OTUs bar plot at the Phylum level. Bar plot depicting the taxonomy of the retrieved OTUs from PEMA for *Pavloudi*'s et al. [45] dataset, at the Phylum level for the case of the 16S marker gene.

Additional files

Additional file 1: Supplementary Methods: Description of tools invoked by PEMA and their licences. Description of the commands, along with their parameters, used to run PEMA, mothur, LotuS and QIIME 2.

Additional file 2: Mock Communities: Details about the mock communities chosen and their corresponding studies as well as the returned output of PEMA for each of those for a number of sets of parameters.

Additional file 3: Table S1: Number of sequences after each pre-processing step for the case of 16S rRNA gene.

Additional file 4: Table S2: Diversity indices of the samples.

Additional file 5: Figure S1: Linear regression between the number of OTUs (averaged per sampling station) and the salinity of the sampling stations. L: Lagoon. S: Sea. R: River. AR: Arachthos. ARO: Arachthos Neochori. ARDelta: Arachthos Delta. LOin: Logarou station inside the lagoon. LOout: Logarou station in the channel connecting the lagoon to the gulf. Kal: Kalamitsi.

Additional file 6: Figure S2: Bar plot depicting the taxonomy of the retrieved OTUs from LotuS at the Phylum level.

Additional file 7: Figure S3: Bar plot depicting the taxonomy of the retrieved OTUs from QIIME 2 using Deblur at the Phylum level.

Additional file 8: Figure S4: Bar plot depicting the taxonomy of the retrieved OTUs from QIIME 2 using DADA2 at the Phylum level.

Additional file 9: Figure S5: Bar plot depicting the taxonomy of the retrieved OTUs from LotuS at the class of Betaproteobacteriales.

Additional file 10: Figure S6: Bar plot depicting the taxonomy of the retrieved OTUs from QIIME 2 using Deblur at the class of Betaproteobacteriales.

Additional file 11: Figure S7: Bar plot depicting the taxonomy of the retrieved OTUs from PEMA at the class of Betaproteobacteriales.

Additional file 12: Table S3: Number of sequences after each pre-processing step for the case of COI,

dataset from *Bista* et al. [46].

Figure 1

Click here to download Figure Figure 1.png

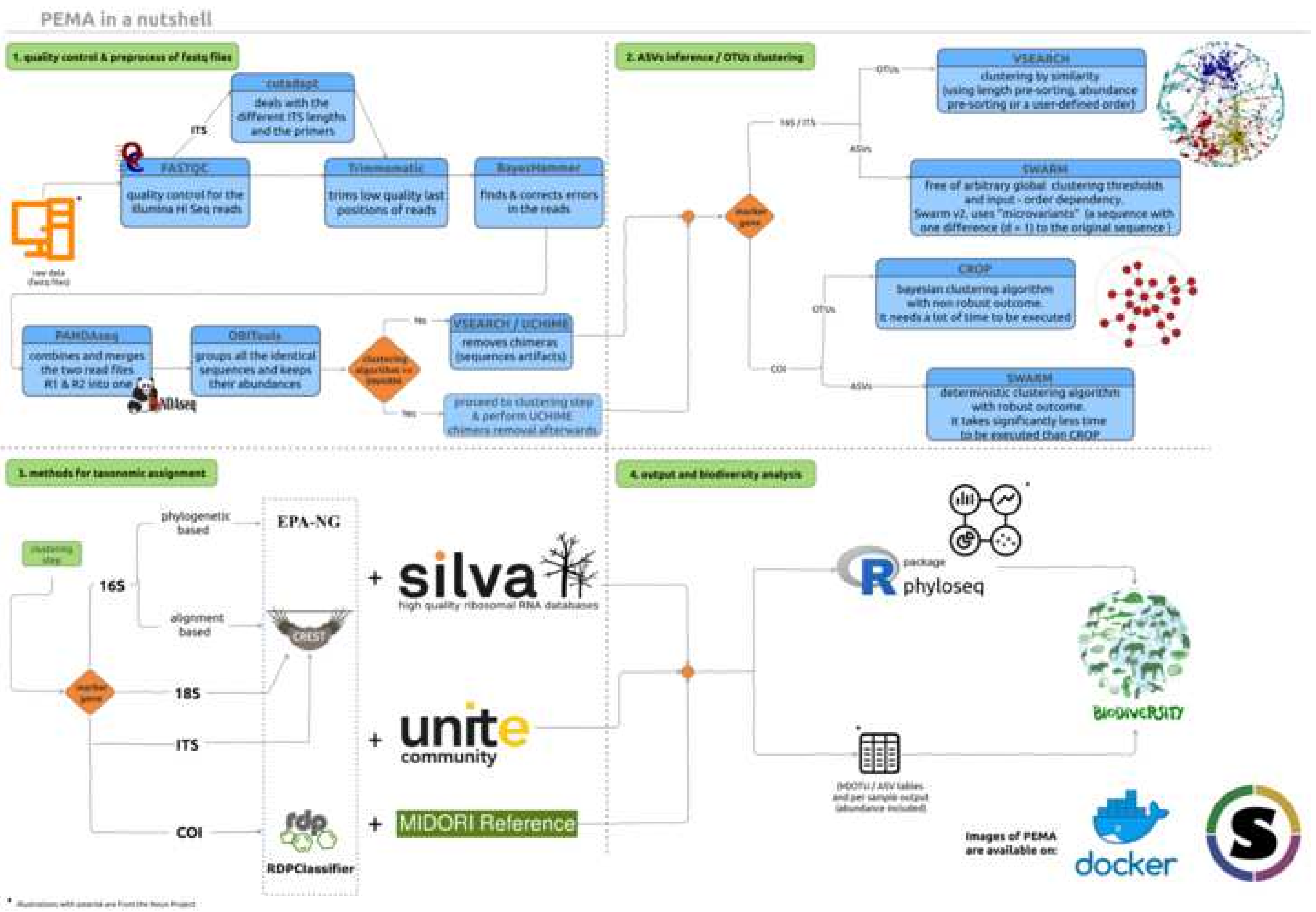

Figure 2 Click here to download Figure Figure 2.png

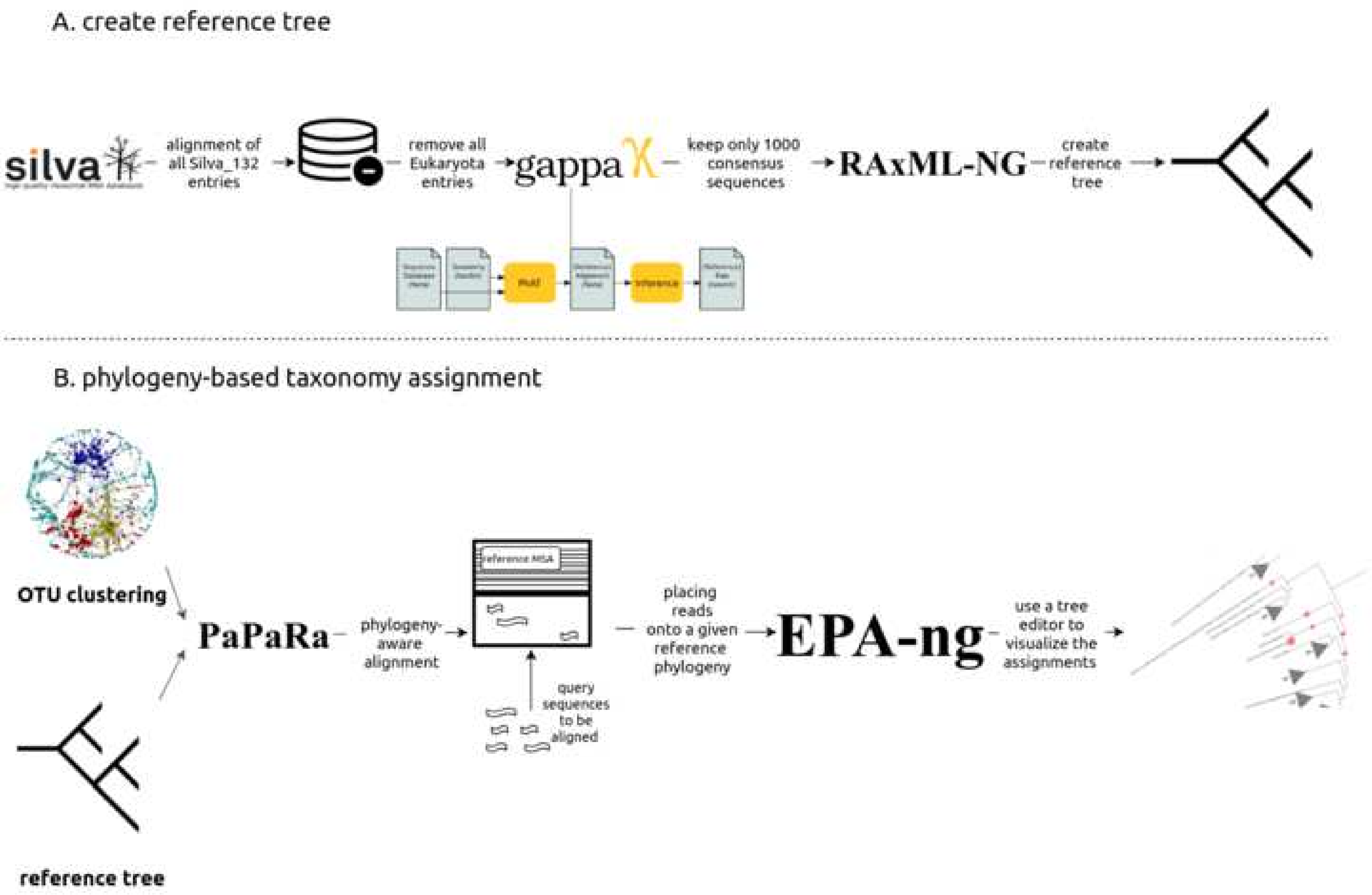

Figure 3

Click here to download Figure Figure 3.png

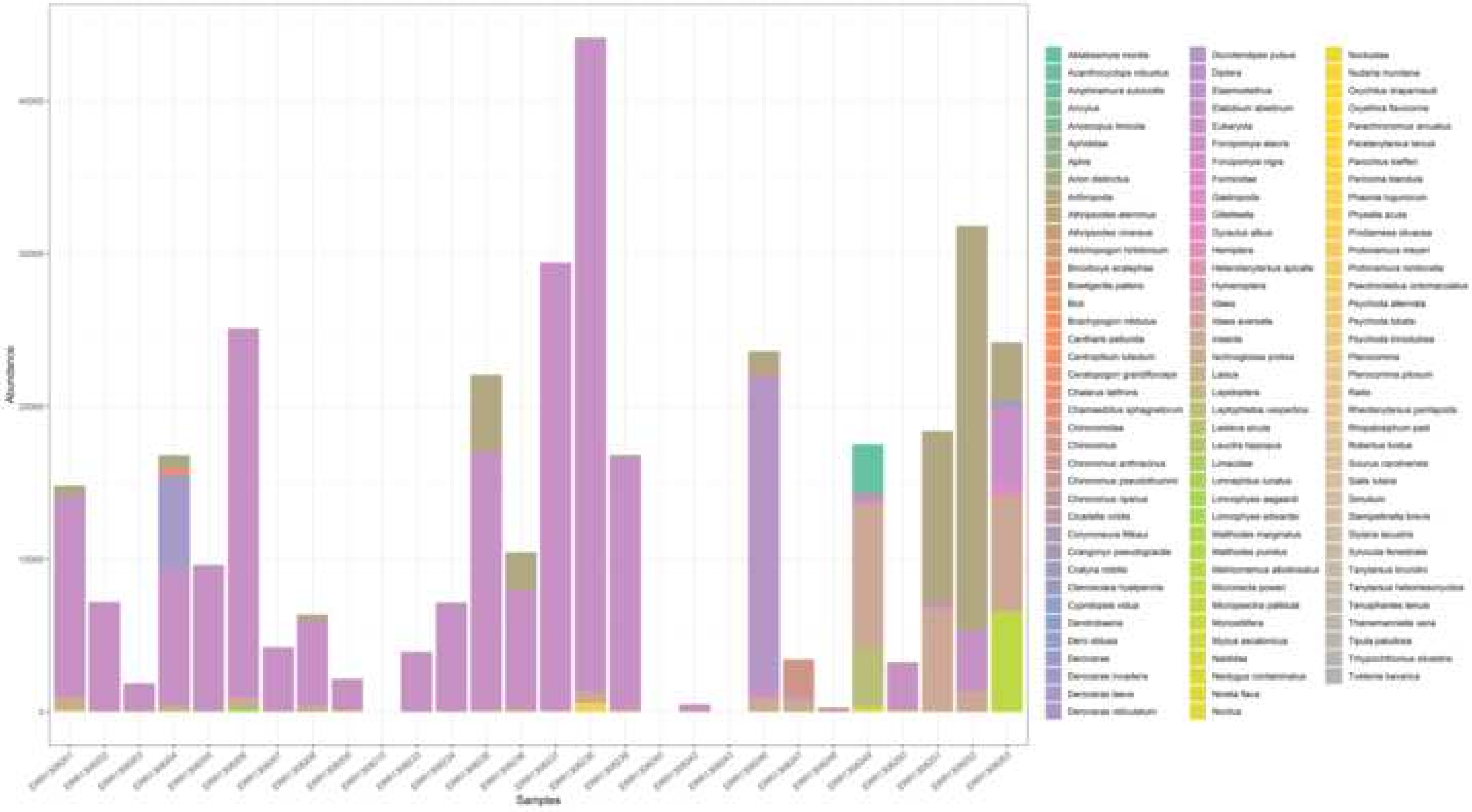

Figure 4

Click here to download Figure Figure 4.png

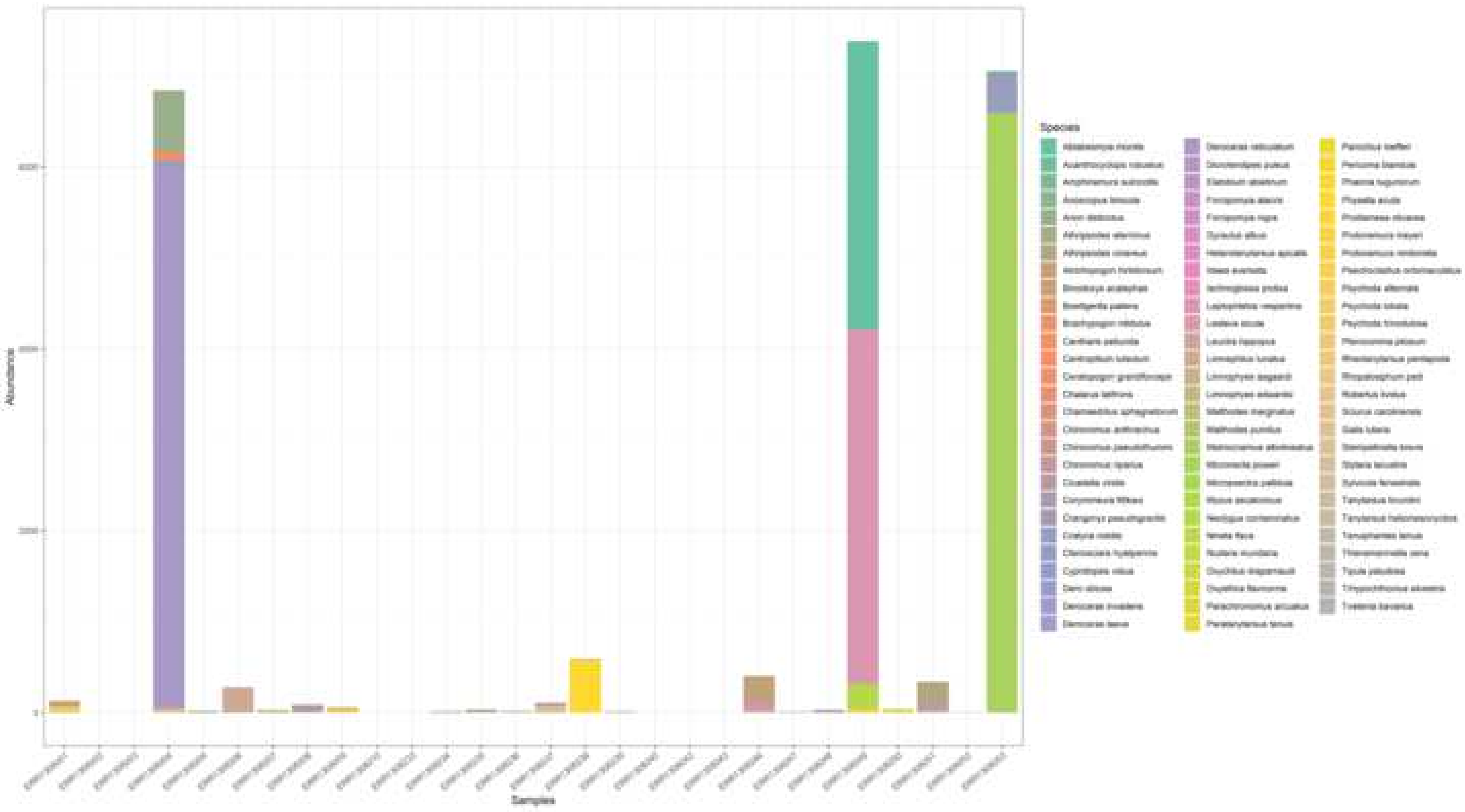

Figure 5 Click here to download Figure Figure 5.png

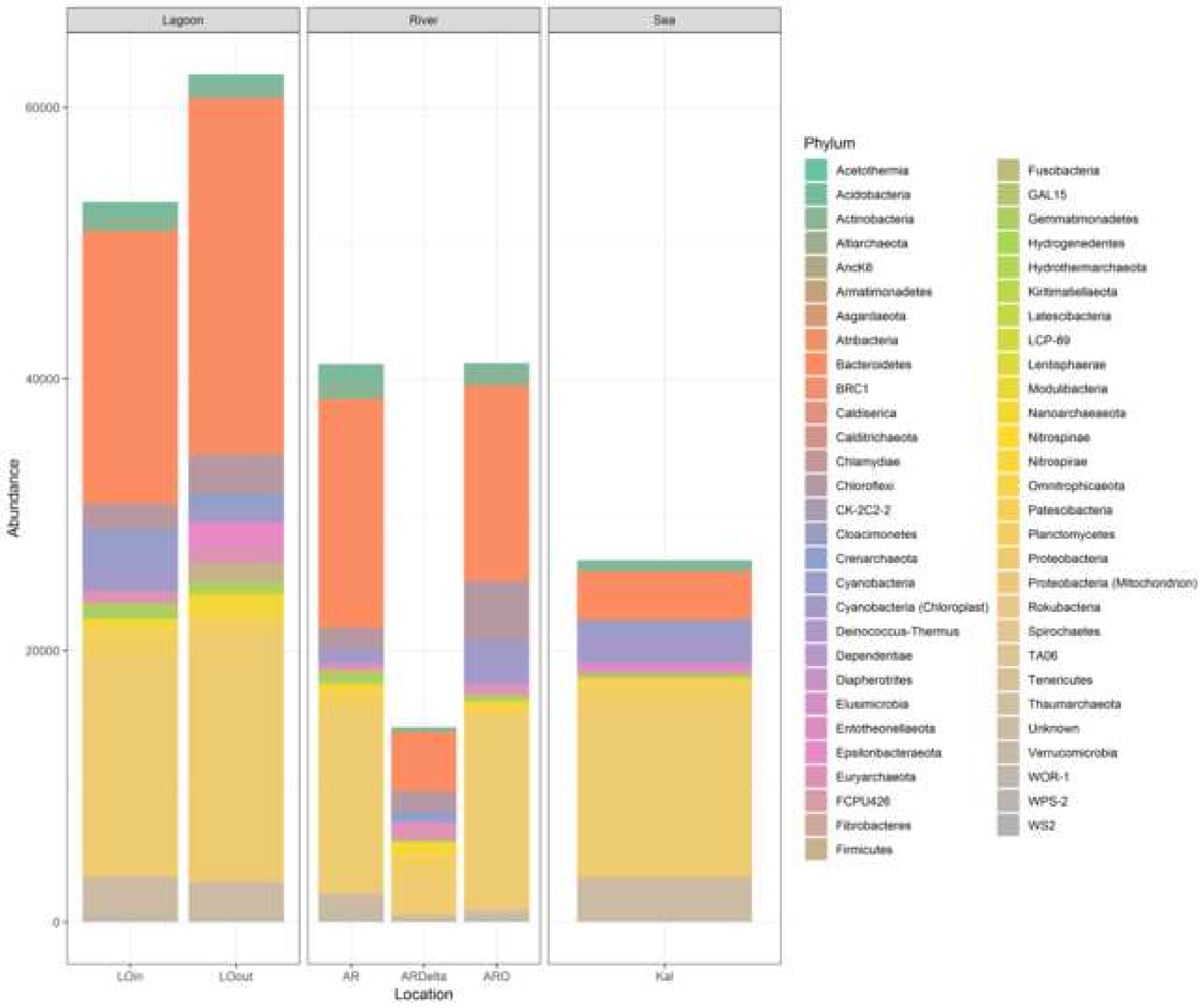

Additional file 1

Click here to access/download
Supplementary Material
Additional file 1_ Supplementary Methods.docx

Additional file 2

Click here to access/download
Supplementary Material
Additional file 2_ Mock Communities_.xlsx

Click here to access/download
Supplementary Material
Additional file 3 Table S1.docx

Additional file 4

Click here to access/download
Supplementary Material
Additional file 4 Table S2.docx

Additional file 5

Click here to access/download
Supplementary Material
Additional file 5_ Figure S1.png

Additional file 6

Click here to access/download
Supplementary Material
Additional file 6 - Figure S2.png

Additional file 7

Click here to access/download
Supplementary Material
Additional file 7 - Figure S3.png

Additional file 8

Click here to access/download

Supplementary Material

Additional file 8 - Figure S4.png

Additional file 9

Click here to access/download
Supplementary Material
Additional file 9 - Figure S5.png

Additional file 10

Click here to access/download
Supplementary Material
Additional file 10 - Figure S6.png

Additional file 11

Click here to access/download
Supplementary Material
Additional file 11 - Figure S7.png

Additional file 12

Click here to access/download
Supplementary Material
Additional file 12_ Table S3.docx

Response to the editor's and reviewers' comments

We would like to kindly thank the reviewers for the time they spent to thoroughly read our manuscript. We sincerely appreciate all accurate comments made by the reviewers, which helped the improvement of our manuscript. In the revised version, we have addressed all of the reviewers' comments and suggestions and where necessary we have incorporated changes and made amendments and alterations to the manuscript. The changes and the new sections of the manuscript are written in blue. Below we cite our detailed answers (in blue) to the editor and reviewers' comments and suggestions (italic).

Editor's comment #1

Both reviewers mention flexibility and performance as positive aspects of your tool, and I feel that, with some major extensions and improvements, a revised version may be suitable for publication in GigaScience.

With respect to Editor's main prompt for major extensions and improvements, we resubmit the PEMA manuscript including two more marker genes (18s rRNA and ITS) and allowing not only OTU clustering, but inferring ASVs as well. In addition, PEMA was evaluated for all the four marker genes not only against results of already published studies, but against mock communities as well. Finally, major improvements in the manuscript have been incorporated.

Editor's comment #2

However, both reviewers also mention that there are many available tools that perform similar tasks, and it is not clear at the moment whether the tool really presents a major advance in the field.

PEMA's novelty is detected on three major features. It supports the analysis of all marker genes used for environmental studies (16S rRNA for Bacteria and Archaea, ITS for Fungi and COI/18S rRNA for Metazoa). It can be used for the case of big datasets; that is because of the BigDataScript programming language in which PEMA has been implemented. In addition, PEMA has been developed in a High Performance Computing (HPC) - based approach to be able to support such analyses. Furthermore, container-based technologies as Docker, for the case of personal computers, and Singularity, for the case of HPC environments, allow PEMA to be rather easy to install. At the same time, the plain *parameter-value* pair text file in which the user sets all the parameters required, makes PEMA user friendly. Finally, the *checkpoints* created allow for further partial re-running of the pipeline, providing the possibility of "investigating" the best tuning for every analysis. It is our belief that the aforementioned features contribute in addressing major issues in the metabarcoding approach.

Editor's comment #3

PEMA currently only allows COI marker sequences. Extending the functionality for other markers (and showing respective validation data) could be a major improvement.

As already mentioned, PEMA has been extended and the 18S rRNA and ITS marker genes are now supported. Further evaluation of PEMA's findings has been performed by running PEMA on mock communities.

Editor's comment #4

Both reviewers mention that Amplicon Sequence Variants are increasingly being used instead of OTUs. I feel if your tool could support ASVs, it would be easier to convince us that the tool is an advance for state-of-the-art applications.

The Swarm v2 algorithm is now an option for all the marker genes supported by PEMA. We support that Swarm v2 produces ASVs as its output meet all ASVs' inferring features; that is also the belief of both Swarm's and DADA2's authors. In addition, with respect to the ongoing discussion about replacing OTU clustering with ASV inferring, an extensive discussion has been added in the manuscript.

Editor's comment #5

Both reviewers ask for a more detailed interpretation of the results in comparison with previous metabarcoding pipelines.

Mock communities were used to address this issue. For all four marker genes comparisons with other pipelines that support each of those was performed. In addition, the comparisons presented in our first submission are now further detailed.

Reviewer's #1 comment #1

I have reviewed the manuscript from Zafeiropoulos et al. "PEMA: from the raw .fastq files of 16S rRNA and COI marker genes to the (M)OTU-table, a thorough metabarcoding analysis". Authors present a flexible pipeline, based on existing bioinformatic tools, to analyse 16S and COI metabarcoding data. This pipeline does not contain any improvement over existing algorithms or analytic tools; but its flexibility and performance time are undoubtedly its major advantages, allowing the user to choose between different tools at each step (and to easily switch from one to another). However, I am not convinced this note meets the novelty and quality standards required for publication in Gigascience.

I am not questioning here the potential usefulness of PEMA but similar (and somehow more flexible) friendly-user pipelines have recently been developed (e.g. SLIM, Dufresne et al. 2019 BMC Bioinformatics). One of the motivation of authors seemed originally to be the adaptation of existing pipelines to markers other than bacterial 16S (line 76) but, although this cannot be considered as a critical flaw, PEMA currently allows only COI sequences to be processed. I understand that PEMA could be extended to support other markers by the use of curated reference databases for other taxonomic groups (e.g., SILVA or PR2 for nuclear ribosomal markers, UNITE for fungal ITS etc.). Nevertheless, even if authors claim that ITS and 18S rRNA marker genes will be supported at medium-term, the lack of such capability reduces the interest of this pipeline compared to existing ones (e.g. SLIM).

With respect to PEMA's novelties and quality standards required for its publication in Gigascience, please see Response to Editor's comment #2. As already mentioned, both 18S rRNA and ITS marker genes are now supported by PEMA.

Regarding the Slim tool, it is our belief that although PEMA and Slim share a number of common features, they should not be directly compared. This is due to their current implementations, that are optimised for different analysis enactment environments. Slim provides an equally good output (from a biological analysis perspective) to non-specialist or command-line reluctant researchers. This is why it has been implemented via a web-based user interface. On the back-end of a Slim installation a local or cloud-based computing server is employed; not however an HPC system. Docker, which is provided as an easy way to run Slim on your own personal computer and is used to ease the back-end (server and sequences analysis tools) components, is also HPC-deficient, e.g. in comparison to Singularity [1]. Contrary, PEMA is an HPC-based tool designed for datasets bigger than what a laptop (personal computer) can address. PEMA draws its simplicity from providing researchers with an easy to set-run (and tweak if needed) environment asking for merely basic command line knowledge. PEMA takes advantage of a series of state-of-the-art HPC-dedicated technologies like the Singularity-based containerisation and BigDataScrip workflow enactment). In order to compare PEMA with other metabarcoding pipelines, LotuS, QIIME2 (includes DADA2 as well) and mothur were performed for the case of the 16S rRNA and Barque for the COI marker gene.

Reviewer's #1 comment #2

Second, the main text lacks information about the choice of the algorithms and tools introduced in PEMA. For instance, why choosing to include Swarm and CROP for mOTU clustering (or RDPClassifier and LCAClassifier for taxonomic assignment) over other tools? Note that more and more studies now rely on ASV (Amplicon Sequence Variant) rather than OTU clustering. It would have been interesting to discuss the choice of including only OTU clustering programs.

Advantages and features of each of the tools invoked by PEMA are extensively described in the Supplementary Methods file. That is to avoid adding technical details in the main manuscript. With respect to the OTU clustering - ASV inferring issue, please see Response to Editor's comment #4.

Reviewer's #1 comment #3

Finally, the abstract claims that "PEMA was evaluated against previously published datasets and achieved

comparable quality results". However, I do not really understand the way the comparisons are made (apart from comparing the execution time). The fact that a similar number of OTUs or identified species is retrieved does not provide information about the PEMA performance but about the variability generated when the user decides to use one tool or another. Indeed, alpha-diversity patterns are highly sensitive to the tools used in the data processing and their parameters (beta-diversity patterns are probably more relevant in this regard). Similarly, the fact that authors retrieve alpha-diversity patterns originally published only with PEMA when comparing the outputs of the 16S dataset
analysed with different pipelines (lines 247-254) does not mean that PEMA performs better, but rather that the tools used in PEMA were more analogous to the original ones in the way they processed the data. And it is the case: Unlike PEMA and the original pipeline used to process the dataset (Pavloudi et al. 2017), Deblur and DADA2 do not use clustering but rather aim to identify putative true sequences (and are thus much more conservative), which explain the variation in alpha-diversity between pipelines.
Metabarcoding is a method commonly used for exploratory studies. Thus, it is important to assess the variability that different tools introduce in the produced outputs of each study. The aforementioned variability can distort the results of a study and skew the conclusions that are subsequently formulated. Undoubtedly, the execution time is a crucial issue when choosing tools for analysis of metabarcoding datasets. However, the results produced should be also compared. In our case, this comparison led to the conclusion that certain tools/algorithms, such as DADA2, could not have identified taxonomic groups that were present in the dataset (as mentioned in the original publication) which would have caused a major distortion in the conclusions of the study. We believe that, under this prism, it is important to show that PEMA outperformed the tools that it was compared against.
In addition, please see Response to Editor's comment #5.

Reviewer's #1 comment #4
The title could better reflect what PEMA actually does, and particularly its flexibility.
The title was changed as suggested.

Reviewer's #1 comment #5
Line 73: write "e.g. mothur, QIIME, Lotus". There are many other tools (e.g. DADA2).
Indeed, there are many other tools for the 16S rRNA marker gene analysis. However, we are mentioning these three as we intend to compare PEMA with them. In addition, DADA2 is also mentioned later in the manuscript as one of the QIIME2 features.

Reviewer's #1 comment #6
Line 74-76: Please tone-down this sentence. Many tools are usable for usable for the metabarcoding analysis of eukaryotic organisms like obitools. For a friendly-user software, see also the SLIM pipeline for eDNA metabarcoding data (Dufresne et al. 2019 BMC Bioinformatics).
The sentence was rephrased as suggested.

Reviewer's #1 comment #7
Line 96: Does the pipeline offer flexibility in the primers pair used to amplify this region? If so, what primers can be used? Please add references.
The pipeline is not designed for specific gene regions. The databases used include sequences of the whole marker gene. Thus, metabarcoding data from any region can be analysed through PEMA, as long as the paired end reads can be merged successfully at the relevant step.

Reviewer's #1 comment #8
Line 98: Please describe what VSEARCH does exactly in PEMA.
The sentence was rephrased. Also, since all the tools are described thoroughly in the Supplementary Methods file, more information has been added there.

Reviewer's #1 comment #9
Line 99: Write "Taxonomic assignment"

The sentence was rephrased as suggested.

Reviewer’s #1 comment #10
Line 124: Please describe what are the differences between these options.
The sentence has been removed.

Reviewer’s #1 comment #11
Line 125-130: Please precise here that chimeras are expected to form independent OTUs.
This information has been included in the Supplementary Methods file.

Reviewer’s #1 comment #12
Line 133: Remove the ")"
The sentence was rephrased as suggested.

Reviewer’s #1 comment #13
Line 136: What do you mean by "singletons" here: Sequences not assigned to a mOTU or sequence with just one read?
The term was further explained.

Reviewer’s #1 comment #14
Line 141: Are users able to download new releases?
Not directly. New versions would have to be incorporated by us and the CREST classifier should be trained accordingly, in order to be used for these newer SILVA versions. The two latest Silva versions (v128 and v132) have been included. For future Silva versions (e.g. v138 and onwards), updated Docker and Singularity images of PEMA will be available.

Reviewer’s #1 comment #15
Line 216-218: Does Table 3 reflect only assignment of taxa from positive controls? Please precise. What do these positive controls contained originally?
Table 5 (Table 3 in the previous version of the manuscript) includes taxonomic identifications of the PEMA retrieved MOTUs and makes a comparison between these identifications and taxa that were included in the positive controls of the original publication. We are not fully aware of what these positive controls contained originally as this information is not provided by Bista et al. The only information that they provide is included in the last column of Table 5.

Reviewer’s #1 comment #16
Line 268-270: I disagree with this sentence (see comments above).
Please see Response to Reviewer’s #1 comment #3 and Response to Editor’s comment #5.

Reviewer’s #1 comment #17
Figs. 3 and 4: I am not convinced about the usefulness of these figures as they only present the outputs of PEMA and do not compare the assignments between pipelines.
The assignments of the different pipelines for the published datasets were included in the original submissions as Additional files 4-8 (Additional files 6-10 in the revised version of the manuscript). So, the reader could compare the results of each pipeline.

Reviewer’s #1 comment #18
Table 5: I would suggest converting this table into a graph to improve its readability, and to present it in relation to the salinity to match with its description in the main text. In addition, the legend says that "N=total microbial relative abundance", which does not seem to make sense in this context.
Additional file 4: Table S2 (Table 5 in the previously submitted version of the manuscript) presents the diversity indices (of the Pavloudi et al dataset) of the samples for each one of the tested pipelines. The columns reflect the

total number of OTUs and the relative abundance of these OTUs. Since we are working with high throughput sequencing data, we cannot use the term "OTU abundance" as high throughput sequencing only provides estimates of the relative abundance of OTUs. Therefore, we do not understand what the reviewer means by his comment that "N [...] does not seem to make sense in this context".
Please see also Response to Reviewer's #2 comment #14.

Reviewer's #1 comment #19
In the description of trimmomatic in supplementary methods, what do the authors mean by "technical sequences? Adapter sequences? Please precise. Also, the following sentence is unclear for me, could you please clarify: "Amplicons in particular could cause significant alterations due to their common presence in the end of the reads".
The term "technical sequences" was replaced by "sequence artifacts". The sentence mentioned was also rephrased.

Reviewer's #1 comment #20
There is no information about the RDPclassifier in the supplementary methods.
Information with respect to the RDPClassifier and the Midori database was added in the Supplementary Methods file.

Reviewer's #2 comment #1
I really appreciated the opportunity to review your and your co-authors' manuscript. The metabarcoding bioinformatics pipeline you present is potentially a useful new addition to the numerous pre-existing pipelines such as those you have mentioned within the manuscript. In particular, the use of BigDataScript language addresses many common concerns regarding processing speeds of such large datasets and supports checkpoint files that would be useful in rerunning portions of the pipeline with different parameter iterations. In addition, a variety of commonly used programs and databases are implemented such as Swarm, CROP, VSEARCH, LCAClassifier, RDPClassifier, Silva, and MIDORI, which will serve to increase applicability to wide diversity of metabarcoding projects. The inclusion of phylogeny-based assignment through RAxML and EPA should be especially useful for microbial research. Lastly, I appreciate the distribution of PEMA via Docker and Singularity, which should ease installation efforts, as well as the detailed tutorial PDF available on the GitHub repository. I hope you will find my comments useful in improving the paper.
We thank you for your creative comments.

Reviewer's #2 comment #2
Line 1-2: I would recommend using an alternative title that describes the abbreviated name of the pipeline, PEMA. Although the pipeline does include steps for taxonomic assignment, only (M)OTU-table is currently mentioned. Personally, I do not think the title needs to be so detailed. Something like this would be preferable: "PEMA: Pipeline for Environmental DNA Metabarcoding Analysis of 16S rRNA and COI genes".
The title has been rephrased. Also, see Response to Reviewer's #1 comment #4.

Reviewer's #2 comment #3
Lines 74-75: "However, there is none that can be used in a straightforward way for metabarcoding analysis of eukaryotic organisms. For this to be functional, adaptation to other marker genes (e.g COI) is required." There are many metabarcoding pipelines available for processing COI markers for eukaryotes. The following are just some examples.
a. https://github.com/Hajibabaei-Lab/SCVUC_COI_metabarcode_pipeline
b. https://github.com/alexcrampton-platt/screenforbio-mbc
c. https://journals.plos.org/plosone/article?id=10.1371/journal.pone.0201763
d. https://github.com/cbirdlab/charybdis
e. https://github.com/enormandeau/barque

f. https://github.com/limey-bean/Anacapa
g. https://chewbacca.readthedocs.io/en/dev/
The sentence was rephrased. Also, see Response to Reviewer's #1 comment #5 and Response to Reviewer's #1 comment #6.

Reviewer's #2 comment #4
Although the manuscript is a technical note, I do think that it would benefit readers to include a brief introduction of what environmental DNA and metabarcoding is and what the advantages are over previous methods of biodiversity assessment.
A paragraph was added in the Background to address this.

Reviewer's #2 comment #5
The pipeline uses VSEARCH, Swarm, and CROP, which are common methods for clustering individual sequences into operational taxonomic units (OTUs). More recently, amplicon sequence variants (ASVs) have been proposed to supplant OTUs through the DADA2 software (Callahan et al. 2016). ASVs have a variety of advantages over OTUs in terms of resolution, accuracy, and comparability (https://benjjneb.github.io/dada2/index.html). One of the major problems with OTU clustering is that de novo clustered OTUs are incomparable across studies. Though not necessary for publication, I think PEMA would benefit greatly from implementation of DADA2 or a discussion of the ability to intake ASV tables for taxonomic assignment within PEMA.
We agree with the Reviewer. However, Swarm is also an ASV inferring algorithm, as its authors have mentioned to us in personal communication. The discussion on ASVs and OTUs is ongoing (an example can be found here: https://github.com/benjjneb/dada2/issues/62 and here: https://twitter.com/ambulanzen/status/1187406117942583296). We have also added a relevant section in our manuscript entitled "OTU clustering *vs* ASV inferring".

Reviewer's #2 comment #6
Is there a reason why downstream analysis via phyloseq is not implemented for OTU tables generated from COI sequences?
Analysis via the phyloseq R package is now available for all the marker genes PEMA supports.

Reviewer's #2 comment #7
The writing of the manuscript could be improved. There are a few typos throughout the paper (e.g. "licences" on line 110, extra")" on line 132, etc.) and the following are just a few examples of awkward phrasing or incorrect grammar usage.
a. Lines 71-72: "However, from the output of a sequencer to an amplicon study analysis results, it takes a long way."
b. Lines 112-113: "Beyond this visual inspection and to correct the errors are produced by a sequencer, PEMA incorporates a number of tools."
c. Lines 291-292: "PEMA's user friendliness derives from the easy and with minimal number of installation and execution commands."
The sentences mentioned were rephrased.

Reviewer's #2 comment #8
All abbreviations should be defined upon first use in the manuscript (e.g. ENA-EBI on line 178).
The abbreviation was defined.

Reviewer's #2 comment #9
Table 2 shows PEMA's performance using various values of parameter d in Swarm. There is no discussion of why each value was chosen and why d = 2 was used for comparison.
d = 2 was discussed further because it resulted in a total number of MOTUs similar to the one of the published study. There is no golden standard value of *d*; it should be tested and decided for each dataset. Also, values up to 13 have been used in certain cases [2]. In addition, a relevant section regarding the values of the parameters and how they can affect the results has been added in the manuscript.

Reviewer's #2 comment #10

The manuscript notes, "Certain .fastq files contained very few reads, such as those for sample ERR1308241, and therefore resulted in zero MOTUs upon the completion of PEMA; thus, these samples are not included in Figure 3." However, three samples also appear to not contain any assigned OTUs (ERR1308210, ERR1308240, ERR1308243). Multiple samples appear without any assigned OTUs in Figure 4 as well.

As it can be seen from Additional file 3: Table S1 (Additional file 2 in the previously submitted version of the manuscript), the samples ERR1308210, ERR1308240, ERR1308243 had initial number of reads 711, 1323 and 873 respectively. After the pre-processing, they ended up with 29, 20 and 26 reads. Inevitably, with such low reads, the derived MOTUs were also very low. This is why in figures 3 and 4 it seems as if they have zero MOTUs; in fact, they have 1, 1 and 2 MOTUs (respectively). As mentioned in the originally submitted manuscript, in the case of samples with zero OTUs found, the samples does not appear in the phyloseq figures at all.

Reviewer's #2 comment #11

Table 3 only shows OTUs that were commonly identified to species and genus level in the original and the current studies. It would be useful to also know which taxa from the positive controls, if any, were only identified in either the original or the current study.

Please see Response to Reviewer's #1 comment #15.

Reviewer's #2 comment #12

The discussion about computational times of Table 2 in lines 223-225 should be moved before discussing Table 3.

The manuscript has been corrected accordingly.

Reviewer's #2 comment #13

It is a known issue that mothur can inflate OTU numbers due to lack of overlap between reads. Have you considered using the phylotype command to assign sequences to OTUs based on taxonomy? https://www.mothur.org/wiki/Phylotype

According to Pat Schloss, when we asked him about the number we got (see: https://forum.mothur.org/t/number-of-otus-in-shared-file-637/19992), he replied that "we generally see an inflated number of OTUs when people sequence regions where the two reads do not fully overlap with each other (http://blog.mothur.org/2014/09/11/Why-such-a-large-distance-matrix/)".

However, since nothing similar occurred when analyzing the same dataset with the other tools, we believe that there may be some other issue in mothur. Also, regarding the "phylotype" you mention, it is relevant to the taxonomy assignment and not to the creation of OTUs (where we experienced the over-inflation problem).

Reviewer's #2 comment #14

Table 5 could be included as a supplementary. Such detail is not necessary in the main text.

The table was moved to the supplementary files as suggested. It is now Additional file 4: Table S2.

Reviewer's #2 comment #15

Lines 250-251 state "the general trend of the decreasing number of OTUs with the increasing salinity was observed as it was in [30]. Notably, this result was not observed with the other tested pipelines (Table 5)." It is difficult to observe the trend with regards to salinity because salinity values are not provided for any of the samples. These statements should be better demonstrated with a figure showing the trend for only PEMA results and none of the other pipelines.

The salinity values are provided at the original publication of Pavloudi et al in Table S6. However, in order to facilitate the reader, a graph (Additional file 5: Figure S1) was created showing the linear decrease of the number of OTUs (as derived by PEMA) with salinity.

Reviewer's #2 comment #16

Lines 252-254 state "Furthermore, each of the pipelines resulted in a different taxonomic profile (Additional files 4-6: Figure S1-3) with an extreme case of missing the Order of Betaproteobacteriales (Additional files 7-9: Figure

S4-6)." It is important to explain why this is the case. Can OTUs and taxonomic profiles of complex microbial communities produced via different pipelines really be directly compared in this way? Do the comparisons even make any sense or reveal anything interesting about why they are different? The results from the original study may not represent the "truth" better than these other pipelines. More discussion on these topics is needed for both the 16S and COI comparisons.

We cannot really know why when we use QIIME with DADA2 we end up missing the order Betaproteobacteriales. Each tool and software developed for metabarcoding analyses produces different results which are not directly comparable to one another, This issue has been discussed extensively in the literature. However, we need to apply a certain degree of comparison when we develop new tools. In this case we chose to use previously published studies in order to see if our tool could produce similar results without distorting the final conclusions of the studies. Furthermore, after your suggestions, we added another level of comparison using mock communities. Also, please see Response to Reviewer's #1 comment #3 and Response to Editor's comment #5.

Reviewer's #2 comment #17

In Table 5, please explain what "N: total microbial relative abundance values" is.

Please see Response to Reviewer's #1 comment #18.

Reviewer's #2 comment #18

A general suggestion that there should be at least a brief discussion of the two datasets being used for comparing the various bioinformatics pipelines. What was the goal of the original study, what was the experimental design, why did they sequence those particular samples, etc. This should help readers understand the results of the comparison better.

Generally, we agree with the reviewer. However, we feel that a discussion on the datasets is not crucial for the reader, since we aim to present our pipeline and since the manuscript's length has increased a lot with the analyses on the mock communities. Thus, we added only a few sentences on the reasons that the datasets were chosen. The main reason was that we wanted to use high quality hypothesis-driven public datasets. Also, please see Response to Reviewer's #2 comment #16.

Reviewer's #2 comment #19

In the Declarations sections, "MOTU: Molecular Operational Taxonomic Unit (species equivalent for Eukaryotes)" and "OTU: Operational Taxonomic Unit (species equivalent for prokaryotes)" is not true. Often OTUs are clustered at levels below species-level such that multiple OTUs will assign to a single taxonomy for both prokaryotes and eukaryotes.

We agree with the reviewer that OTUs can represent taxonomic levels below the species level. The declarations were rephrased.

Reviewer's #2 comment #20

Captions for Figures 3-5 should be more detailed and indicate which gene is analyzed and which comparisons they are for. Additionally, Figure 4 is not discussed in the text at all.

The captions of Figures mentioned were explained further as suggested. Figure 4 is now discussed in the text.

Reviewer's #2 comment #21

The use of citations within the text such as in the following sentence is unconventional and inappropriate. Typically, the last name of the first author is used followed by the numbered citations.

a. Lines 175-177: "For the 16S rRNA marker gene, the dataset reported by study [30] was used while for the COI case, the one of [31] (accession numbers: PRJEB20211 and PRJEB13009 respectively)."

b. Lines 196-197: "Regarding the creation of the MOTU table, [31] used VSEARCH [10] with a clustering at 97% similarity threshold."

In the first place, we followed the instructions of the journal in formatting the references and the citations (see: https://academic.oup.com/gigascience/pages/instructions_to_authors). Hence, we used the square brackets. However, we agree with your point of view and we did the suggested changes.

References

[1] Kurtzer GM, Sochat V, Bauer MW. Singularity: Scientific containers for mobility of compute. PloS one. 2017 May 11;12(5):e0177459.

[2] Siegenthaler A, Wangensteen OS, Benvenuto C, Campos J, Mariani S. DNA metabarcoding unveils multiscale trophic variation in a widespread coastal opportunist. Molecular ecology. 2019 Jan;28(2):232-49.
