# Supplementary material for: PEMA: a flexible Pipeline for Environmental DNA Metabarcoding Analysis of the 16S/18S ribosomal RNA, ITS, and COI marker genes
Source: Gigascience. 2020 Mar 12;9(3):giaa022. doi: 10.1093/gigascience/giaa022 (PMC7066391; doi:10.1093/gigascience/giaa022)
Supplement: giaa022_GIGA-D-19-00397_Revision_1 [file giaa022_giga-d-19-00397_revision_1.pdf]

GigaScience

PEMA: a flexible Pipeline for Environmental DNA Metabarcoding Analysis of the 16S/18S rRNA, ITS and COI marker genes

--Manuscript Draft--

**Manuscript Number:**	GIGA-D-19-00397R1
**Full Title:**	PEMA: a flexible Pipeline for Environmental DNA Metabarcoding Analysis of the 16S/18S rRNA, ITS and COI marker genes
**Article Type:**	Technical Note
**Funding Information:**	General Secretariat for Research and Technology (241) — Dr Evangelos Pafilis
**Abstract:**	Background: Environmental DNA (eDNA) and metabarcoding allow the identification of a mixture of species individuals and launch a new era in bio- and eco-assessment. A great number of steps are required to obtain taxonomically assigned matrices from raw data. For most of these, a plethora of tools are available; each tool's execution parameters need to be tailored to reflect each experiment's idiosyncrasy. Adding to this complexity, the computation capacity of High Performance Computing systems is frequently required for such analyses. To address the aforementioned difficulties, bioinformatic pipelines need to combine state-of-the art technologies and algorithms with an easy to get-set-use framework, allowing researchers to tune each study. Software containerization technologies ease the sharing and running of software packages across operating systems; thus, they strongly facilitate pipeline development and usage. Likewise are programming languages specialized for big data pipelines, incorporating features like roll-back checkpoints and on-demand partial pipeline execution.<br>Findings: PEMA is a containerized assembly of key metabarcoding analysis tools with a low effort in setting up, running and customizing to researchers' needs. Based on third party tools, PEMA performs read pre-processing, (M)OTUs clustering, ASV inference, and taxonomy assignment for 16S and 18S rRNA as well as ITS and COI marker gene data. Due to its simplified parameterisation and checkpoint support, PEMA allows users to explore alternative algorithms for specific steps of the pipeline without the need of a complete re-execution. PEMA was evaluated against both mock communities and previously published datasets and achieved comparable quality results.<br>Conclusions: An HPC-based approach was used to develop PEMA, however it can be used in personal computers as well. Given its time-efficient performance and its quality results, it is suggested that PEMA can be used for accurate eDNA metabarcoding analysis, thus enhancing the applicability of next-generation biodiversity assessment studies.
**Corresponding Author:**	Haris Zafeiropoulos<br>Hellenic Centre for Marine Research<br>Heraklion, Irakleio GREECE
**Corresponding Author Secondary Information:**	
**Corresponding Author's Institution:**	Hellenic Centre for Marine Research
**Corresponding Author's Secondary Institution:**	
**First Author:**	Haris Zafeiropoulos
**First Author Secondary Information:**	
**Order of Authors:**	Haris Zafeiropoulos
	Viet Ha Quoc
	Katerina Vasileiadou
	Antonis Potirakis

Powered by Editorial Manager® and ProduXion Manager® from Aries Systems Corporation

	Christos Arvanitidis
	Pantelis Topalis
	Christina Pavloudi
	Evangelos Pafilis
**Order of Authors Secondary Information:**	
**Response to Reviewers:**	Response to the editor’s and reviewer’s comments<br><br>We would like to kindly thank the reviewer for the time he spent to thoroughly read our manuscript. We sincerely appreciate all accurate comments made by the reviewer, which helped the improvement of our manuscript. In the revised version, we have addressed all of the reviewer’s comments and suggestions and where necessary we have incorporated changes and made amendments and alterations to the manuscript. The changes and the new sections of the manuscript are written in blue. Below we cite our detailed answers (in blue) to the editor and reviewers’ comments and suggestions (italic).<br><br><br><br>Editor’s comment #1<br>In particular, the section on comparing the tool with existing pipelines needs more attention with respect to the way this comparison is presented in the manuscript. As the reviewer says, "PEMA allows to switch from one (existing) tool to another at each step of the filtering process, therefore differences are the result of the combination of tools decided by the user, not of PEMA itself."<br>Switching tools without having to re-run an analysis from scratch, is one of PEMA’s main advantages. Providing the user the ability for a great number of tests, while tuning numerous parameters of the tools selected, PEMA allows for a thorough benchmarking for each and every study. Regarding the way that the comparison between PEMA and other metabarcoding pipelines is presented, please see Editor’s comment #2 and Reviewer’s comment #2.<br><br>Editor’s comment #2<br>I do feel that comparisons to similar tools are an important aspect for our Technical Notes, but please make sure that the reader is not getting potentially misleading impressions.<br>A paragraph explaining both the purpose and the limitations of comparing pipelines has been added as a “preface” in the “Evaluation on real datasets and against other tools” section (lines 345-352 of the revised version of the manuscript). Certain changes have been made in the manuscript to clarify any potential misconception regarding this issue (e.g. lines 389-390).<br><br>Editor’s comment #3<br>If you have not yet done so, please register your new software application in the bio.tools and SciCrunch.org databases to receive RRID (Research Resource Identification Initiative ID) and biotoolsID identifiers, and include these in your manuscript. This will facilitate tracking, reproducibility and re-use of your tool.<br>PEMA was not registered in bio.tools or SciCrunc.org databases until the last submission. Now it has been registered in both and has the unique ids: “PEMA” (in biotools) and “SCR_017676” (RRID in SciCrunch.org). They have both been included in the revised PEMA manuscript.<br><br><br><br>Reviewer’s comment #1<br>I have reviewed for the second time the manuscript from Zafeiropoulos et al. "PEMA: a flexible Pipeline for Environmental DNA Metabarcoding Analysis of the 16S/18S rRNA, ITS and COI marker genes". First, I would like to acknowledge the major improvements implemented by the authors in their pipeline since the first submission, namely: the extension of the functionality to two other marker genes (18S rRNA and ITS) and the inclusion of tools for inferring ASV. This is undoubtedly a great set of additional tools, and the pipeline now covers four of the most common markers for eDNA studies (but

not all marker genes as mentioned in the responses). That said, attending that similar pipelines already exist (see PipeCraft for another example; Anslan et al. 2017 Molecular Ecology), I think the main strength of this pipeline is in flexibility, time efficiency and ability to handle large datasets.
Thank you for your patience for a second review. It is the most appreciated.
With respect to PipeCraft, unfortunately we did not achieve to test it. In their paper, Anslan et al (2017) write that it is available through PlutoF system (download link https://plutof.ut.ee/#/datacite/10.15156%2FBIO%2F587450), however the link does not work. In addition, PipeCraft is not registered in any other repository, making it impossible for end-users to reach it.

Reviewer’s comment #2
I appreciate the inclusion of mock community analyses that, contrarily to real eDNA dataset, allow the reader to have an idea of what to expect and easily compare outputs of the pipeline with the biological reality (although this section could gain in readability by avoiding study-specific details). However, I am still very puzzled by the comparisons with other software. I agree with the authors when they say that "it is important to assess the variability that different tools introduce in the produced outputs of each study", and PEMA allows that by offering different options at every step of the pipeline, but it seems abusive to say that results from PEMA outperforms those of other pipelines (e.g. lines 370-372). Comparing pipelines make sense when there is algorithm development but here PEMA allows to switch from one (existing) tool to another at each step of the filtering process, therefore differences are the result of the combination of tools decided by the user, not of PEMA itself.
In my opinion, these comparisons can appear as misleading, and make the paper more complicated and longer than needed.
Regarding the sentence in lines 370-372 (previous version of the manuscript), it has been rephrased (lines 389-390 of the revised version of the manuscript), hopefully not causing misconceptions. In addition, in the paragraph added (preface) in this section, an extended explanation of the rationale of this comparison clarifies this issue (lines 345-352).
However, we would like to specify our point-of-view on this issue. The comparison with other software is one of the prerequisite criteria of the journal’s author guidelines for “Technical Notes” (https://academic.oup.com/gigascience/pages/technical_note). As mentioned there: “The tool or method needs to have been tested, and properly compared to any existing tools or methods used by the community. It does not necessarily have to outperform existing approaches, but it should show innovation in the approach, implementation, or have added benefits that have been needed in this arena.” That is the reason we thought about adding the section “Evaluation on real datasets and against other tools” and run these tests in the first place.
According to the reviewer: “PEMA allows to switch from one (existing) tool to another at each step of the filtering process, therefore differences are the result of the combination of tools decided by the user, not of PEMA itself”. PEMA does not, indeed, contain novel algorithm development. However, being able to select among a richer pool of tools so as to perform a high quality metabarcoding analysis is the very essence of PEMA. PEMA users being able to combine third-party tools (out of a rich set) in an easy way constitutes an “added benefit that has been needed in this arena” (based on the GigaScience journal aim mentioned above). It is this easiness along with more of PEMA distinctive features (such as checkpoints and partial workflow re-execution) that justify the flexibility claimed in the manuscript title “PEMA: a flexible Pipeline for Environmental DNA Metabarcoding”.
By comparing metabarcoding pipelines we cannot argue in a straightforward way that one is better than the other; it is definitely not the aim of this section. By performing these analyses we intend to present the potentials of each of those pipelines as well as their computational needs in each case. More specifically, you cannot say that PEMA is generally faster than any other pipeline; for example, if the CROP algorithm is selected, then for sure the analysis will take a long time. However, a more general view of such analyses does provide the reader with important information and, in all cases, it highlights the importance of its own role when setting the parameters.

Reviewer’s comment #3
Finally, I am still not convinced by the usefulness of Figs. 3, 4 and 5 in the main text. This is a technical note and, without any direct comparison, they do not bring much information. I would suggest to either make a synthetic figure or to move them to

Powered by Editorial Manager® and ProduXion Manager® from Aries Systems Corporation

supplementary material (but there are already many supplementary files).
The main aim of the figures mentioned is to visualize the findings described in the "Evaluation on real datasets and against other tools". We agree with the reviewer's comment that in a technical note they could be avoided and it is our belief that we should remove Figures 3 and 4 at all. However, it is our belief that Figure 5 (Figure 3 in the revised version of the manuscript) should be kept as an example of the visualization that PEMA supports.

Reviewer's comment #4
In further communication, please add line number in the responses to editor/reviewers to indicate where changes have been made.
We have followed the recommendation of the reviewer.

Reviewer's comment #5
Line 74-78: please reformulate, the current definition of metabarcoding is quite vague.
The definition of metabarcoding has been rephrased (lines 76-79 of the revised version of the manuscript).

Reviewer's comment #6
Line 78: it is rather a "potential holistic approach"
The sentence was rephrased (line 81 of the revised version of the manuscript).

Reviewer's comment #7
Line 82-88: for each marker, please explain what taxonomic group(s) it targets. Also, authors could make explicit that any primer pairs amplifying one of these regions can be used as long as paired-end reads can be merged successfully.
With respect to the taxonomic groups, we have followed the recommendation of the reviewer (see lines 76-79). Regarding the primer pairs, it is our belief that as this is a background paragraph describing the metabarcoding method, it would be better not to include it. That is because metabarcoding studies may occur with single end reads as well.

Reviewer's comment #8
Line 87-88: there are already some pipelines for this.
An example of a pipeline for the ITS marker gene has been added (line 90 of the revised version of the manuscript). The main point of this sentence is to underlie the need for a fast and flexible pipeline for those two marker genes (COI and ITS) too.

Reviewer's comment #9
Line 112-115: this paragraph could place later to increase readability (e.g. after the next paragraph or in the discussion)
It is our belief that this paragraph should remain in its current place in the manuscript (lines 117-120 in the revised version of the manuscript). Its aim is to justify what comes to the exact next paragraph; PEMA supports both OTU clustering and ASV inference because of the fact that "The use of ASVs instead of OTUs has been suggested [14], however the choice for which approach to use should rely on each study's objective(s) [15]." Furthermore, we consider that a definition of the ASVs is needed here.

Reviewer's comment #10
Line 137-138: What about samples with a low number of reads? This could be part of the initial quality check.
We have followed the recommendation of the reviewer and changed the sentence accordingly (lines 144-145).

Reviewer's comment #11
Line 164-165: Is there two chimera removal steps: Vsearch in Part 1 and later step in part 3? Or is it only when using Swarm? Can you please explain.
The chimera removal step occurs only once in all cases, as it is also shown in the figure describing the pipeline (Figure 1). What changes is the order of the steps, depending on the algorithms selected by the user. If the Swarm v2 algorithm has been selected, then the chimera removal takes place after the ASV inference. In all other cases, the chimera removal step occurs before the OTU clustering. We added a sentence at the end of the previous section ("Part 1: Quality control and pre-processing of raw data") to clarify this (lines 153-155).

Powered by Editorial Manager® and ProduXion Manager® from Aries Systems Corporation

Reviewer’s comment #12
Lines 238-241: Please reformulate
We have followed the recommendation of the reviewer and rephrased the sentence (lines 247-255).

Reviewer’s comment #13
Line 249 and additional file 2: The description of the tools and parameters used for each dataset (as well as the rationales for choosing them) would be welcomed here.
As mentioned in the manuscript, the tools and the parameters used in each run can be found in the “Additional file 2: Mock communities”. More specifically, a separate sheet for each marker gene can be found in this document, where PEMA's output as long as the corresponding statistics are shown for each tool and parameter set. Regarding the rationales for choosing those mock communities, a paragraph has been added in the “Additional file 2: Mock communities” on the "datasets" sheet.

Reviewer’s comment #14
Line 258-317: This section could be reduced by removing too species-specific details (e.g. lines 283-285).
As we share the common belief for a not excessively long manuscript, we removed any species-specific and mock-community-specific details for the manuscript as possible (e.g. lines 283-285 of the previous version of the manuscript). However, as it is of great importance to distinguish PEMA’s false positives and true negatives from those that occur due to the datasets’ features we kept such details when considered that is needed to that end.

Reviewer’s comment #15
Line 270-273: This paragraph should be moved elsewhere as it is valid for all markers.
We have followed the recommendation of the reviewer. The paragraph has been moved at the end of the previous section (lines 272-276).

Reviewer’s comment #16
Lines 311-314: unclear, please reformulate.
We have followed the recommendation of the reviewer and reformulated the sentence (lines 322-327).

Reviewer’s comment #17
Lines 429-440: References would be welcomed here.
We have followed the recommendation of the reviewer (lines 446-458).

Reviewer’s comment #18
Line 437-440: Please explain more in details what you mean here. I am not sure I fully agree with this statement.
We have followed the recommendation of the reviewer (lines 455-458). The point of this paragraph is to discuss that by making use of ASVs, especially in the case of microbial communities, we might end up with a vast number of sequences, both because of high alpha- (intra-sample variation) and beta (inter-sample variation) diversity. This complicates even more downstream statistical analyses and the drawing of conclusions about the dynamics of the communities under study.

Reviewer’s comment #19
Table 5: This table does not seem necessary now that authors added mock community analyses, especially if the original community is unknown. It seems redundant.
The aim of this study is to show PEMA’s performance from a biological point-of-view in real data, compared to those of Barque. As mentioned in the table’s label, the comparison is against the initial study’s positive controls. Thus, the output of two different pipelines can be compared as the composition of those samples is known. For the case of the Pavloudi et al. dataset, there is no such positive controls and that is why there is not an equivalent table presented.

Reviewer’s comment #20
Table S2 (previously Table 5): I still do not understand what the authors mean by "N = total microbial relative abundance". It seems to me that these numbers represent the number of reads? If so, the term "relative abundance" is inappropriate and confusing

Powered by Editorial Manager® and ProduXion Manager® from Aries Systems Corporation

	(one would expect a percentage).<br>High throughput sequencing can only provide relative estimates of abundances. Absolute abundances can be provided by other methods, such as quantitative PCR (Q-PCR). Therefore, it is well established that high throughput sequencing can only result in relative abundances of taxa.<br>"N" has been used extensively among biodiversity scientists to denote the "total number of individuals" of a given taxon, therefore its "total abundance" (for example, see: 10.1016/S0022-0981(98)00028-8). Thus, in our case, as well as in other studies where it has been used to describe metabarcoding data, it denotes the "total microbial relative abundance values" (see: 10.7717/peerj.3687).<br><br>For example, consider the example below, of a classic abundance table (taxa per stations):<br><br><br>,Station A,Station B<br>Taxon 1,15,22<br>Taxon 2,0,52<br>Taxon 3,2,34<br><br>In this example case, when one would calculate the classic biodiversity indices, the result would be the following:<br><br>,S (number of taxa),N (total abundance)<br>Station A,2,17<br>Station B,3,108<br>SUM,5,125<br><br>If these indices were to be presented as percentages, the result would be:<br><br>,Percentage of each station's number of taxa to the sum of the number of taxa found in all the stations of the study,Percentage of each station's total abundance of taxa to the sum of the total abundances of taxa found in all the stations of the study<br>Station A,40 %,13.6 %<br>Station B,60 %,86.4 %<br><br>The logic is the same, however we cannot use the term "total abundance". These numbers do not just represent the number of reads, since the reads have undergone all the necessary processing (which is thoroughly explained in the manuscript) in order to derive to the final (M)OTU/ASV table (the equivalent of the classic abundance table in the case of eDNA metabarcoding). The number of sequences, i.e. reads) after each pre-processing step are shown in Additional file 3: Table S1.
**Additional Information:**	
**Question**	**Response**
Are you submitting this manuscript to a special series or article collection?	No
**Experimental design and statistics**<br><br><br>Full details of the experimental design and statistical methods used should be given in the Methods section, as detailed in our Minimum Standards Reporting Checklist. Information essential to interpreting the data presented should be made available in the figure legends.	Yes

Powered by Editorial Manager® and ProduXion Manager® from Aries Systems Corporation

Have you included all the information requested in your manuscript?	
**Resources**<br><br>A description of all resources used, including antibodies, cell lines, animals and software tools, with enough information to allow them to be uniquely identified, should be included in the Methods section. Authors are strongly encouraged to cite Research Resource Identifiers (RRIDs) for antibodies, model organisms and tools, where possible.<br><br>Have you included the information requested as detailed in our Minimum Standards Reporting Checklist?	Yes
**Availability of data and materials**<br><br>All datasets and code on which the conclusions of the paper rely must be either included in your submission or deposited in publicly available repositories (where available and ethically appropriate), referencing such data using a unique identifier in the references and in the “Availability of Data and Materials” section of your manuscript.<br><br>Have you have met the above requirement as detailed in our Minimum Standards Reporting Checklist?	Yes

Powered by Editorial Manager® and ProduXion Manager® from Aries Systems Corporation

Manuscript Click here to access/download;Manuscript;PEMA_forpublication.docx

PEMA: a flexible Pipeline for Environmental DNA Metabarcoding Analysis of the 16S/18S rRNA, ITS and COI marker genes

Haris Zafeiropoulos*

Institute of Marine Biology, Biotechnology and Aquaculture (IMBBC), Hellenic Centre for Marine Research (HCMR), Heraklion, Greece

e-mail: haris-zaf@hcmr.gr

*** Corresponding author**

Ha Quoc Viet

Institute of Marine Biology, Biotechnology and Aquaculture (IMBBC), Hellenic Centre for Marine Research (HCMR), Heraklion, Greece

e-mail: qvha@free.fr

Katerina Vasileiadou

Charles University, Prague, Czech Republic

Institute of Marine Biology, Biotechnology and Aquaculture (IMBBC), Hellenic Centre for Marine Research (HCMR), Heraklion, Greece

e-mail: kvasileiadou@hcmr.gr

Antonis Potirakis

Institute of Marine Biology, Biotechnology and Aquaculture (IMBBC), Hellenic Centre for Marine Research (HCMR), Heraklion, Greece

e-mail: potant@hcmr.gr

Christos Arvanitidis

Institute of Marine Biology, Biotechnology and Aquaculture (IMBBC), Hellenic Centre for Marine Research (HCMR), Heraklion, Greece

e-mail: arvanitidis@hcmr.gr

Pantelis Topalis

Institute of Molecular Biology and Biotechnology (IMBB), Foundation for Research and Technology (FORTH), Heraklion, Greece

e-mail: topalis@imbb.forth.gr

Christina Pavloudi

Institute of Marine Biology, Biotechnology and Aquaculture (IMBBC), Hellenic Centre for Marine Research (HCMR), Heraklion, Greece

e-mail: cpavloud@hcmr.gr

Evangelos Pafilis

Institute of Marine Biology, Biotechnology and Aquaculture (IMBBC), Hellenic Centre for Marine Research (HCMR), Heraklion, Greece

e-mail: pafilis@hcmr.gr

Abstract

Background: Environmental DNA (eDNA) and metabarcoding allow the identification of a mixture of species and launch a new era in bio- and eco-assessment. A great number of steps are required to obtain taxonomically assigned matrices from raw data. For most of these, a plethora of tools are available; each tool's execution parameters need to be tailored to reflect each experiment's idiosyncrasy. Adding to this complexity, the computation capacity of High Performance Computing systems is frequently required for such analyses. To address the difficulties, bioinformatic pipelines need to combine state-of-the art technologies and algorithms with an easy to get-set-use framework, allowing researchers to tune each

study. Software containerization technologies ease the sharing and running of software packages across operating systems; thus, they strongly facilitate pipeline development and usage. Likewise programming languages specialized for big data pipelines incorporate features like roll-back checkpoints and on-demand partial pipeline execution.

Findings: PEMA is a containerized assembly of key metabarcoding analysis tools with a low effort in setting up, running and customizing to researchers' needs. Based on third party tools, PEMA performs read pre-processing, (M)OTUs clustering, ASV inference, and taxonomy assignment for 16S and 18S rRNA as well as ITS and COI marker gene data. Due to its simplified parameterisation and checkpoint support, PEMA allows users to explore alternative algorithms for specific steps of the pipeline without the need of a complete re-execution. PEMA was evaluated against both mock communities and previously published datasets and achieved comparable quality results.

Conclusions: An HPC-based approach was used to develop PEMA, however it can be used in personal computers as well. Given its time-efficient performance and its quality results, it is suggested that PEMA can be used for accurate eDNA metabarcoding analysis, thus enhancing the applicability of next-generation biodiversity assessment studies.

Keywords:

Pipeline, Container, Docker, Singularity, High Performance Computing, HPC, eDNA, metabarcoding

Background

Environmental DNA (eDNA) metabarcoding inaugurates a new era in bio- and eco-monitoring [1]. eDNA, i.e. genetic material obtained directly from environmental samples (soil, sediment, water, etc.) without any obvious signs of biological source material [2]. Metabarcoding is the combination of DNA taxonomy, based on taxa specific marker genes (e.g. 16S rRNA for Bacteria and Archaea, COI and 18S rRNA for Metazoa, ITS for Fungi), and high-throughput DNA sequencing technologies; thus, simultaneous identification of a mixture of organisms is attainable [3]. eDNA metabarcoding attempts to turn the page into the way biodiversity is perceived and monitored [3]. Their combination is considered to be a potential

holistic approach that, once standardized, allows for higher detection capacity and at a lower cost compared to conventional methods of biodiversity assessment. However, from the raw reads sequence files to an amplicon study's results, the bioinformatics analysis required can be troublesome for many researchers.

Well-established pipelines are available to process metabarcoding data for the case of 16S and 18S rRNA marker genes and bacterial communities (e.g. mothur [4], QIIME 2 [5], LotuS [6]). However, certain limitations accompany each of those and occasionally they can be far from easy-to-use software. Moreover, there is a great need for similarly straightforward and benchmarked approaches for the analysis of other marker genes. With respect to the COI and the ITS marker genes, a number of pipelines have been implemented, e.g. Barque [7], ScreenForBio [8] etc. and PIPITS [9] correspondingly. However, there is still need for a fast, flexible, easy-to-install and easy-to-use pipeline for both, COI and ITS marker genes. The pipelines mentioned above, although entrenched, still suffer from a series of hurdles: technical difficulties in installation and usage, strict limitations in setting parameters for the algorithms invoked, incompetence in partial re-execution of an analysis, are among the most prominent.

Moreover, given the computational demands of such analyses, access to High Performance Computing (HPC) systems might be mandatory, for example, to process studies with large number of samples. This is rather timely given the ongoing investment of national and international efforts (for example [10]) to serve the broad biological community via commonly accessible infrastructures.

PEMA is an open-source pipeline that bundles state-of-the-art bioinformatic tools for all necessary steps of amplicon analysis and aims to address the issues mentioned above. It is designed for paired-end sequencing studies and is implemented in the BigDataScript (BDS) [11] programming language. BDS's *ad hoc* task parallelism and task synchronization supports heavyweight computation which PEMA inherits. In addition, BDS supports *checkpoint* files that can be used for partial re-execution and crash recovery of the pipeline. PEMA builds on this feature to serve tool and parameter exploratory customization for optimal metabarcoding analysis fine tuning. Switching effortlessly between (Molecular) Operational Taxonomic Units ((M)OTUs) clustering and Amplicon Sequence Variants (ASVs) inference algorithms is a pertinent example. Finally, via software containerization technologies such as Docker [12] and Singularity [13], with the latter being HPC-centered, PEMA is distributed in an easy to download and

install fashion on a range of systems, from regular computers to cloud or HPC environments.

From the biological perspective, monitoring biodiversity at all its different levels is of great importance. As there is not a single marker gene to detect all taxa, researchers need to use different genes targeting each great taxonomy group separately [14]. To that end, PEMA supports the metabarcoding analysis of both prokaryotic communities, based on the 16S rRNA marker gene, and eukaryotic ones, based on the ITS (for Fungi), and COI and 18S rRNA (for Metazoa) marker genes [14].

As High Throughput Sequencing (HTS) data become more and more accurate, ASVs, i.e. marker gene amplified sequence reads that differ in at least one nucleotide to each other, become easier to resolve [15]. The use of ASVs instead of OTUs has been suggested [15], however the choice for which approach to use should rely on each study's objective(s) [16].

PEMA supports both OTU clustering and ASV inference for all marker genes (see "OTU clustering *vs* ASV inference" in the "Results and discussion" section). Two clustering algorithms, VSEARCH [17] and CROP [18], are employed for the clustering of reads in (M)OTUs; the former for the case of the 16S/18S rRNA marker genes, the latter for the case of COI and ITS. Swarm v2 [19] allows ASV inference in all cases.

Taxonomic assignment is performed in an alignment-based approach, making use of the CREST LCAClassifier [20] and the Silva database [21] for the case of 16S and 18S rRNA marker genes; the Unite database [22] is used for the ITS gene. In the 16S marker gene case, phylogeny-based assignment is also supported, based on RAxML-ng [23], EPA-ng [24] and Silva [21]. For the COI marker gene, the RDPClassifier [25] and the MIDORI database [26] are used for the taxonomic assignment. In addition, ecological and phylogenetic analysis are facilitated via the "phyloseq" R package [27].

All the pipeline- and third-party-module-controlling parameters are defined in a plain *parameter-value pair* text file. Its straightforward format eases the analysis fine tuning, complementary to the aforementioned *checkpoint* mechanism. A tutorial about PEMA and installation guidance can be found on PEMA's GitHub repository (https://github.com/hariszaf/pema).

Implementation

PEMA's architecture comprises four main parts taking place in tandem (Figure 1). A detailed description of the tools invoked by PEMA and their licenses is included in Additional file 1: Supplementary Methods.

Part 1: Quality control and pre-processing of raw data

First, FastQC [28] is used to obtain an overall read-quality summary; the visual inspection of each sample's quality may recommend to remove those with utterly poor quality as well as samples with a low number of reads and run again the analysis. To correct errors produced by the sequencer, PEMA incorporates a number of tools. Trimmomatic [29] implements a series of trimming steps, namely: either to remove parts of the sequences corresponding to the adapters or the primers, or to trim and crop parts of the reads, or even remove a read completely, when it fails to reach the quality filtering standards set by the user. Cutadapt [30] is used additionally for the case of ITS to address the variability in length of this marker gene (see Additional file 1: Supplementary Methods). BayesHammer [31], an algorithm of the SPAdes assembly toolkit [32], revises incorrectly called bases. PANDAseq [33] assembles the overlapping paired-end reads and then the 'obiuniq' program of OBITools [34] groups all the identical sequences in every sample, keeping a track of their abundances. The VSEARCH package [17] is invoked for the chimera removal then; however, if the Swarm v2 algorithm is selected, this step will be performed after the ASV inference (see in the section below).

Part 2: (M)OTUs clustering and ASV inference

Quality controlled and processed sequences are subsequently clustered into (M)OTUs or treated as input for inferring ASVs. For the case of 16S and 18S rRNA marker genes, VSEARCH [17] is used for the case of OTU clustering, while ASVs can be identified by the Swarm v2 algorithm [19]. VSEARCH is an accurate and fast tool that can handle large datasets; at the same time it is a great alternative of USEARCH [35] as it is distributed under an open source license.

For the ITS and COI marker genes, CROP [18], an unsupervised probabilistic Bayesian clustering algorithm that models the clustering process using Birth-death Markov chain Monte Carlo (MCMC). The CROP clustering algorithm is adjusted by a series of parameters need to be tuned by the user (namely *b*, *e*

and *z*). These parameters depend on specific dataset properties like the length and the number of reads. PEMA, automatically adjusts *b, e* and *z* by collecting such information and applying the CROP recommended parameter-setting rules [18]. ASV inference is conducted by Swarm v2 [19] in this case too.

As the Swarm v2 algorithm is not affected by chimeras (F. Mahé, personal communication), when Swarm v2 is selected, chimera removal occurs after the clustering (see Additional file 1: Supplementary Methods: Swarm v2). This leads to a computational time gain as chimeras are sought among ASVs, instead of ungrouped reads.

Last, any singletons, i.e. sequences with only one read, occurring after the (M)OTU clustering or the ASV inference, may be removed according to the user's parameter settings.

Part 3: Taxonomy assignment

Alignment-based taxonomy assignment is supported for all marker gene analyses. In the case of the 16S/18S rRNA and ITS marker genes, the LCAClassifier algorithm of the CREST set of resources and tools [20], is used together with the Silva [21] and the Unite [22] database, respectively, to assign taxonomy to the OTUs. Two versions of Silva are included in PEMA: 128 (Sept 29, 2016) and 132 (Dec 13, 2017). As classifiers need first to be trained for each database they use, for future Silva [21] versions new PEMA versions will be available.

For the COI marker gene, PEMA uses the RDPClassifier [25] and the MIDORI reference database [26] to assign taxonomy of the MOTUs. The MIDORI database contains quality controlled metazoan mitochondrial gene sequences from GenBank [36].

Intended primarily for studies from less explored environments, phylogeny-based assignment is available for 16S rRNA marker gene data. PEMA maps OTUs to a custom reference tree of 1000 Silva-derived consensus sequences (created using RAxML-ng [23] and gappa (phat algorithm) [37], Figure 2A). PaPaRa [38] and EPA-ng [24] combine the OTU clustering output and the reference tree to produce a phylogeny-aware alignment and map the 16S rRNA OTUs to the custom reference tree. Beyond the context of PEMA, users may visualize the output with tree viewers like iTOL [39] (Figure 2B).

Part 4: Ecological downstream analysis of the taxonomy assigned (M)OTU/ASV tables

PEMA's major output is either an (M)OTUor an ASV table with the assigned taxonomies and the abundances of each taxon in every sample. For each sample of the analysis, a subfolder containing statistics about the quality of its reads, as well as the taxonomies and their abundances, is also returned.

Via the "phyloseq" R package [27], downstream ecological analysis of the taxonomically assigned OTUs or ASVs is supported. This includes alpha- and beta-diversity analysis, taxonomic composition, statistical comparisons and calculation of correlations between samples.

When selected, in addition to the phyloseq's [27] output, a Multiple Sequence Alignment (MSA) and a phylogenetic tree of the OTU/ASVs retrieved can be returned; for the MSA, the MAFFT [40] aligner is invoked while the latter is being built by RAxML-ng [23].

PEMA container-based installation

An easy way of installing PEMA is via its containers. A dockerized PEMA version is available at https://hub.docker.com/r/hariszaf/pema. Singularity users can *pull* the PEMA image from https://singularity-hub.org/collections/2295. Between the two containers, the Singularity-based one is recommended for HPC environments due to Singularity's improved security and file accessing properties [41]. PEMA can also be found in *bio.tools* (id: PEMA) and *SciCruch* (RRID: SCR_017676) databases. For detailed documentation, visit https://github.com/hariszaf/pema.

PEMA output

All PEMA-related files (i.e. intermediate files, final output, *checkpoint* files and per-analysis-parameters) are grouped in distinct (self-explanatory) subfolders per major PEMA pipeline step. In the last subfolder, i.e. subfolder 8, the results are further split in folders per sample. This eases further analysis both within the PEMA framework (like partial re-execution for parameter exploration) or beyond. An extra subfolder is created when an ecological analysis via the "phyloseq" package has been selected.

Results and discussion

Evaluation

To evaluate PEMA, two approaches were followed. First, PEMA was benchmarked against mock community datasets. Second, PEMA was used to analyse previously published datasets. PEMA's output was then compared with the original study outcome as well as with the output of QIIME2, Lotus, Mothur and Barque (where applicable).

Four mock communities, one for each marker gene were used. With respect to the 16S rRNA marker gene, a mock community of *Gohl* et al. [42] with 20 different bacterial species was studied. Correspondingly, in the case of 18S rRNA marker gene, a mock community of *Bradley* et al. [43] with 12 algal species was used; for the ITS, one of *Bakker* [44] including 19 different fungal taxa and for the case of the COI marker gene, a mock community of *Bista* et al. [45] containing 14 metazoan species. More information on the mock communities, their original studies and the results of PEMA for various combinations of parameters can be found in Additional File 2: Mock Communities.

Complementary to the mock community evaluation, two publicly available datasets from published studies were investigated through PEMA. For the 16S rRNA marker gene, the dataset reported by *Pavloudi* et al. [46] was used; the original study aimed at investigating the sediment prokaryotic diversity along a transect river-lagoon-open sea. For the COI case, the one of *Bista* et al. [47] was used; in this study it was investigated whether eDNA can be used for the accurate detection of chironomids (a taxonomic group of macroinvertebrates) in a freshwater habitat.

In both approaches, the respective .fastq files were downloaded from the European Nucleotide Archive (ENA) of the European Bioinformatics Institute ENA-(EBI) using 'ENA File Downloader version 1.2' [48] and PEMA was run on the in-house HPC cluster.

All analyses were conducted on identical Dell M630 nodes (128GB RAM, 20 physical Intel Xeon 2.60GHz cores).

Mock community evaluation

PEMA was tested against mock communities. To evaluate its accuracy it is required to capture: a. how

many of PEMA's predictions that are indeed true (i.e. the percent of correctly assigned taxa among all the predicted ones), and b. how many out of a mock community's taxa were recovered successfully by PEMA. The *precision* statistical metric was used to assess the former and *recall for* the latter. In addition, the *F1-score* was used as a combined metric of both precision and recall. In finer detail *precision* is the ratio of true positives (TP) over the total number of true (TP) and false positives (FP) predicted by a model (*precision = TP / (TP + FP)*); *recall*, is the ratio of TP over the total number of TP and false negatives (FN) (*recall = TP / (TP + FN)*). The *F1-score* is the precision and recall harmonic mean and equals *F1 = 2*(precision*recall)/(precision+recall)* [49].

Table 1: Summary benchmark of PEMA marker-gene specific mock community recovery (precision)

marker gene	precision	recall	F1
16S rRNA	0.81	0.85	0.83
18S rRNA	0.75	0.90	0.82
ITS	0.79	0.94	0.86
COI	0.62	0.93	0.74

Adequate accuracy was achieved when PEMA was used to recover the marker gene specific mock communities at the genus level. Precision and recall scores of ~80% or more are observed with two exceptions in precision but also three very high scores in recall. Overall the precision and recall harmonic mean (F1) scores range from 74% to 86%. A detailed description of the benchmark methodology and statistics analysis is given in the Additional file 2: Mock Communities.

Detailed presentation of per-marker-gene-specific mock community recovery via PEMA is provided in the following sections. A number of different sets of parameters was chosen for each marker gene. Each marker gene has special features (length variability, sequence variability etc.) and each Illumina run has its own intrinsic biases (primers used, PCR protocol etc.); thus, parameters' tuning plays a crucial part in metabarcoding analyses.

In an attempt to analyze thoroughly the sequence data from the mock communities, various sets of parameters were tested based on the experimental details of the published studies but also in an exploratory

way. A great number of different parameter settings were tested, especially for the steps of quality trimming of the reads and the OTU clustering / ASV inference. The differences of their output indicate how sensitive this method is, as well as the great need of a mock community in every metabarcoding study; both as a control but also as a "tuning system" for the parameter setting of the pipeline used.

16S rRNA

When PEMA was performed with the Swarm v2 algorithm (*d* = 3, strictness = 0.6) and the singletons were not removed, 18 out of the 20 taxa were identified to the genus level; 3 of them even to the species level. There were 2 species that were not found in any of the PEMA runs. According to *Gohl* et al. [42], there was a discrepancy in the identification of those two species which was dependent on the amplification protocol used. It is worth mentioning that as *d* increases, taxa cannot be identified to species level at all; however, there is a great shrinkage of the false positive assignments. Thus, when *d* = 30 and strictness = 0.6 for the KAPA samples, *Enterococcus* is not identified at all, however PEMA gets its greatest F1 value (at the genus level, see Table 1), as the false positive assignments returned are minimized. When PEMA was run using the VSEARCH clustering algorithm, high precision values were returned in all cases (>0.79). However, the recall values were decreased when using Swarm v2 (0.65 - 0.68).

18S rRNA

When PEMA was performed using Swarm v2 algorithm (*d* = 1 , strictness = 0.5), 3 out of 12 community members were identified to species level (*Isochrysis galbana*, *Nannochloropsis oculata* and *Thalassiosira pseudonana*), 6 to genus and the rest 3 to class; the latter were all the green algae species (Chlorophyta) of the mock community. However, a better F1 score (0.82) was achieved when the class of Chlorophyceae was not found at all (*d* = 1, strictness = 0.3) as the false positives were decreased to only 1. When the VSEARCH algorithm was used, *Isochrysis galbana* was identified only to the genus level, the *Nannochloropsis* to the order level (Eustigmatales) and the *Poterioochromonas* genus to its class (Chrysophyceae).

ITS

Running PEMA by making use of the Swarm v2 algorithm (d = 20) and targeting the ITS2 region, ASVs from 5 of the 19 species of the mock community were assigned to species level, 10 to genus, 2 to family and 2 to class level. Contrary to Bakker's study [44], PEMA identified the genus *Chytriomyces* in all three samples, as well as the Ustilaginaceae family. Only one false positive assignment was recorded. When the CROP algorithm was used, PEMA's output was less accurate; the *Fusarium* species contained in the mock community were not identified further than their family (Nectriaceae). As mentioned in Bakker's study [44], many reads deriving from the *Fusarium* spp. were not assigned to species level because of the quality trimming step. In addition, a manually assemble reference database for the taxonomy assignment was used in the initial study, containing only sequences of the mock community species, which biased this step and cannot be directly comparable to our case.

COI

Running PEMA on *Bista* et al. dataset [45] and using Swarm v2 (d = 10) identified 12 out of the 14 species included in the mock community. The sole non-identified species were *Bithynia leachii* and *Anisus vortex*. For *B. leachii* no entry exists in the MIDORI database, version MIDORI_LONGEST_1.1. However, the existence of another species of the genus *Bithynia* was recorded. With respect to *A. vortex*, PEMA returned a high abundance ASV assigned to the *Anisus* genus but with a low confidence level. PEMA managed to identify all the members of the mock community. This includes *Physa fontinalis*, originally not designed to be a member of the mock community but as *Bista* et al. [45] explain, was recorded due to cross-contamination. In the case of COI marker gene, unique sequences with low abundances (singletons or doubletons) often lead to spurious MOTUs/ASVs. Thus, as shown in the Additional file 2: Mock Communities, the false positives assignments are decreased when these low abundant sequences are removed; also, the abundance of the assignments (ie. read counts) retrieved can indicate false positive assignments. Thus, true positive assignments occur in greater abundance, with hundreds or even thousands of reads. Contrary to most of the false positives whose abundance is less than 10 read counts. That is mostly for the case of the COI marker gene, as Eukaryotes are under study; Eukaryotes have a great number

of copies of this marker gene - different number of copies among the different species - and not just a single one as it is almost always the case in Bacteria. Therefore, assignments with such low abundances should be doubted as true positives in analyses on real datasets.

Comparison to existing software

By the means of evaluation, PEMA's features were compared with those of mothur [4], QIIME 2 [5], LotuS [6] and Barque [7]. Table 2 presents a detailed comparison among the four tool features in terms of marker gene support, diversity and phylogeny analysis capability, parameter setting and mode of execution, operation system availability and HPC suitability. As shown, PEMA is equally feature-rich, if not richer in certain feature categories, to the other software packages. In particular, PEMA's support for COI marker gene studies is distinctive; two methods for the taxonomy assignment are supported and PEMA's easy-parameter setting, step-by-step execution and container distribution render it user and analysis friendly.

Table 2: Pipeline comparison.

Feature	LotuS	QIIME 2	mothur	Barque	PEMA
16S rRNA	☑	☑	☑	☐	☑
18S rRNA	☑	☑	☑	☐	☑
ITS	☑	☑	☐	☐	☑
COI	☐	☐	☐	☑	☑
diversity indices	☐	☑	☑	☐	☑
alignment-based taxonomy assignment	☑	☑	☑	☑	☑
phylogenetic-based taxonomy assignment	☑	☑	☐	☐	☑
parameters assigned in the command line	☑	☑	☑	☐	☐

parameters assigned through a text file	☑	☐	☐	☑	☑
step-by-step execution	☑	☑	☑	☐	☑
all steps in one go possible	☑	☐	☐	☑	☑
available for any Operating System (Linux, OSX, Windows)	☐	☑	☑	☐	☑
traditional application installation	☑	☑	☑	☑	☑
available as a virtual machine	☐	☑	☐	☐	☐
available as a container	☐	☑	☐	☐	☑
available for HPC as a container (Singularity container)	☐	☐	☐	☐	☑

Comparison of the basic features of the different pipelines.

Evaluation on real datasets and against other tools

In the following sections, a comparative study on real datasets of the 16S rRNA and COI marker genes is presented. PEMA and the pipelines mentioned above that support each of those two marker genes were performed, both with multiple sets of parameters. It is typical for pipelines to invoke a variety of established tools. In many cases, a number of tools are common among different pipelines. Therefore, it is of great importance to stress that such comparisons should be taken into account not in a strict way; claiming that a pipeline is better than another is not trivial to declare. Potentials, limitations of both the pipelines and the metabarcoding method, as well as the importance of the role of the pipeline-user are underlined in the following sections.

16S rRNA marker gene analysis evaluation

To evaluate PEMA's performance, a comparative analysis of the *Pavloudi* et al. [46] dataset with mothur [4], QIIME 2 [5], LotuS [6] and PEMA was conducted.

It is known that the choice of parameters affects the output of each analysis; therefore, it is expected that different user choices might distort the derived outputs. For this reason and for a direct comparison of the pipelines, we have included all the commands and parameters chosen in the framework of this study in the

Additional file 1: Supplementary Methods. The results of the processing of the sequences by PEMA are shown in Additional file 3: Table S1. All analyses were conducted on identical Dell M630 nodes (128GB RAM, 20 physical Intel Xeon 2.60GHz cores). LotuS, mothur and QIIME 2 operated in a single thread (core) fashion. PEMA, given the BDS intrinsic parallelization [11], operated with up to the maximum number of node cores (in this case 20).

The execution time and the reported OTU number of each tool are presented in Table 3. LotuS and PEMA resulted in a final number of OTUs comparable to that of *Pavloudi* et. al [46]. Clearly, due to PEMA's parallel-execution support, the analysis time can be significantly reduced (~1.5 hours in this case). The executional time is depending on the parameters chosen for each software (see Additional file 1: Supplementary Methods).

Table 3: OTU predictions and executional time for the different pipelines.

	LotuS	mothur	QIIME 2		PEMA	*Pavloudi* et al. [46]
			Deblur	DADA2		
Number of OTUs	9849	142669	517	1023	6028	7050
Executional time (h)	~9	~67 (~56 if the reference database is already built)	~2.5	~5	~1.5	~26

Due to the non-full overlap of the sequence reads, mothur resulted in an inflated number of OTUs; thus, is was excluded from further analyses. The results of all the pipelines were analyzed with the phyloseq script that is provided with PEMA. The taxonomic assignment of the PEMA retrieved OTUs is shown in Figure 3. The phyla that were found in the samples are similar to the ones that were found in the original study [46]. Although the lowest number of OTUs was found in the marine station (Kal) (Additional file 4:

Table S3), which is not in accordance with *Pavloudi* et. al [46], the general trend of the decreasing number of OTUs with the increasing salinity was observed as it was in the original study (Additional file 5: Figure S1). Notably, this result was not observed with the other tested pipelines (Additional file 4: Table S3). Furthermore, each of the pipelines resulted in a different taxonomic profile (Additional files 6-8: Figure S2-4) with an extreme case of missing the Order of Betaproteobacteriales (Additional files 9-11: Figure S5-7).

Moreover, when the PERMANOVA analysis was run for the results of PEMA, LotuS and DADA2, it was clear that the microbial community composition was significantly different in each of the three sampled habitats (i.e. River, Lagoon, Sea) (PERMANOVA: F.Model = 7.0718, $p < 0.001$; F.Model = 6.5901, $p < 0.001$, F.Model = 2.2484, $p < 0.05$, respectively), which is in accordance with *Pavloudi* et. al [46]. However, this was not the case with Deblur (PERMANOVA: $p > 0.05$). Overall, PEMA's output is in accordance with the original study [46], and seen through this perspective PEMA performed equally well with the other tested pipelines, along with having the shortest execution time.

COI marker gene analysis evaluation

Bista et al. [47] created two COI libraries of different sizes: COIS (235 bp amplicon size) and COIF (658 bp amplicon size). The sequencing reads of COIS were selected for PEMA's evaluation; the COIF sequencing read pairs had no overlap so as to be merged and therefore were not considered appropriate for the analysis.

As previously, PEMA's performance was evaluated through a comparative analysis of the *Bista* et al. [47] dataset with Barque [7]; the commands and parameters chosen can be found in the Additional file 1: Supplementary Methods. Regarding the creation of the MOTU table, in the *Bista* et al. [47] study VSEARCH [17] was used with a clustering at 97% similarity threshold. Afterwards, the BLAST+ (megablast) algorithm [50] was used against a manually created database including all NCBI GenBank COI sequences of length >100 bp (June 2015) while excluding environmental sequences and higher taxonomic level information [47]. As discussed in the publication, this approach resulted in 138 unique MOTUs out of which 73 were assigned to species level. For PEMA's evaluation, the chosen clustering

algorithm was Swarm v2, using different options for the cluster radius (*d*) parameter (Table 4); according to *Mahé* et al. [19], this is the most important parameter as it affects the number of MOTUs that are being created. The resulting MOTUs were classified against the MIDORI reference database [26] using RDPClassifier [25]. The results of the processing of the sequences are shown in Additional file 12: Table S3. For the case of Barque, the BOLD Database was used [51].

As shown in Table 4, PEMA resulted in 83 species level MOTUs with a cluster radius (*d*) of 2, which is very similar to that of the published study (i.e. 73 species). Although both the clustering algorithm and the taxonomy assignment methods were different between the original [47] and the present study, the results regarding the number of unique species present in the samples are in agreement to a considerable extent.

The computational time required by PEMA for the completion of the analysis is also shown in Table 4. Regardless of the value of the *d* parameter, all analyses were completed in about 2 hours, ie. adequately fast to allow parameter testing and customization. Regarding Barque, the analysis resulted in the identification of 51 species level MOTUs and was concluded in 15 minutes. This difference is due to the error correction step of PEMA (BayesHammer algorithm [31]) which plays an important part in the enhanced results PEMA returns but it also requires a certain computational time; Barque does not have an analogous step, therefore its overall executional time is shorter.

Table 4: PEMA's output and executional time.

	***d* = 1**	***d* = 2**	***d* = 3**	***d* = 10**	***d* = 13**
MOTUs after preprocess and clustering steps	83791	59833	33227	7384	4829
MOTUs after chimera removal	80347	57863	32539	7339	4796
Non singletons MOTUs	6381	4947	2658	1914	1634
Assigned species	62	83	86	86	84
Executional time (h)	02:01:35	02:09:49	01:51:44	02:17:26	02:31:15

PEMA's output and executional time (using a 20 core node) for different values of Swarm's *d* parameter.

PEMA performed better in identifying taxa that were included in the positive control contents of the published study than Barque (Table 5).

Table 5: Comparison of the taxonomy of retrieved MOTUs among PEMA, Barque and the positive controls of *Bista* et al. [47].

**Barque**	**PEMA**	***Bista* et al.** [47]
*Ablabesmyia monilis**	*Ablabesmyia monilis**	*Ablabesmyia monilis*
	*Crangonyx pseudogracilis**	*Crangonyx pseudogracilis*
	Radix sp.*	*Radix* sp.
	Chironomidae sp.*	Chironomidae sp.
	Ancylus sp.**	*Ancylus fluviatilis*
	Athripsodes aterrimus, *Athripsodes cinereus***	*Athripsodes albifrons*
*Chironomus anthracinus***	*Chironomus* sp., *Chironomus anthracinus*, *Chironomus pseudothummi*, *Chironomus riparius***	*Chironomus tentans*
*Polypedilum sordens***		*Polypedilum nubeculosum*
*Athripsodes aterrimus***		*Athripsodes albifrons*

*: Taxonomies identical to the published study (species level). **: Taxonomies identical to the published study (genus level).

OTU clustering *vs* ASV inference

There is an ongoing discussion about whether ASVs exceed OTUs. The strongest argument to this end is that ASVs are real biological sequences. Hence, they can be compared between different studies in a straightforward way; considered as consistent labels. In comparison, *de novo* OTUs are constructed, or

“clustered”, with respect to the emergent features of each specific dataset. Therefore, OTUs defined in two different data sets cannot be directly compared.

However, the OTU concept is not compulsory related to the clustering approach; it is widely used to describe results based on its biological meaning but it does not imply clustering. In addition, according to *Callahan* et al. [15] “ASV methods infer the biological sequences in the sample prior to the introduction of amplification and sequencing errors, and distinguish sequence variants differing by as little as one nucleotide”. As a result, ASVs could be considered as OTUs of higher resolution.

It is due to this concept confusion that algorithms whose rationale is considerably closer to the variant-based approach, are still considered as OTU clustering algorithms [15]. Swarm v2 produces all possible “microvariants” of an amplicon to implement an exact-string comparison [19]. Furthermore, real biological sequences, “clouds of microvariants”, are produced as its output, which can be used for comparisons between different studies. Thus, Swarm v2 can be considered as an ASV inferring algorithm.

Traditional clustering methods have certain limitations such as arbitrary global clustering thresholds, centroid selection, as it depends on the input order, time-consuming etc [52], that variant-based approaches manage to address. However certain algorithms for OTU clustering as VSEARCH have been proven to be especially reliable and they are widely used by a great number of researchers. Furthermore, ASVs intend to improve taxonomic resolution; however, a vast number of inferred ASVs [53] can lead to inflation of diversity estimates, especially in the case of microbial communities, thus making the analysis even more complicated.

ASV or OTU approaches are supported by PEMA, though we support that similar ecological results are produced by both these methods, as also suggested by *Glassman* et al. [54].

Beyond environmental ecology, ongoing and future work

PEMA is mainly intended to support eDNA metabarcoding analysis and be directly applicable to next-generation biodiversity/ecological assessment studies. Given that community composition analysis may also serve additional research fields, eg. microbial pathology, the potential impact of such pipelines is expected to be much higher. On-going PEMA work focuses on serving a wide scientific audience and on

making it applicable to more types of studies. The easy set up and execution of PEMA, allows users to work closely with national and European HPC/e-infrastructures (e.g. ELIXIR Greece [55], LifeWatch ERIC [56], EMBRC ERIC [57]). To that end and in a mid-term perspective, a Common Workflow Language (CWL) version of PEMA will be explored. The aim of this effort is to reach out to a wider scientific audience and address both their ongoing as well as future analysis needs.

By supporting the analysis of the most commonly used marker genes for Bacteria and Archaea (16S rRNA), Fungi (ITS) and Metazoa (COI/18S rRNA), a holistic biodiversity assessment approach is now possible through PEMA and eDNA metabarcoding; Though, in a mid term perspective, it is our intention to allow *ad hoc* and in-house databases to be used as reference for the taxonomy assignment.

Conclusions

PEMA is an accurate, execution friendly and fast pipeline for eDNA metabarcoding analysis. It provides a per-sample analysis output, different taxonomy assignment methods and graphics-based biodiversity/ecological analysis. This way, in addition to (M)OTU/ASV calling, it provides users with both an informative study overview and detailed result snapshots.

Thanks to a nominal number of installation and execution commands required for PEMA to be set and run, it is considered essentially user friendly. In addition, PEMA's strategic choice of a single parameter file, implementation programming language, and multiple container-type distribution, grant it with speed (running in parallel), on-demand partial pipeline enactment, and provision for HPC-system-based sharing. All the aforementioned features, render PEMA attractive for biodiversity/ecological assessment analyses. By supporting the analysis of the most commonly used marker genes for Prokaryotes (Bacteria and Archaea), as well as Eukaryotes (Fungi and Metazoa), PEMA allows assessment of biodiversity in different levels of biodiversity. Applications may mainly concern environmental ecology with possible extensions to fields like microbial pathology and gut microbiome, inline with modern research needs, from low volume to big data.

Availability of supporting source code and requirements

Project name: PEMA

Project home page: https://github.com/hariszaf/pema

Dockerized version: https://hub.docker.com/r/hariszaf/pema.

Singularity image: https://singularity-hub.org/collections/2295.

Operating system(s): Platform independent

Programming language: BigDataScript

Other requirements: Singularity (in case of HPC usage)

License: GNU GPLv3. For 3rd party components separate licenses apply. See Additional File 1 for a list of tools invoked by PEMA and their respective licenses.

bio.tools id: PEMA

RRID: SCR_017676

Availability of supporting data

The sequence data that support the findings of this study, with respect to the mock-community-based evaluation, are available in European Nucleotide Archive (ENA) with the following study accession numbers - for the 16S, 18S rRNA, ITS and COI marker genes respectively:

PRJNA305443 (https://www.ebi.ac.uk/ena/browser/view/PRJNA305443),

PRJNA314977 (https://www.ebi.ac.uk/ena/browser/view/PRJNA314977),

PRJNA377530 (https://www.ebi.ac.uk/ena/browser/view/PRJNA377530) and

PRJEB23036 (https://www.ebi.ac.uk/ena/browser/view/PRJEB23036)

The real datasets used are also available in ENA:

PRJEB20211 (http://www.ebi.ac.uk/ena/data/view/PRJEB20211) and

PRJEB13009 (https://www.ebi.ac.uk/ena/data/view/PRJEB13009).

An archived version of the code and supporting data is also available via the *GigaScience* database GigaDB [58].

Declarations

List of abbreviations

BDS: BigDataScript

COI: Cytochrome Oxidase Subunit 1

eDNA: Environmental DNA

MOTU: Molecular Operational Taxonomic Unit (used for Eukaryotes)

HPC: High Performance Computing

MCMC: Markov chain Monte Carlo

MSA: Multiple Sequence Alignment

OTU: Operational Taxonomic Unit (used for prokaryotes)

PEMA: a Pipeline for Environmental DNA Metabarcoding Analysis

SSU: Small Subunit

CWL: Common Workflow Language

Ethics approval and consent to participate

Not applicable

Consent for publication

Not applicable

Competing interests

The authors declare that they have no competing interests

Funding

This project has received funding from the Hellenic Foundation for Research and Innovation (HFRI) and the General Secretariat for Research and Technology (GSRT), under grant agreement No 241 (PREGO project). There was no additional external funding received for this study. The funders had no role in study

design, data collection and analysis, decision to publish, or preparation of the manuscript.

Authors' contributions

HZ conceived and designed the pipeline, performed its containerization, analyzed and interpreted the data, wrote the paper, prepared figures and/or tables, reviewed drafts of the paper. HQV offered support in the HPC preparation and setup and in 3rd party component usage. KV and CA conceived the idea and reviewed drafts of the paper. PT conceived the idea, proposed the usage of the programming language and reviewed drafts of the paper. CP conceived the idea, prepared figures and/or table and reviewed drafts of the paper. AP offered support in HPC and in 3rd party components. EP conceived the idea, assisted with programming and setup and reviewed drafts of the paper. All authors read and approved the final manuscript.

Acknowledgements

The authors would like to thank: a. the Information technology (IT) group of HCMR and especially Mr Stelios Ninidakis, Mr Georgios Tsamis and Mr Dimitris Sidirokastritis for their help and support during cluster maintenance and installation of third party software. b. Dr. Christos A. Christakis (ORCID iD: 0000-0002-7075-0996) for his valuable feedback on ecological analysis usefulness aspects.

This research was supported in part through computational resources provided by IMBBC (Institute of Marine Biology, Biotechnology and Aquaculture) of the HCMR (Hellenic Centre for Marine Research). Funding for establishing the IMBBC HPC has been received by the MARBIGEN (EU Regpot) project, LifeWatchGreece RI and the CMBR (Centre for the study and sustainable exploitation of Marine Biological Resources) RI.

References

[1] Pavan-Kumar A, Gireesh-Babu P, Lakra WS. DNA metabarcoding: a new approach for rapid biodiversity assessment. J Cell Sci Mol Biol. 2015;2(1):111.

[2] Thomsen PF and Willerslev E. Environmental dna–an emerging tool in conservation for monitoring

past and present biodiversity. Biological Conservation. 2015; 183:.4–18.

[3] Ji Y, Ashton L, Pedley SM, Edwards DP, Tang Y, Nakamura A, Kitching R, Dolman PM, Woodcock P, Edwards FA, Larsen TH. Reliable, verifiable and efficient monitoring of biodiversity via metabarcoding. Ecology letters. 2013 Oct;16(10):1245-57.

[4] Schloss PD, Westcott SL, Ryabin T, Hall JR, Hartmann M, Hollister EB, et al. Introducing mothur: open-source, platform-independent, community-supported software for describing and comparing microbial communities. Appl. Environ. Microbiol. 2009; 75:7537-41.

[5] Bolyen E, Rideout JR, Dillon MR, Bokulich NA, Abnet C, Al-Ghalith GA, et al. QIIME 2: Reproducible, interactive, scalable, and extensible microbiome data science. PeerJ Preprints. 2018; 6:e27295v2.

[6] Hildebrand F, Tadeo R, Voigt AY, Bork P, Raes J. LotuS: an efficient and user-friendly OTU processing pipeline. Microbiome. 2014; 2:30.

[7] Normandeau E. Environmental DNA metabarcoding analysis. https://github.com/enormandeau/barque. Accessed 10 November 2019.

[8] Axtner J, Crampton-Platt A, Hoerig LA, Mohamed A, Xu CC, Yu DW, Wilting A. An efficient and robust laboratory workflow and tetrapod database for larger scale environmental DNA studies. GigaScience. 2019 Apr 13;8(4):giz029.

[9] Gweon HS, Oliver A, Taylor J, Booth T, Gibbs M, Read DS, Griffiths RI, Schonrogge K. PIPITS: an automated pipeline for analyses of fungal internal transcribed spacer sequences from the I llumina sequencing platform. Methods in Ecology and Evolution. 2015 Aug;6(8):973-80.

[10] European Strategy Forum on Research Infrastructures Innovation Working Group. Innovation-oriented cooperation of Research Infrastructures. Vol.3. ESFRI Scripta. 2018.

[11] Cingolani P, Sladek R, Blanchette M. BigDataScript: a scripting language for data pipelines. Bioinformatics. 2014; 31:10-16.

[12] Rad BB, Bhatti HJ, Ahmadi M. An introduction to docker and analysis of its performance. International Journal of Computer Science and Network Security (IJCSNS). 2017; 17:228.

[13] Kurtzer GM, Sochat V, Bauer MW. Singularity: Scientific containers for mobility of compute. PloS

one. 2017; 12:e0177459.

[14] Coissac E, Riaz T, Puillandre N. Bioinformatic challenges for DNA metabarcoding of plants and animals. Molecular ecology. 2012 Apr;21(8):1834-47.

[15] Callahan BJ, McMurdie PJ, Holmes SP. Exact sequence variants should replace operational taxonomic units in marker-gene data analysis. The ISME journal. 2017 Dec;11(12):2639.

[16] Pauvert C, Buée M, Laval V, Edel-Hermann V, Fauchery L, Gautier A, Lesur I, Vallance J, Vacher C. Bioinformatics matters: the accuracy of plant and soil fungal community data is highly dependent on the metabarcoding pipeline. Fungal Ecology. 2019 Oct 1;41:23-33.

[17] Rognes T, Flouri T, Nichols B, Quince C, Mahé F. VSEARCH: a versatile open source tool for metagenomics. PeerJ. 2016; 4:e2584.

[18] Hao X, Jiang R, Chen T. Clustering 16S rRNA for OTU prediction: a method of unsupervised Bayesian clustering. Bioinformatics. 2011; 27:611-8.

[19] Mahé F, Rognes T, Quince C, de Vargas C, Dunthorn M. Swarm v2: highly-scalable and high-resolution amplicon clustering. PeerJ. 2015; 3:e1420.

[20] Lanzén A, Jørgensen SL, Huson DH, Gorfer M, Grindhaug SH, Jonassen I, et al. CREST–classification resources for environmental sequence tags. PloS one. 2012; 7:e49334.

[21] Quast C, Pruesse E, Yilmaz P, Gerken J, Schweer T, Yarza P, Peplies J, Glöckner FO. The SILVA ribosomal RNA gene database project: improved data processing and web-based tools. Nucl. Acids Res. 2013; 41:D590-6.

[22] Nilsson RH, Larsson KH, Taylor AF, Bengtsson-Palme J, Jeppesen TS, Schigel D, Kennedy P, Picard K, Glöckner FO, Tedersoo L, Saar I. The UNITE database for molecular identification of fungi: handling dark taxa and parallel taxonomic classifications. Nucleic acids research. 2018 Oct 29;47(D1):D259-64.

[23] Kozlov AM, Darriba D, Flouri T, Morel B, Stamatakis A. RAxML-NG: a fast, scalable and user-friendly tool for maximum likelihood phylogenetic inference. Bioinformatics. 2019; btz305.

[24] Barbera P, Kozlov AM, Czech L, Morel B, Darriba D, Flouri T, Stamatakis A. EPA-ng: massively parallel evolutionary placement of genetic sequences. Systematic biology. 2018; 68:365-9.

[25] Wang Q, Garrity GM, Tiedje JM, Cole JR. Naive Bayesian classifier for rapid assignment of rRNA

sequences into the new bacterial taxonomy. Appl. Environ. Microbiol. 2007; 73:5261-7.

[26] Machida RJ, Leray M, Ho SL, Knowlton N. Metazoan mitochondrial gene sequence reference datasets for taxonomic assignment of environmental samples. Scientific data. 2017; 4:170027.

[27] McMurdie JP, Holmes S. phyloseq: an R package for reproducible interactive analysis and graphics of microbiome census data. PloS one. 2013; 8:e61217.

[28] Andrews S. FastQC. http://www.bioinformatics.babraham.ac.uk/projects/fastqc/. Accessed 08 July 2019.

[29] Bolger AM, Lohse M, Usadel B. Trimmomatic: a flexible trimmer for illumina sequence data. Bioinformatics. 2014; 30:2114-20.

[30] Martin M. Cutadapt removes adapter sequences from high-throughput sequencing reads. EMBnet. journal. 2011 May 2;17(1):10-2.

[31] Nikolenko SI, Korobeynikov AI, Alekseyev MA. Bayeshammer: Bayesian clustering for error correction in single-cell sequencing. BMC genomics. 2013; S7.

[32] Bankevich A, Nurk S, Antipov D, Gurevich AA, Dvorkin M, Kulikov AS, et al. Spades: a new genome assembly algorithm and its applications to single-cell sequencing. Journal of computational biology. 2012; 19:455-77.

[33] Masella AP, Bartram AK, Truszkowski JM, Brown DG, Neufeld JD. PANDAseq: paired-end assembler for illumina sequences. BMC bioinformatics. 2012; 13:31.

[34] Boyer F, Mercier C, Bonin A, Le Bras Y, Taberlet P, Coissac E. OBITools: a unix-inspired software package for dna metabarcoding. Molecular ecology resources. 2016; 16:176-82.

[35] Edgar RC. Search and clustering orders of magnitude faster than BLAST. Bioinformatics. 2010 Aug 12;26(19):2460-1.

[36] Benson DA, Cavanaugh M, Clark K, Karsch-Mizrachi I, Ostell J, Pruitt KD, Sayers EW.GenBank. Nucleic acids research 2018; 46:D41-47.

[37] Czech L, Barbera P, Stamatakis A. Methods for automatic reference trees and multilevel phylogenetic placement. Bioinformatics. 2018; 35:1151-8.

[38] Berger SA, Stamatakis A. PaPaRa 2.0: a vectorized algorithm for probabilistic phylogeny-aware

alignment extension. Heidelberg Institute for Theoretical Studies. 2012.

[39] Letunic I, Bork P. Interactive tree of life (itol): an online tool for phylogenetic tree display and annotation. Bioinformatics. 2006; 23:127-8.

[40] Katoh K, Misawa K, Kuma KI, Miyata T. Mafft: a novel method for rapid multiple sequence alignment based on fast fourier transform. Nucleic acids research. 2002; 30:3059-66.

[41] Chavez J. Singularity: a "Docker" for HPC environments. https://dev.to/grokcode/singularity--a-docker-for-hpc-environments-i6p. Accessed 08 Jul 2019.

[42] Gohl DM, Vangay P, Garbe J, MacLean A, Hauge A, Becker A, Gould TJ, Clayton JB, Johnson TJ, Hunter R, Knights D. Systematic improvement of amplicon marker gene methods for increased accuracy in microbiome studies. Nature biotechnology. 2016 Sep;34(9):942.

[43] Bradley IM, Pinto AJ, Guest JS. Design and evaluation of Illumina MiSeq-compatible, 18S rRNA gene-specific primers for improved characterization of mixed phototrophic communities. Appl. Environ. Microbiol.. 2016 Oct 1;82(19):5878-91.

[44] Bakker MG. A fungal mock community control for amplicon sequencing experiments. Molecular ecology resources. 2018 May;18(3):541-56.

[45] Bista I, Carvalho GR, Tang M, Walsh K, Zhou X, Hajibabaei M, Shokralla S, Seymour M, Bradley D, Liu S, Christmas M. Performance of amplicon and shotgun sequencing for accurate biomass estimation in invertebrate community samples. Molecular ecology resources. 2018 Sep;18(5):1020-34.

[46] Pavloudi C, Kristoffersen JB, Oulas A, De Troch M, Arvanitidis C. Sediment microbial taxonomic and functional diversity in a natural salinity gradient challenge Remane's "species minimum" concept. PeerJ. 2017; 5:e3687.

[47] Bista I, Carvalho GR, Walsh K, Seymour M, Hajibabaei M, Lallias D, et al. Annual time-series analysis of aqueous edna reveals ecologically relevant dynamics of lake ecosystem biodiversity. Nature communications. 2017; 8:14087.

[48] Harrison PW, Alako B, Amid C, Cerdeño-Tárraga A, Cleland I, Holt S, et al. The European Nucleotide Archive in 2018. Nucleic acids research. 2018; 47:D84-8.

[49] Ting K.M. Precision and Recall. In: Sammut C., Webb G.I. (eds) Encyclopedia of Machine Learning.

Springer, Boston, MA. 2011.

[50] Camacho C, Coulouris G, Avagyan V, Ma N, Papadopoulos J, Bealer K, Madden TL BLAST+: architecture and applications. BMC bioinformatics. 2009; 10:421.

[51] Ratnasingham S, Hebert PD. BOLD: The Barcode of Life Data System (http://www. barcodinglife. org). Molecular ecology notes. 2007 May;7(3):355-64.

[52] Mahé F, Rognes T, Quince C, de Vargas C, Dunthorn M. Swarm: robust and fast clustering method for amplicon-based studies. PeerJ. 2014 Sep 25;2:e593.

[53] Lumping versus splitting – is it time for microbial ecologists to abandon OTUs? http://fiererlab.org/2017/05/02/lumping-versus-splitting-is-it-time-for-microbial-ecologists-to-abandon-otus/. Accessed 20 December 2019.

[54] Glassman SI, Martiny JB. Broadscale ecological patterns are robust to use of exact sequence variants versus operational taxonomic units. MSphere. 2018 Aug 29;3(4):e00148-18.

[55] ELIXIR-GR. https://www.elixir-greece.org/. Accessed 08 July 2019.

[56] LifeWatch-ERIC. https://www.lifewatch.eu/. Accessed 08 July 2019.

[57] EMBRC. http://www.embrc.eu/. Accessed 08 July 2019.

[58] Zafeiropoulos H, Quoc VH, Vasileiadou K, Potirakis A, Arvanitidis C, Topalis P, Pavloudi C, Pafilis E. Supporting data for "PEMA: a flexible Pipeline for Environmental DNA Metabarcoding Analysis of the 16S/18S rRNA, ITS and COI marker genes" GigaScience Database 2020. http://dx.doi.org/10.5524/100715

Figure legends

Figure 1: PEMA comprises four parts. The first step (top left) is the quality control and pre-processing of the Illumina sequencing reads. This step is common for both 16S rRNA and COI marker genes. The second step (top right) is the clustering of reads to (M)OTUs or their inferring to ASVs. The third step (bottom left) is the taxonomy assignment to the generated (M)OTUs/ASVs. In the fourth step (bottom right), the results of the metabarcoding analysis are provided to the user and visualized.

Figure 2: Phylogeny-based taxonomy assignment. A: Building a reference tree for the phylogeny-based taxonomy assignment to 16S rRNA marker gene OTUs: from the latest edition of Silva SSU, all entries referring to Bacteria and Archaea were used and using "art" algorithm, 10000 consensus taxa were kept. B: Using PaPaRa and the OTUs that come up from every analysis, an MSA was made and EPA-ng took over the phylogeny based taxonomy assignment.

Figure 3: OTUs bar plot at the Phylum level. Bar plot depicting the taxonomy of the retrieved OTUs from PEMA for *Pavloudi*'s et al. [46] dataset, at the Phylum level for the case of the 16S marker gene.

Additional files

Additional file 1: Supplementary Methods: Description of tools invoked by PEMA and their licences. Description of the commands, along with their parameters, used to run PEMA, mothur, LotuS and QIIME 2.

Additional file 2: Mock Communities: Details about the mock communities chosen and their corresponding studies as well as the returned output of PEMA for each of those for a number of sets of parameters.

Additional file 3: Table S1: Number of sequences after each pre-processing step for the case of 16S rRNA gene.

Additional file 4: Table S2: Diversity indices of the samples.

Additional file 5: Figure S1: Linear regression between the number of OTUs (averaged per sampling station) and the salinity of the sampling stations. L: Lagoon. S: Sea. R: River. AR: Arachthos. ARO: Arachthos Neochori. ARDelta: Arachthos Delta. LOin: Logarou station inside the lagoon. LOout: Logarou station in the channel connecting the lagoon to the gulf. Kal: Kalamitsi.

Additional file 6: Figure S2: Bar plot depicting the taxonomy of the retrieved OTUs from LotuS at the Phylum level.

Additional file 7: Figure S3: Bar plot depicting the taxonomy of the retrieved OTUs from QIIME 2 using Deblur at the Phylum level.

Additional file 8: Figure S4: Bar plot depicting the taxonomy of the retrieved OTUs from QIIME 2 using DADA2 at the Phylum level.

Additional file 9: Figure S5: Bar plot depicting the taxonomy of the retrieved OTUs from LotuS at the class of Betaproteobacteriales.

Additional file 10: Figure S6: Bar plot depicting the taxonomy of the retrieved OTUs from QIIME 2 using Deblur at the class of Betaproteobacteriales.

Additional file 11: Figure S7: Bar plot depicting the taxonomy of the retrieved OTUs from PEMA at the class of Betaproteobacteriales.

Additional file 12: Table S3: Number of sequences after each pre-processing step for the case of COI,

dataset from *Bista* et al. [47].

Figure 1

Click here to access/download;Figure;Figure 1.png

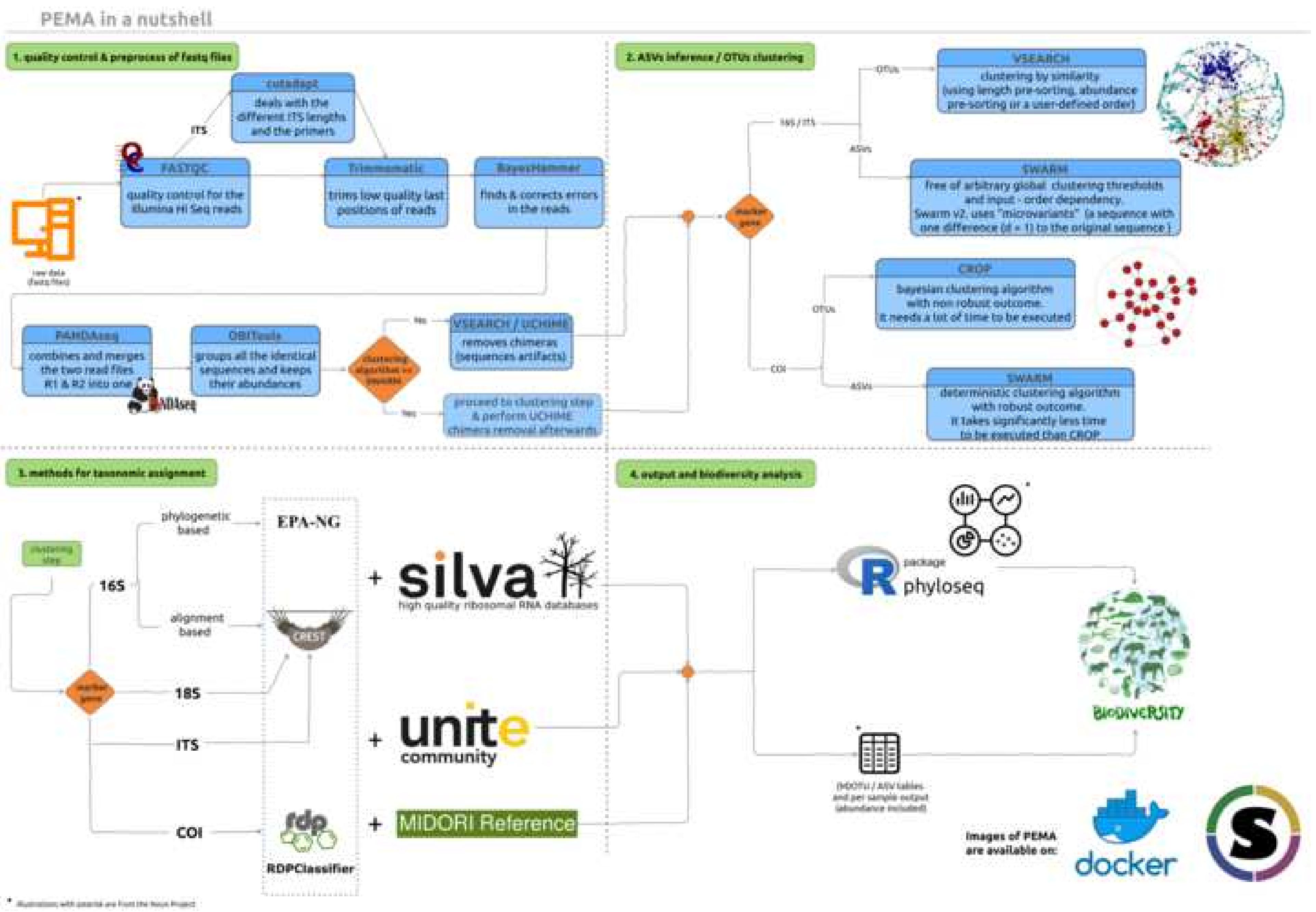

Figure 2 Click here to access/download;Figure;Figure 2.png

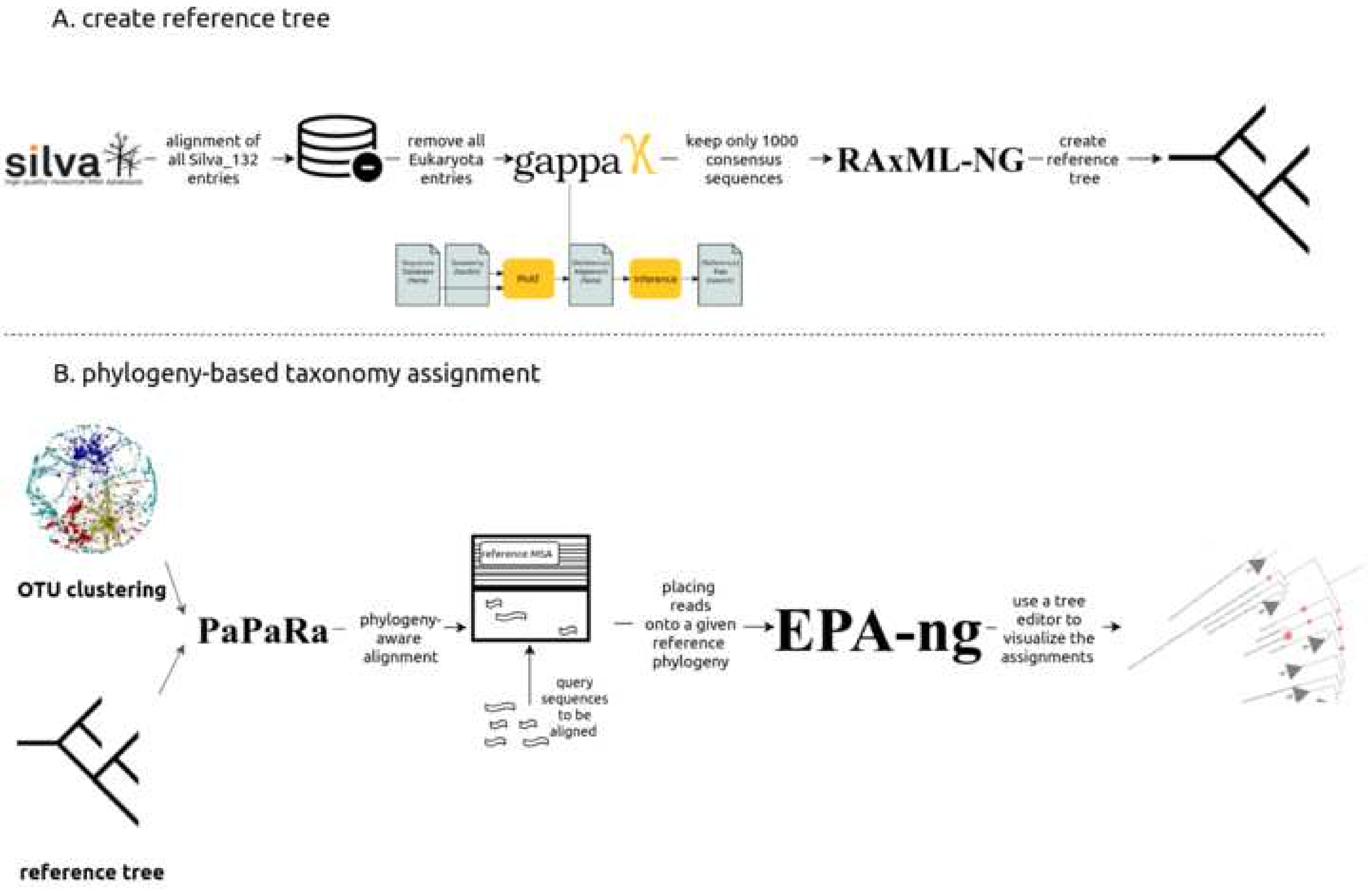

Figure 3

Click here to access/download;Figure;Figure 3.png

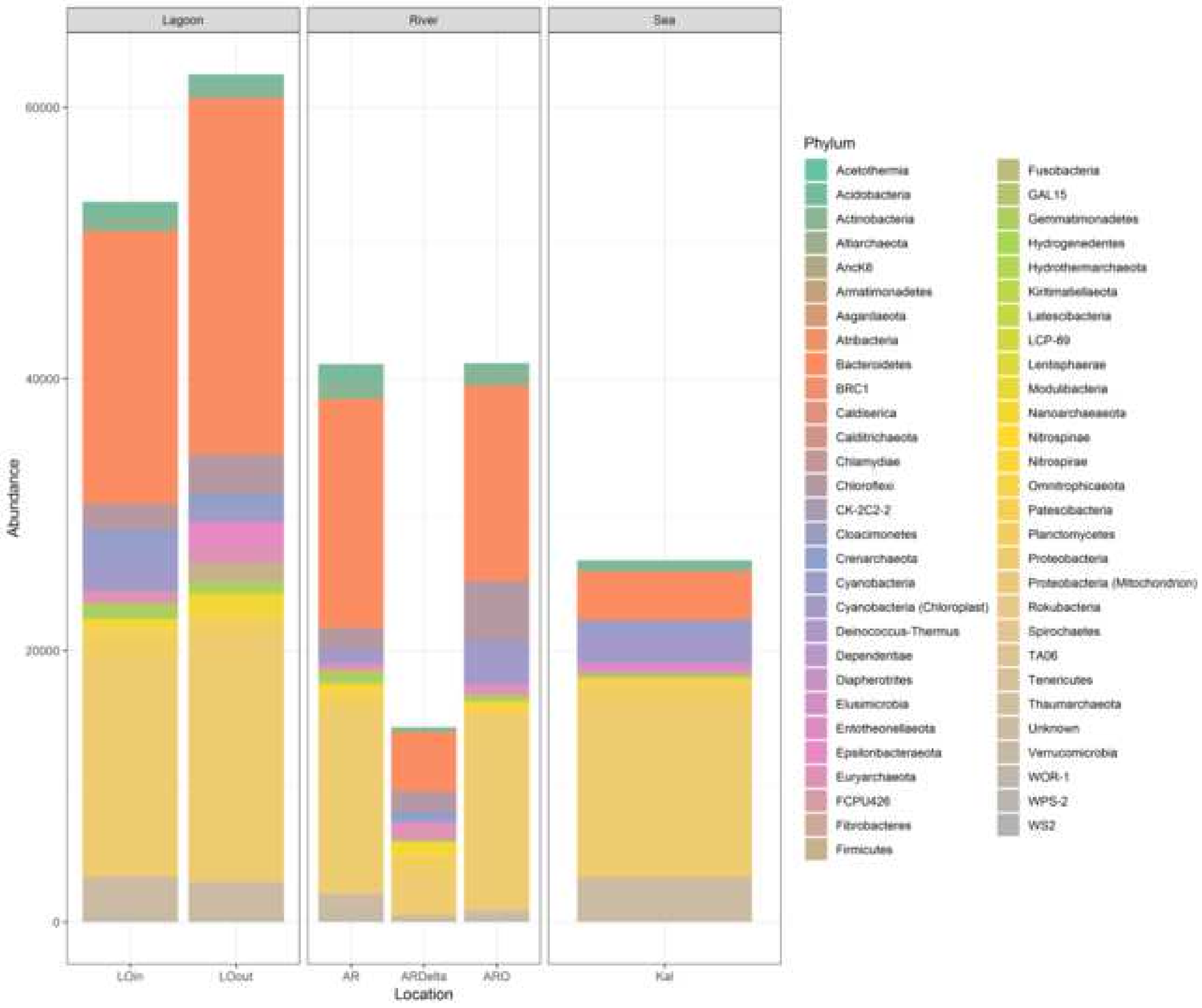

Additional file 1 : Supplementary methods

Click here to access/download
Supplementary Material
Additional file 1_ Supplementary Methods.docx

Additional file 2 : Mock communities

Click here to access/download
Supplementary Material
Additional file 2_ Mock Communities .xlsx

Additional file 3 : Table S1

Click here to access/download
Supplementary Material
Additional file 3_Table S1.docx

Additional file 4 : Table S2

Click here to access/download
Supplementary Material
Additional file 4_Table S2.docx

Additional file 5 : Figure S1

Click here to access/download
Supplementary Material
Additional file 5_ Figure S1.png

Additional file 6 : Figure S2

Click here to access/download
Supplementary Material
Additional file 6_Figure S2.png

Additional file 7 : Figure S3

Click here to access/download
Supplementary Material
Additional file 7_Figure S3.png

Additional file 8 : Figure S4

Click here to access/download
Supplementary Material
Additional file 8_Figure S4.png

Additional file 9 : Figure S5

Click here to access/download
Supplementary Material
Additional file 9_Figure S5.png

Additional file 10 : Figure S6

Click here to access/download
Supplementary Material
Additional file 10_Figure S6.png

Additional file 11 : Figure S7

Click here to access/download
Supplementary Material
Additional file 11_Figure S7.png

Additional file 12 : Table S3

Click here to access/download
Supplementary Material
Additional file 12_ Table S3.docx

Reviews of a previous version

Click here to access/download
Supplementary Material
PEMA_Reviews_PreviousSubmission.odt

Cover letter & point-by-point response to the reviewer's comments Click here to access/download;Personal Cover;Cover letter & response to the reviewers comments.docx

hcmr
ΕΛΚΕΘΕ

Dr. Hans Zauner
Editor in GigaScience journal
BGI Hong Kong Ltd.

Heraklion, 4th January, 2020

Dear Dr. Zauner,

Please find enclosed our revised manuscript "***PEMA: a flexible Pipeline for Environmental DNA Metabarcoding Analysis of the 16S/18S rRNA, ITS and COI marker genes***" by H. Zafeiropoulos, H. Q. Viet, K. Vasileiadou, A. Potirakis, C. Arvanitidis, P. Topalis, C. Pavloudi and E. Pafilis, which we would like to submit for publication in the GigaScience journal, as a Technical Note.

We believe our findings would appeal to the readership of GigaScience journal. The article presents PEMA, a Pipeline for Environmental DNA Metabarcoding Analysis, which is a containerized assembly of key metabarcoding analysis tools with a significantly low effort in setting up, running and customizing to researchers' needs. PEMA is based on third party tools and performs the processing of Illumina reads, their clustering to (M)OTUs or their tinferring of ASVs and their taxonomic assignment.

PEMA allows a container-based installation via Docker and Singularity; the latter is recommended for HPC environments. PEMA attempts to address the metabarcoding analysis of big datasets coming from a variety of systems (e.g. environmental samples, gut microbiome etc.). Furthermore, by being time-efficient and allowing on-demand partial execution, PEMA enhances the efforts for benchmarking of metabarcoding analyses.

The manuscript is a revision of the GIGA-D-19-00397 manuscript.

In this revised version of our manuscript, all the points raised by the reviewers have been addressed. A detailed point-by-point response to the reviewers' comments is attached.

We propose that the manuscript is forwarded to the initial (GIGA-D-19-00261) submission reviewers:

- Dr. Johan Andre Pansu (johanandre.pansu@mq.edu.au)
- Dr. Charles Xu (cong.xu3@mail.mcgill.ca)

We confirm that this manuscript has not been published elsewhere and is not under consideration by another journal, and that all authors have approved the manuscript and agree with its submission to the GigaScience journal. None of the authors express any conflict of interest.

Please address all correspondence to:

Charalampos (Haris) Zafeiropoulos
Hellenic Centre for Marine Research
Thalassocosmos, P.O. Box 2214
71003 Heraklion, Crete, Greece
email: haris-zaf@hcmr.gr
Tel.: +30 2810 337740

We look forward to hearing from you at your earliest convenience.

Yours sincerely,

Haris Zafeiropoulos (on behalf of the authors)

Ελληνικό Κέντρο Θαλασσίων Ερευνών, Γούρνες Πεδιάδος, Τ.Θ. 2214, 71003 Ηράκλειο Κρήτης, Ελλάδα
Hellenic Centre For Marine Research, Gournes Pediados, P.O. Box 2214, 71003 Heraklion, Crete, Greece
Τηλ/Tel: + 30 2810 337806, Fax: + 30 2810 337822, URL: www.hcmr.gr

Response to the editor's and reviewer's comments

We would like to kindly thank the reviewer for the time he spent to thoroughly read our manuscript. We sincerely appreciate all accurate comments made by the reviewer, which helped the improvement of our manuscript. In the revised version, we have addressed all of the reviewer's comments and suggestions and where necessary we have incorporated changes and made amendments and alterations to the manuscript. The changes and the new sections of the manuscript are written in blue. Below we cite our detailed answers (in blue) to the editor and reviewers' comments and suggestions (italic).

Editor's comment #1
In particular, the section on comparing the tool with existing pipelines needs more attention with respect to the way this comparison is presented in the manuscript. As the reviewer says, "PEMA allows to switch from one (existing) tool to another at each step of the filtering process, therefore differences are the result of the combination of tools decided by the user, not of PEMA itself."
Switching tools without having to re-run an analysis from scratch, is one of PEMA's main advantages. Providing the user the ability for a great number of tests, while tuning numerous parameters of the tools selected, PEMA allows for a thorough benchmarking for each and every study. Regarding the way that the comparison between PEMA and other metabarcoding pipelines is presented, please see Editor's comment #2 and Reviewer's comment #2.

Editor's comment #2
I do feel that comparisons to similar tools are an important aspect for our Technical Notes, but please make sure that the reader is not getting potentially misleading impressions.
A paragraph explaining both the purpose and the limitations of comparing pipelines has been added as a "preface" in the "*Evaluation on real datasets and against other tools*" section (lines 345-352 of the revised version of the manuscript). Certain changes have been made in the manuscript to clarify any potential misconception regarding this issue (e.g. lines 389-390).

Editor's comment #3
If you have not yet done so, please register your new software application in the bio.tools and SciCrunch.org databases to receive RRID (Research Resource Identification Initiative ID) and biotoolsID identifiers, and include these in your manuscript. This will facilitate tracking, reproducibility and re-use of your tool.
PEMA was not registered in bio.tools or SciCrunc.org databases until the last submission. Now it has been registered in both and has the unique ids: "PEMA" (in biotools) and "SCR_017676" (RRID in SciCrunch.org). They have both been included in the revised PEMA manuscript.

Reviewer's comment #1
I have reviewed for the second time the manuscript from Zafeiropoulos et al. "PEMA: a flexible Pipeline for Environmental DNA Metabarcoding Analysis of the 16S/18S rRNA, ITS and COI marker genes". First, I would like to acknowledge the major improvements implemented by the authors in their pipeline since the first submission, namely: the extension of the functionality to two other marker genes (18S rRNA and ITS) and the inclusion of tools for inferring ASV. This is undoubtedly a great set of additional tools, and the pipeline now covers four of the most common markers for eDNA studies (but not all marker genes as mentioned in the responses). That said, attending that similar pipelines already exist (see PipeCraft for another example; Anslan et al. 2017 Molecular Ecology), I think the main strength of this pipeline is in flexibility, time efficiency and ability to handle large datasets.
Thank you for your patience for a second review. It is the most appreciated.
With respect to PipeCraft, unfortunately we did not achieve to test it. In their paper, Anslan et al (2017) write that it is available through PlutoF system (download link https://plutof.ut.ee/#/datacite/10.15156%2FBIO%2F587450), however the link does not work. In addition, PipeCraft is not registered in any other repository, making it impossible for end-users to reach it.

Reviewer's comment #2
I appreciate the inclusion of mock community analyses that, contrarily to real eDNA dataset, allow the reader to have

an idea of what to expect and easily compare outputs of the pipeline with the biological reality (although this section could gain in readability by avoiding study-specific details). However, I am still very puzzled by the comparisons with other software. I agree with the authors when they say that "it is important to assess the variability that different tools introduce in the produced outputs of each study", and PEMA allows that by offering different options at every step of the pipeline, but it seems abusive to say that results from PEMA outperforms those of other pipelines (e.g. lines 370-372). Comparing pipelines make sense when there is algorithm development but here PEMA allows to switch from one (existing) tool to another at each step of the filtering process, therefore differences are the result of the combination of tools decided by the user, not of PEMA itself.
In my opinion, these comparisons can appear as misleading, and make the paper more complicated and longer than needed.
Regarding the sentence in lines 370-372 (previous version of the manuscript), it has been rephrased (lines 389-390 of the revised version of the manuscript), hopefully not causing misconceptions. In addition, in the paragraph added (preface) in this section, an extended explanation of the rationale of this comparison clarifies this issue (lines 345-352). However, we would like to specify our point-of-view on this issue. The comparison with other software is one of the prerequisite criteria of the journal's author guidelines for "Technical Notes" (https://academic.oup.com/gigascience/pages/technical_note). As mentioned there: "*The tool or method needs to have been tested, and properly compared to any existing tools or methods used by the community. It does not necessarily have to outperform existing approaches, but it should show innovation in the approach, implementation, or have added benefits that have been needed in this arena.*" That is the reason we thought about adding the section "*Evaluation on real datasets and against other tools*" and run these tests in the first place.
According to the reviewer: "*PEMA allows to switch from one (existing) tool to another at each step of the filtering process, therefore differences are the result of the combination of tools decided by the user, not of PEMA itself*". PEMA does not, indeed, contain novel algorithm development. However, being able to select among a richer pool of tools so as to perform a high quality metabarcoding analysis is the very essence of PEMA. PEMA users being able to combine third-party tools (out of a rich set) in an easy way constitutes an *"added benefit that has been needed in this arena"* (based on the GigaScience journal aim mentioned above). It is this easiness along with more of PEMA distinctive features (such as checkpoints and partial workflow re-execution) that justify the flexibility claimed in the manuscript title *"PEMA: a flexible Pipeline for Environmental DNA Metabarcoding".*
By comparing metabarcoding pipelines we cannot argue in a straightforward way that one is better than the other; it is definitely not the aim of this section. By performing these analyses we intend to present the potentials of each of those pipelines as well as their computational needs in each case. More specifically, you cannot say that PEMA is generally faster than any other pipeline; for example, if the CROP algorithm is selected, then for sure the analysis will take a long time. However, a more general view of such analyses does provide the reader with important information and, in all cases, it highlights the importance of its own role when setting the parameters.

Reviewer's comment #3
Finally, I am still not convinced by the usefulness of Figs. 3, 4 and 5 in the main text. This is a technical note and, without any direct comparison, they do not bring much information. I would suggest to either make a synthetic figure or to move them to supplementary material (but there are already many supplementary files).
The main aim of the figures mentioned is to visualize the findings described in the "Evaluation on real datasets and against other tools". We agree with the reviewer's comment that in a technical note they could be avoided and it is our belief that we should remove Figures 3 and 4 at all. However, it is our belief that Figure 5 (Figure 3 in the revised version of the manuscript) should be kept as an example of the visualization that PEMA supports.

Reviewer's comment #4
In further communication, please add line number in the responses to editor/reviewers to indicate where changes have been made.
We have followed the recommendation of the reviewer.

Reviewer's comment #5
Line 74-78: please reformulate, the current definition of metabarcoding is quite vague.
The definition of metabarcoding has been rephrased (lines 76-79 of the revised version of the manuscript).

Reviewer's comment #6
Line 78: it is rather a "potential holistic approach"
The sentence was rephrased (line 81 of the revised version of the manuscript).

Reviewer's comment #7
Line 82-88: for each marker, please explain what taxonomic group(s) it targets. Also, authors could make explicit that

any primer pairs amplifying one of these regions can be used as long as paired-end reads can be merged successfully.
With respect to the taxonomic groups, we have followed the recommendation of the reviewer (see lines 76-79). Regarding the primer pairs, it is our belief that as this is a background paragraph describing the metabarcoding method, it would be better not to include it. That is because metabarcoding studies may occur with single end reads as well.

Reviewer's comment #8
Line 87-88: there are already some pipelines for this.
An example of a pipeline for the ITS marker gene has been added (line 90 of the revised version of the manuscript). The main point of this sentence is to underlie the need for a fast and flexible pipeline for those two marker genes (COI and ITS) too.

Reviewer's comment #9
Line 112-115: this paragraph could place later to increase readability (e.g. after the next paragraph or in the discussion)
It is our belief that this paragraph should remain in its current place in the manuscript (lines 117-120 in the revised version of the manuscript). Its aim is to justify what comes to the exact next paragraph; PEMA supports both OTU clustering and ASV inference because of the fact that "The use of ASVs instead of OTUs has been suggested [14], however the choice for which approach to use should rely on each study's objective(s) [15]." Furthermore, we consider that a definition of the ASVs is needed here.

Reviewer's comment #10
Line 137-138: What about samples with a low number of reads? This could be part of the initial quality check.
We have followed the recommendation of the reviewer and changed the sentence accordingly (lines 144-145).

Reviewer's comment #11
Line 164-165: Is there two chimera removal steps: Vsearch in Part 1 and later step in part 3? Or is it only when using Swarm? Can you please explain.
The chimera removal step occurs only once in all cases, as it is also shown in the figure describing the pipeline (Figure 1). What changes is the order of the steps, depending on the algorithms selected by the user. If the Swarm v2 algorithm has been selected, then the chimera removal takes place after the ASV inference. In all other cases, the chimera removal step occurs before the OTU clustering. We added a sentence at the end of the previous section ("Part 1: Quality control and pre-processing of raw data") to clarify this (lines 153-155).

Reviewer's comment #12
Lines 238-241: Please reformulate
We have followed the recommendation of the reviewer and rephrased the sentence (lines 247-255).

Reviewer's comment #13
Line 249 and additional file 2: The description of the tools and parameters used for each dataset (as well as the rationales for choosing them) would be welcomed here.
As mentioned in the manuscript, the tools and the parameters used in each run can be found in the "Additional file 2: Mock communities". More specifically, a separate sheet for each marker gene can be found in this document, where PEMA's output as long as the corresponding statistics are shown for each tool and parameter set. Regarding the rationales for choosing those mock communities, a paragraph has been added in the "Additional file 2: Mock communities" on the "datasets" sheet.

Reviewer's comment #14
Line 258-317: This section could be reduced by removing too species-specific details (e.g. lines 283-285).
As we share the common belief for a not excessively long manuscript, we removed any species-specific and mock-community-specific details for the manuscript as possible (e.g. lines 283-285 of the previous version of the manuscript). However, as it is of great importance to distinguish PEMA's false positives and true negatives from those that occur due to the datasets' features we kept such details when considered that is needed to that end.

Reviewer's comment #15
Line 270-273: This paragraph should be moved elsewhere as it is valid for all markers.
We have followed the recommendation of the reviewer. The paragraph has been moved at the end of the previous section (lines 272-276).

Reviewer's comment #16

Lines 311-314: unclear, please reformulate.
We have followed the recommendation of the reviewer and reformulated the sentence (lines 322-327).

Reviewer's comment #17
Lines 429-440: References would be welcomed here.
We have followed the recommendation of the reviewer (lines 446-458).

Reviewer's comment #18
Line 437-440: Please explain more in details what you mean here. I am not sure I fully agree with this statement.
We have followed the recommendation of the reviewer (lines 455-458). The point of this paragraph is to discuss that by making use of ASVs, especially in the case of microbial communities, we might end up with a vast number of sequences, both because of high alpha- (intra-sample variation) and beta (inter-sample variation) diversity. This complicates even more downstream statistical analyses and the drawing of conclusions about the dynamics of the communities under study.

Reviewer's comment #19
Table 5: This table does not seem necessary now that authors added mock community analyses, especially if the original community is unknown. It seems redundant.
The aim of this study is to show PEMA's performance from a biological point-of-view in real data, compared to those of Barque. As mentioned in the table's label, the comparison is against the initial study's positive controls. Thus, the output of two different pipelines can be compared as the composition of those samples is known. For the case of the *Pavloudi* et al. dataset, there is no such positive controls and that is why there is not an equivalent table presented.

Reviewer's comment #20
Table S2 (previously Table 5): I still do not understand what the authors mean by "N = total microbial relative abundance". It seems to me that these numbers represent the number of reads? If so, the term "relative abundance" is inappropriate and confusing (one would expect a percentage).
High throughput sequencing can only provide relative estimates of abundances. Absolute abundances can be provided by other methods, such as quantitative PCR (Q-PCR). Therefore, it is well established that high throughput sequencing can only result in relative abundances of taxa.
"N" has been used extensively among biodiversity scientists to denote the "total number of individuals" of a given taxon, therefore its "total abundance" (for example, see: 10.1016/S0022-0981(98)00028-8). Thus, in our case, as well as in other studies where it has been used to describe metabarcoding data, it denotes the "total microbial relative abundance values" (see: 10.7717/peerj.3687).

For example, consider the example below, of a classic abundance table (taxa per stations):

	Station A	Station B
Taxon 1	15	22
Taxon 2	0	52
Taxon 3	2	34

In this example case, when one would calculate the classic biodiversity indices, the result would be the following:

	S (number of taxa)	N (total abundance)
Station A	2	17
Station B	3	108
SUM	5	125

If these indices were to be presented as percentages, the result would be:

	Percentage of each station’s number of taxa to the sum of the number of taxa found in all the stations of the study	Percentage of each station’s total abundance of taxa to the sum of the total abundances of taxa found in all the stations of the study
Station A	40 %	13.6 %
Station B	60 %	86.4 %

The logic is the same, however we cannot use the term “total abundance”. These numbers do not just represent the number of reads, since the reads have undergone all the necessary processing (which is thoroughly explained in the manuscript) in order to derive to the final (M)OTU/ASV table (the equivalent of the classic abundance table in the case of eDNA metabarcoding). The number of sequences, i.e. reads) after each pre-processing step are shown in Additional file 3: Table S1.
