# Supplementary material for: PEMA: a flexible Pipeline for Environmental DNA Metabarcoding Analysis of the 16S/18S ribosomal RNA, ITS, and COI marker genes
Source: Gigascience. 2020 Mar 12;9(3):giaa022. doi: 10.1093/gigascience/giaa022 (PMC7066391; doi:10.1093/gigascience/giaa022)
Supplement: giaa022_Supplement_Files [file giaa022_supplement_files.zip › Additional file 1_ Supplementary Methods.docx]

**Supplementary Methods**

Each of the tools PEMA uses has some special characteristics that answer to the amplicon analysis’ demands in a better way, compared to others. In this supplementary file, some of these characteristics are recorded.

***FastQC***

After a dataset has been analysed using PEMA, samples whose .fastq files are of low quality can be excluded from subsequent PEMA runs of this particular dataset. However, removing files completely does not secure that all the remaining reads are of satisfactory quality, as either smaller regions of a read or specific-position errors might have occurred during the sequencing. The next tools in PEMA address these issues.

***Trimmomatic***

As PEMA deals with amplicons, the handling of sequence artifacts erroneously produced because of technology’s limitations is crucial. Further problems may be posed due to the primers and the adapters used, depending on the library preparation and downstream application. Trimmomatic addresses the need of removing such sequences.

When run in ‘Simple mode’, Trimmomatic aims to find approximate matches between the reads and the technical sequences supplied by the user. This way, technical sequences can be identified in any location or orientation within the reads. A considerable overlap between the read and a technical sequence is required to prevent false-positive findings. However, in some cases, the sequenced DNA fragment is shorter than the read length. For these cases, Trimmomatic’s ‘Palindrome mode’ uses both reads and, given the latter are reverse complements, is able to locate short partial adapter sequences; sequences that would otherwise remain undetected as they miss the minimum required overlap.

Trimmomatic, in the aforementioned ways, detects and removes regions of low sequencing quality. However, it is not able to correct a wrong read base. For this task the next algorithm was recruited.

***Cutadapt***

In general, primers, adapter sequences, poly-A tails and other types of unwanted sequence from NGS data are found and then removed by the Cutadapt tool. In PEMA, Cutadapt is used only for the case of the ITS marker gene. That is because the ITS marker gene is highly variable in length due to the high insertion - deletion rate in its evolution. Hence, the removal of primers by taking into account the possibility of read-through into the opposite primer is required.

***BayesHammer***

BayesHammer algorithm tries to

1) find read k-mers whose sequencing quality is above a predefined threshold (“solid k-mers”), and

2) select the sequencing reads that can be rebuilt based on the aforementioned “solid k-mers”.

Thus, after finding the “solid k-mers” BayesHammer can decide whether a base is sequenced properly or not.

***PANDAseq***

PANDAseq attempts to correct possible errors using a probabilistic approach based on the overlap data from the paired-end reads. Further, it uses intrinsic properties of Illumina sequencing, including the low probability of gap inclusion. Finally, it also takes into account the primers that have been used.

PANDAseq supports more than one merging algorithms. ‘Pear’ and ‘simple\_bayesian’ are the ones used most commonly. In PEMA the default merging algorithm is set in ‘simple\_bayesian’; shifting to another PANDAseq-supported algorithm is possible for users that would like to optimize this step via experimentation.

***OBITools***

OBITools is a set of python programs developed to simplify the manipulation of sequence files, especially in the context of NGS-based DNA metabarcoding.

PEMA uses the ‘obiuniq’ program of OBITools with the PANDAseq merged files. A set of fastq files is returned. In the header of each entry, except from the sample name and the sequence id there is also the number of copies of this sequence that were found in this sample.

***VSEARCH***

VSEARCH is an open source and free of charge tool that is meant to be used as an alternative to the USEARCH tool; as on the one hand USEARCH source code is not publicly available and on the other, it is not free distributed.

VSEARCH achieves the processing and preparing of sequence data that arrive from a series of different fields like metagenomics, genomics and population genomics. Among other tasks, VSEARCH supports chimera removal, clustering and dereplication of the sequencing reads. VSEARCH also supports multithreading and as a result takes advantage of this parallelism to perform accurate results at high speed.

*De novo* clustering in VSEARCH is performed by using a greedy and heuristic centroid-based algorithm. An adjustable sequence similarity threshold, e.g., 0.97, can be specified by the user; PEMA also supports for that option.

VSEARCH can be considered as a traditional OTU clustering algorithm as for each cluster, it creates a simple multiple sequence alignment with the centroid as the center sequence, and then compute a consensus sequence and a sequence profile.

***Swarm v2***

In the context of Swarm v2, as “microvariant” it is considered a sequence that differs from the original by one nucleotide (*d* = 1). All possible microvariants of each amplicon are produced and those present in the amplicon pool are recorded. This way, an exact-string search can be performed fast, while at the same time it does not depend on the number of available amplicons present.

The way Swarm infers ASVs should not be affected by chimeras, according to the developers of the algorithm. Chimeras will very likely form independent ASVs. Hence, PEMA checks for chimeras after clustering when Swarm is used. Swarm creates a fasta file containing ASVs representatives and with this file PEMA runs a chimera check, using again VSEARCH algorithm. With the output of ‘uchime3_denovo’, PEMA builds an ASV-table without taxonomies.

In the “.stats” file that Swarm produces, the number of unique amplicons in each ASV, the total abundance of amplicons in it, the identifier of the initial seed, the abundance of that initial seed and the number of amplicons with abundance 1 stand for the first 6 columns of the file, respectively. The last two columns represent the maximum number of iterations before the MOTU reached its natural limit and the cumulative number of steps along the path joining the seed and the furthermost amplicon in the ASV. Furthermore, in case that parameter *d* equals to 1, all first five columns are modified, as a second clustering pass is performed to reduce the number of small ASVs, but not the last two.

***CREST & SILVA***

The ‘LCAClassifier’ algorithm is used for the classification of sequences aligned to the reference databases provided. CREST can handle 4 databases: SILVA, Greengenes, Unite and amoA. Moreover, it allows the user to build his/her own database.

***RDPClassifier & Midori***

RDPClassifier is a naive Bayesian classifier, compatible with the Midori reference datasets. RDPClassifier automatically estimates the classification reliability by making use of the bootstrap method for each rank assignment. Bootstrap confidence reflects the frequency of most common assignments out of 100 bootstrap samplings. The default threshold is 80% but the assignment confidence cutoff that determines the assignment count for each taxon, can be set by the user as well.

Midori includes all metazoan mitochondrial gene sequences from GenBank, quality filtered and formatted for taxonomic assignments. The RDPClassifier was trained with the Midori database (version: MIDORI_LONGEST_1.1) during PEMA’s development and can be used for the taxonomic assignment for the case of the COI marker gene.

***BigDataScript programming language (BDS)***

PEMA is a very fast pipeline for a metabarcoding analysis. This is mainly due to the BDS programming language that allows absolute serialization and lazy processing. This way, even in the case that an error takes place, PEMA is able to restart from the last checkpoint that was created, before the error occurred. In addition, when PEMA has to perform a “for” loop, then BDS splits that task in all cores available. Then, thanks to the “wait” command that BDS includes, it just stops until everything needed for the next step of the pipeline is completed. However, even if BDS has a lot of advantages, some of the tools included in BDS cannot exploit them. That is why, even if in some steps there are nodes available, PEMA does not use them.

After a failure of a task, the program can be re-executed from that specific point and there is no need to re-run it from scratch. This is also possible in case a pipeline has more than one possible arguments in a specific step. Re-executing the pipeline from a specific step, after changing a specific parameter, is possible with BDS checkpoints. This is quite convenient for a pipeline like PEMA as after the completion of an analysis, if the user wishes for a single parameter of a single step to be changed, PEMA can be executed only from the point that this parameter appears and not earlier. For example, even if P.E.M.A has been executed with Swarm as clustering algorithm, BDS checkpoints allow the user to rerun PEMA with CROP as clustering algorithm, only from the clustering step and forward.

**Licenses**

- BDS Apache License 2 (<http://www.apache.org/licenses/LICENSE-2.0>)
- Trimmomatic, PANDAseq, GNU GPLv3 (<https://www.gnu.org/licenses/gpl-3.0.en.html>)
  PaPaRa, VSEARCH,
  CREST, Fastqc, RAxML-ng,
  Crop, EPA-ng
- Phyloseq, vegan, Swarm v2 AGPLv3 (<https://opensource.org/licenses/AGPL-3.0>)
- Blastn (NCBI BLAST) Public Domain Notice ([https://www.ncbi.nlm.nih.gov/IEB/ToolBox
   /CPP_DOC/lxr/source/scripts/projects/blast/LICENSE](https://www.ncbi.nlm.nih.gov/IEB/ToolBox/CPP_DOC/lxr/source/scripts/projects/blast/LICENSE))
- Spades, RDPTools GNU GPLv2 (<https://opensource.org/licenses/GPL-2.0> )
- OBITools cecill ([http://www.cecill.info/licences/Licence_CeCILL_V2.1
   -en.html](http://www.cecill.info/licences/Licence_CeCILL_V2.1-en.html))
- Mafft BSD license (<https://mafft.cbrc.jp/alignment/software/license.txt>)
- Cutadapt MIT License ([https://github.com/marcelm/cutadapt/blob/master/](https://github.com/marcelm/cutadapt/blob/master/LICENSE)

[LICENSE](https://github.com/marcelm/cutadapt/blob/master/LICENSE) )

### **Comparison of pipelines**

For reference, we include the commands used to execute QIIME 2, mothur, LotuS and PEMA on the chosen dataset. The official QIIME 2 tutorial (<https://docs.qiime2.org/2019.4/tutorials/moving-pictures/>), mothur SOP (<https://www.mothur.org/wiki/MiSeq_SOP>) and LotuS tutorial (<http://psbweb05.psb.ugent.be/lotus/documentation.html#Tutorial>) were followed.

***QIIME 2 commands***

In order to import our data we ran:

qiime tools import **\**

--type 'SampleData[PairedEndSequencesWithQuality]' **\**

--input-path pe-64-manifest **\**

--output-path paired-end-demux.qza **\**

--input-format PairedEndFastqManifestPhred64V2

Then, the two different approaches that QIIME 2 supports were both performed.

Hence, for the DADA2 approach, the below commands were performed:

qiime dada2 denoise-single **\**

--i-demultiplexed-seqs paired-end-demux.qza **\**

--p-trim-left 0 **\**

--p-trunc-len 120 **\**

--o-representative-sequences rep-seqs-dada2.qza **\**

--o-table table-dada2.qza **\**

--o-denoising-stats stats-dada2.qza

qiime metadata tabulate **\**

--m-input-file stats-dada2.qza **\**

--o-visualization stats-dada2.qzv

mv rep-seqs-dada2.qza rep-seqs.qza

mv table-dada2.qza table.qza

While for the Deblur approach, the following commands were executed instead:

qiime quality-filter q-score **\**

--i-demux paired-end-demux.qza **\**

--o-filtered-sequences demux-filtered.qza **\**

--o-filter-stats demux-filter-stats.qza

qiime deblur denoise-16S **\**

--i-demultiplexed-seqs demux-filtered.qza **\**

--p-trim-length 120 **\**

--o-representative-sequences rep-seqs-deblur.qza **\**

--o-table table-deblur.qza **\**

--p-sample-stats **\**

--o-stats deblur-stats.qza

qiime metadata tabulate **\**

--m-input-file demux-filter-stats.qza **\**

--o-visualization demux-filter-stats.qzv

qiime deblur visualize-stats **\**

--i-deblur-stats deblur-stats.qza **\**

--o-visualization deblur-stats.qzv

mv rep-seqs-deblur.qza rep-seqs.qza

mv table-deblur.qza table.qza

After that, for both cases the succeeding commands were performed:

In order to get feature table and feature data summaries:

qiime feature-table summarize **\**

--i-table table.qza **\**

--o-visualization table.qzv **\**

--m-sample-metadata-file sample-metadata.tsv

qiime feature-table tabulate-seqs **\**

--i-data rep-seqs.qza **\**

--o-visualization rep-seqs.qzv

And for the taxonomy assignment of the OTUs found:

qiime feature-classifier classify-sklearn **\**

--i-classifier gg-13-8-99-515-806-nb-classifier.qza **\**

--i-reads rep-seqs.qza **\**

--o-classification taxonomy.qza

qiime metadata tabulate **\**

--m-input-file taxonomy.qza **\**

--o-visualization taxonomy.qzv

***mothur commands***

The mothur sbatch file was set as such:

#This is a batch file for running the equivalent of mothur

make.file(inputdir=/home1/haris/MyData_mothur/, type=fastq, prefix=mapping)

make.contigs(file=mapping.files, processors=8)

summary.seqs(fasta=mapping.trim.contigs.fasta)

screen.seqs(fasta=mapping.trim.contigs.fasta, group=mapping.contigs.groups, summary=mapping.trim.contigs.summary, maxambig=0, minlength=100, maxlength=600, maxhomop=8)

unique.seqs(fasta=mapping.trim.contigs.good.fasta)

count.seqs(name=mapping.trim.contigs.good.names, group=mapping.contigs.good.groups)

summary.seqs(count=mapping.trim.contigs.good.count_table)

pcr.seqs(fasta=silva.nr_v132.align, start=1044, end=43116, keepdots=T, processors=8)

rename.file(input=silva.nr_v132.pcr.align, new=silva.fasta)

summary.seqs(fasta=silva.fasta)

align.seqs(fasta=mapping.trim.contigs.good.unique.fasta, reference=silva.fasta)

summary.seqs(fasta=mapping.trim.contigs.good.unique.align, count=mapping.trim.contigs.good.count_table)

screen.seqs(fasta=mapping.trim.contigs.good.unique.align, count=mapping.trim.contigs.good.count_table, summary=mapping.trim.contigs.good.unique.summary, start=6388, end=25316, maxhomop=8)

summary.seqs(fasta=mapping.trim.contigs.good.unique.good.align, count=mapping.trim.contigs.good.good.count_table)

filter.seqs(fasta=mapping.trim.contigs.good.unique.good.align, vertical=T, trump=.)

unique.seqs(fasta=mapping.trim.contigs.good.unique.good.filter.fasta, count=mapping.trim.contigs.good.good.count_table)

pre.cluster(fasta=mapping.trim.contigs.good.unique.good.filter.unique.fasta, count=mapping.trim.contigs.good.unique.good.filter.count_table, diffs=2)

chimera.vsearch(fasta=mapping.trim.contigs.good.unique.good.filter.unique.precluster.fasta, count=mapping.trim.contigs.good.unique.good.filter.unique.precluster.count_table, dereplicate=t)

remove.seqs(fasta=mapping.trim.contigs.good.unique.good.filter.unique.precluster.fasta, accnos=mapping.trim.contigs.good.unique.good.filter.unique.precluster.denovo.vsearch.accnos)

classify.seqs(fasta=mapping.trim.contigs.good.unique.good.filter.unique.precluster.pick.fasta, count=mapping.trim.contigs.good.unique.good.filter.unique.precluster.denovo.vsearch.pick.count_table, reference=silva.nr_v132.align, taxonomy=silva.nr_v132.tax, cutoff=80)

summary.tax(taxonomy=mapping.trim.contigs.good.unique.good.filter.unique.precluster.pick.nr_v132.wang.taxonomy, count=mapping.trim.contigs.good.unique.good.filter.unique.precluster.denovo.vsearch.pick.count_table)

dist.seqs(fasta=mapping.trim.contigs.good.unique.good.filter.unique.precluster.pick.fasta, cutoff=0.03)

cluster(column=mapping.trim.contigs.good.unique.good.filter.unique.precluster.pick.dist, count=mapping.trim.contigs.good.unique.good.filter.unique.precluster.denovo.vsearch.pick.count_table)

make.shared(list=mapping.trim.contigs.good.unique.good.filter.unique.precluster.pick.opti_mcc.list, count=mapping.trim.contigs.good.unique.good.filter.unique.precluster.denovo.vsearch.pick.count_table, label=0.03)

classify.otu(list=mapping.trim.contigs.good.unique.good.filter.unique.precluster.pick.opti_mcc.list, count=mapping.trim.contigs.good.unique.good.filter.unique.precluster.denovo.vsearch.pick.count_table, taxonomy=mapping.trim.contigs.good.unique.good.filter.unique.precluster.pick.nr_v132.wang.taxonomy, label=0.03)

rename.file(taxonomy=mapping.trim.contigs.good.unique.good.filter.unique.precluster.pick.opti_mcc.0.03.cons.taxonomy, shared=mapping.trim.contigs.good.unique.good.filter.unique.precluster.pick.opti_mcc.shared)

count.groups(shared=mapping.opti_mcc.shared)

rarefaction.single(shared=mapping.opti_mcc.shared, calc=sobs, freq=100)

summary.single(shared=mapping.opti_mcc.shared, calc=nseqs-coverage-sobs-invsimpson)

Then, mothur was performed running the command:

/mnt/big/mothur-1.42.1/mothur mothur.batch

***LotuS commands and parameters***

The sdm_miSeq.txt file was set as such:

#sdm options file to control sequence quality filtering, demultiplexing and preparation (can also be used without demultiplexing)

#* indicates alternative quality filtering options, saved in *.add.fna etc. files separately from initial quality filtered dataset

#sequence length refers to sequence length AFTER removal of Primers, Barcodes and trimming. this ensures that downstream analyis tools will have appropiate sequence information

#options with a star in front are lenient parameters for mid qual sequences (only used for estimating OTU abundance, not for OTU building itself).

minSeqLength 150

maxSeqLength 350

minAvgQuality 30

*minSeqLength 150

*minAvgQuality 30

#truncate total Sequence length to X (length after Barcode, Adapter and Primer removals, set to -1 to deactivate)

TruncateSequenceLength -1

#Ambiguous bases in Sequence

maxAmbiguousNT 1

*maxAmbiguousNT 1

#sequence is discarded if a homonucleotide run in sequence is longer

maxHomonucleotide 8

#Filter whole sequence if one window of quality scores is below average

QualWindowWidth 50

QualWindowThreshhold 25

#Trim the end of a sequence if a window falls below quality threshhold. Useful for removing low qulaity trailing ends of sequence

TrimWindowWidth 20

TrimWindowThreshhold 25

#Probabilistic max number of accumulated sequencing errors. After this length, the rest of the sequence will be deleted. Complimentary to TrimWindowThreshhold. (-1) deactivates this option.

maxAccumulatedError 0.75

*maxAccumulatedError -1

#Binomial error model of expected errors per sequence (see https://github.com/fpusan/moira), to deactivate, set BinErrorModelAlpha to -1

BinErrorModelMaxExpError 2.5

BinErrorModelAlpha -1

#Max Barcode Errors

maxBarcodeErrs 0

maxPrimerErrs 0

#keep Barcode / Primer Sequence in the output fasta file - in a normal 16S analysis this should be deactivated (0) for Barcode and de-activated (0) for primer

keepBarcodeSeq 0

keepPrimerSeq 0

#set fastqVersion to 1 if you use Sanger, Illumina 1.8+ or NCBI SRA files. Set fastqVersion to 2, if you use Illumina 1.3+ - 1.7+ or Solexa fastq files. "auto" will look for typical characteristics of either of these and choose the quality offset score automatically.

fastqVersion auto

#if one or more files have a technical adapter still included (e.g. TCAG 454) this can be removed by setting this option

TechnicalAdapter

#delete X NTs (e.g. if the first 5 bases are known to have strange biases)

TrimStartNTs 0

#correct PE header format (1/2) this is to accomodate the illumina miSeq paired end annotations 2="@XXX 1:0:4" insteand of 1="@XXX/1". Note that the format will be automatically detected

PEheaderPairFmt 1

#sets if sequences without match to reverse primer (ReversePrimer) will be accepted (T=reject ; F=accept all); default=F

RejectSeqWithoutRevPrim F

#*RejectSeqWithoutRevPrim F

#sets if sequences without a forward (LinkerPrimerSequence) primer will be accepted (T=reject ; F=accept all); default=F

RejectSeqWithoutFwdPrim F

#*RejectSeqWithoutFwdPrim F

#this option should be "T" if your amplicons are possibly shorter than a single read in a paired end sequencing run (e.g. if the 16S amplicon length is 200bp in a 250x2 miSeq run, set this to "T"). This option increases runtime by 10%, if in doubt just set to "T". *Requires* LinkerPrimerSequence and ReversePrimer to be defined in mapping file.

AmpliconShortPE F

#options for difficulties during sequencing library construction

#checks if pair1 and pair2 were switched (ignore if single read data)

CheckForMixedPairs F

#checks if whole amplicon was reverse-transcribed sequenced (not switched, just reverse translated)

CheckForReversedSeqs F

Then, LotuS was performed running the command:

Perl /home1/haris/lotus_pipeline/lotus.pl -i /home1/haris/MyData/ -o /home1/haris/MyData_LotuS/output/ -m /home1/haris/MyData_LotuS/mymap.txt -c /home1/haris/lotus_pipeline/lOTUs.cfg -s /home1/haris/MyData_LotuS/sdm_miSeq.txt -keepTmpFiles 1 -highmem 1 -p miSeq -itsextraction 0 -simBasedTaxo 2 -refDB SLV

***Barque commands and parameters***

# Global parameters

NCPUS=20 # Number of CPUs to use for analyses (int, 1+)

PRIMER_FILE="02_info/primers.csv" # File with PCR primers information

# Skip data preparation and rerun only from vsearch

SKIP_DATA_PREP=0 # 1 to skip data preparation steps, 0 to run full pipeline

# Filtering with Trimmomatic

CROP_LENGTH=300 # Cut reads to this length after filtering

# Merging reads with flash

MIN_OVERLAP=10 # Minimum number of overlapping nucleotides to merge

reads (int, 1+)

MAX_OVERLAP=280 # Maximum number of overlapping nucleotides to merge

reads (int, 1+)

# Extracting barcodes

MAX_PRIMER_DIFF=1 # Maximum number of differences allowed between primer

and sequence (int, 0+)

# Running or skipping chimera detection

SKIP_CHIMERA_DETECTION=0 # 0 to search for chimeras (RECOMMENDED), 1 to skip

chimera detection or use already created chimera cleaned files

# vsearch

MAX_ACCEPTS=20 # Accept at most this number of sequences before stoping

search (int, 1+)

MAX_REJECTS=20 # Reject at most this number of sequences before stoping

search (int, 1+)

QUERY_COV=0.6 # At least that proportion of the sequence must match the

database (float, 0-1)

# Filters

MIN_HIT_LENGTH=230 # Minimum vsearch hit length to keep in results (int, 1+)

MIN_HITS_SAMPLE=1 # Minimum number of hits a species must have in at least

one sample to keep in results (int, 1+)

# Non-annotated reads

NUM_NON_ANNOTATED_SEQ=1000 # Number of unique most-frequent non-annotated reads to

keep (int, 1+)

# OTUs

SKIP_OTUS=1 # Skip OTU creation

MIN_SIZE_FOR_OTU=20 # Only unique reads with at least this coverage will be used

for OTUs

***PEMA command and parameters***

The parameters file for the case of the 16S rRNA marker gene and the [30] dataset was set as such:

outputFolderName amvrakikos_silva132_final

EnaData Yes

maxInfo Yes

targetLength 200

strictness 0.3

adapters TruSeq2-PE.fa

seedMismatches 0

palindromeClipThreshold 20

simpleClipThreshold 30

leading 20

trailing 20

minlen 100

threadsTrimmomatic 20

pandaseqAlgorithm simple_bayesian

vsearchThreads 20

vsearchId 0.98

gene gene_16S

taxonomyAssignmentMethod alignment

silvaVersion silva_132

taxonomyFolderName 16S_taxon_assign

phyloseq No

raxmlThreads 20

parsTrees 10

bootstrapTrees 100

emptyRawDataFile Yes

emptyCheckpoints Yes

Then, PEMA was performed running the command:

singularity run -B /home1/haris/16S_analysis/:/mnt /home1/haris/metabar_pipeline/pema.simg

Similarly, for the case of the COI marker gene and the [31] dataset, the parameters were the following:

outputFolderName lakes_235bp_only_d_10

EnaData Yes

targetLength 235

strictness 0.4

adapters TruSeq2-PE.fa

seedMismatches 1

palindromeClipThreshold 20

simpleClipThreshold 30

leading 20

trailing 20

minlen 100

spadesAlgorithm simple_bayesian

vsearchThreads 20

clusteringAlgo algo_SWARM

d 10

abskew 2

emptyRawDataFile No

emptyCheckpoints Yes

For the rest of the runs that are presented in this study, the only changes in the parameter set above, was the value of the cluster radius (*d*) of the Swarm algorithm. Obviously, the “outputFolderName” was also changed correspondingly in each run.
