# Supplementary material for: PEMA: a flexible Pipeline for Environmental DNA Metabarcoding Analysis of the 16S/18S ribosomal RNA, ITS, and COI marker genes
Source: Gigascience. 2020 Mar 12;9(3):giaa022. doi: 10.1093/gigascience/giaa022 (PMC7066391; doi:10.1093/gigascience/giaa022)
Supplement: giaa022_Supplement_Files [file giaa022_supplement_files.zip › Additional file 4_Table S2.docx]

**Table S2: Diversity indices of the samples.**

|  | **LotuS** | | **Deblur** | | **DADA2** | | **PEMA** | |
| --- | --- | --- | --- | --- | --- | --- | --- | --- |
| **Samples** | **OTUs** | **N** | **OTUs** | **N** | **OTUs** | **N** | **OTUs** | **N** |
| L_LOout_A | 2451 | 67640 | 156 | 5878 | 177 | 47954 | 791 | 17888 |
| L_LOout_B | 3432 | 95835 | 180 | 5978 | 248 | 62416 | 1182 | 19571 |
| L_LOout_C | 2987 | 97592 | 180 | 9216 | 221 | 59346 | 1048 | 24947 |
| L_LOinA | 3656 | 85882 | 200 | 6284 | 264 | 62253 | 1176 | 18346 |
| L_LOinB | 2935 | 76545 | 173 | 6357 | 194 | 46191 | 954 | 18750 |
| L_LOinC | 3149 | 71222 | 183 | 5849 | 219 | 49890 | 1012 | 15940 |
| S_KalA | 2467 | 59039 | 82 | 2991 | 173 | 46418 | 668 | 10086 |
| S_KalB | 2940 | 57091 | 89 | 2107 | 192 | 49715 | 802 | 7702 |
| S_KalC | 2898 | 61191 | 101 | 2934 | 214 | 51321 | 769 | 8840 |
| R_ARDelta_A | 3079 | 53780 | 97 | 1341 | 259 | 53237 | 737 | 4386 |
| R_ARDelta_B | 3671 | 63857 | 117 | 1893 | 271 | 62477 | 836 | 5716 |
| R_ARDelta_C | 3323 | 50640 | 100 | 1389 | 230 | 48650 | 703 | 4247 |
| R_AR_A | 3084 | 58311 | 152 | 3000 | 269 | 52689 | 1058 | 10208 |
| R_AR_B | 4011 | 84370 | 199 | 3931 | 304 | 81589 | 1546 | 14058 |
| R_AR_C | 3674 | 97462 | 196 | 5290 | 343 | 83000 | 1501 | 16819 |
| R_ARO_A | 3079 | 63781 | 123 | 1993 | 324 | 60752 | 1000 | 7381 |
| R_ARO_B | 3178 | 92008 | 183 | 5352 | 359 | 64824 | 1307 | 17052 |
| R_ARO_C | 3248 | 89992 | 186 | 5540 | 307 | 61082 | 1255 | 16730 |

OTUs: total number of OTUs. N: total microbial relative abundance values. All sample libraries that start with “L” correspond to samples collected from lagoons, with “S” from the sea while “R” stands for the riverine samples. AR: Arachthos. ARO: Arachthos Neochori. ARDelta: Arachthos Delta. LOin: Logarou station inside the lagoon. LOout: Logarou station in the channel connecting the lagoon to the gulf. Kal: Kalamitsi. A, B, C replicate samples.
