# Supplementary figures and images for: PEMA: a flexible Pipeline for Environmental DNA Metabarcoding Analysis of the 16S/18S ribosomal RNA, ITS, and COI marker genes
Source: Gigascience. 2020 Mar 12;9(3):giaa022. doi: 10.1093/gigascience/giaa022 (PMC7066391; doi:10.1093/gigascience/giaa022)

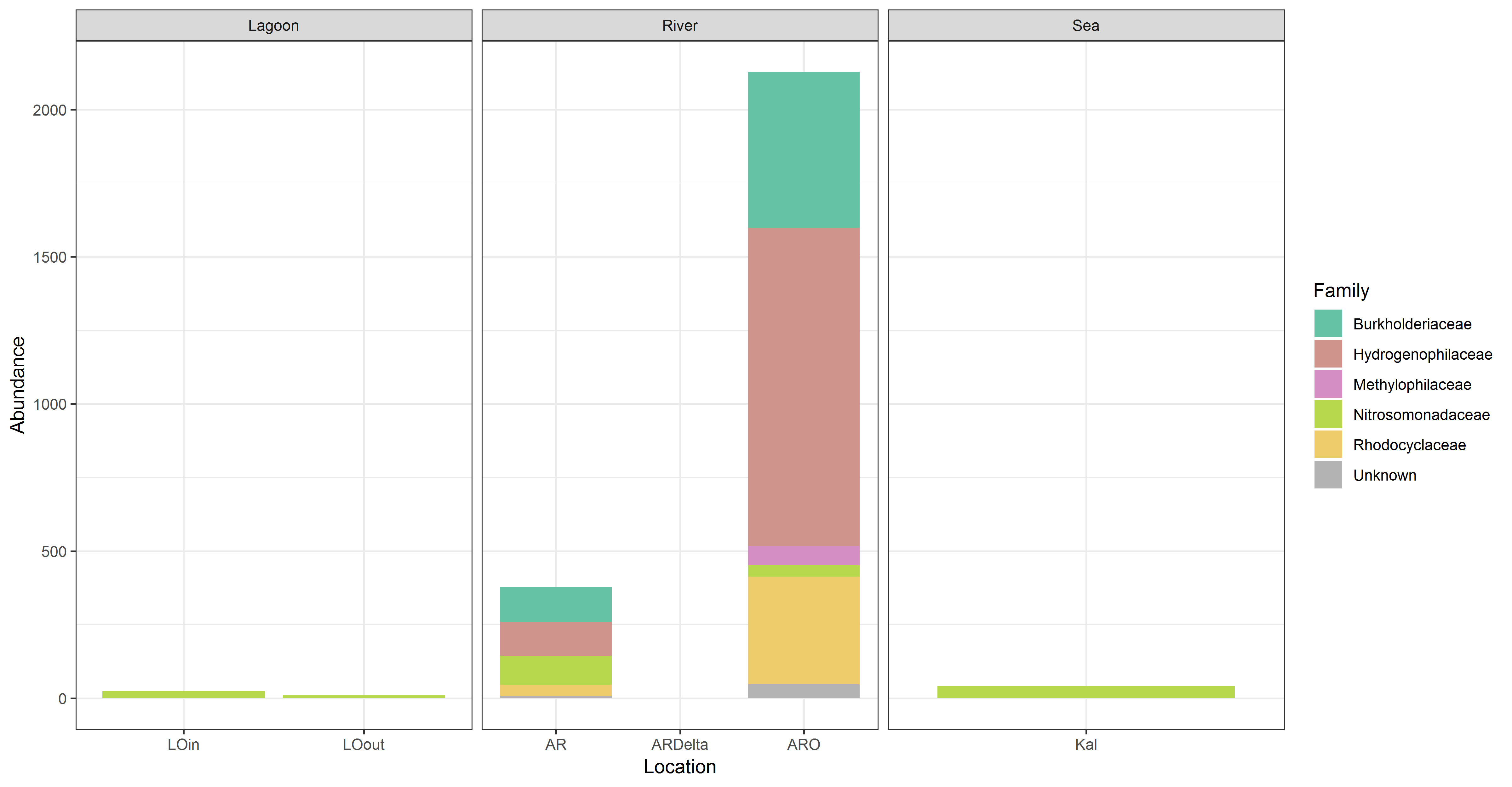

Supplement: giaa022_Supplement_Files [file giaa022_supplement_files.zip › Additional file 10_Figure S6.png]

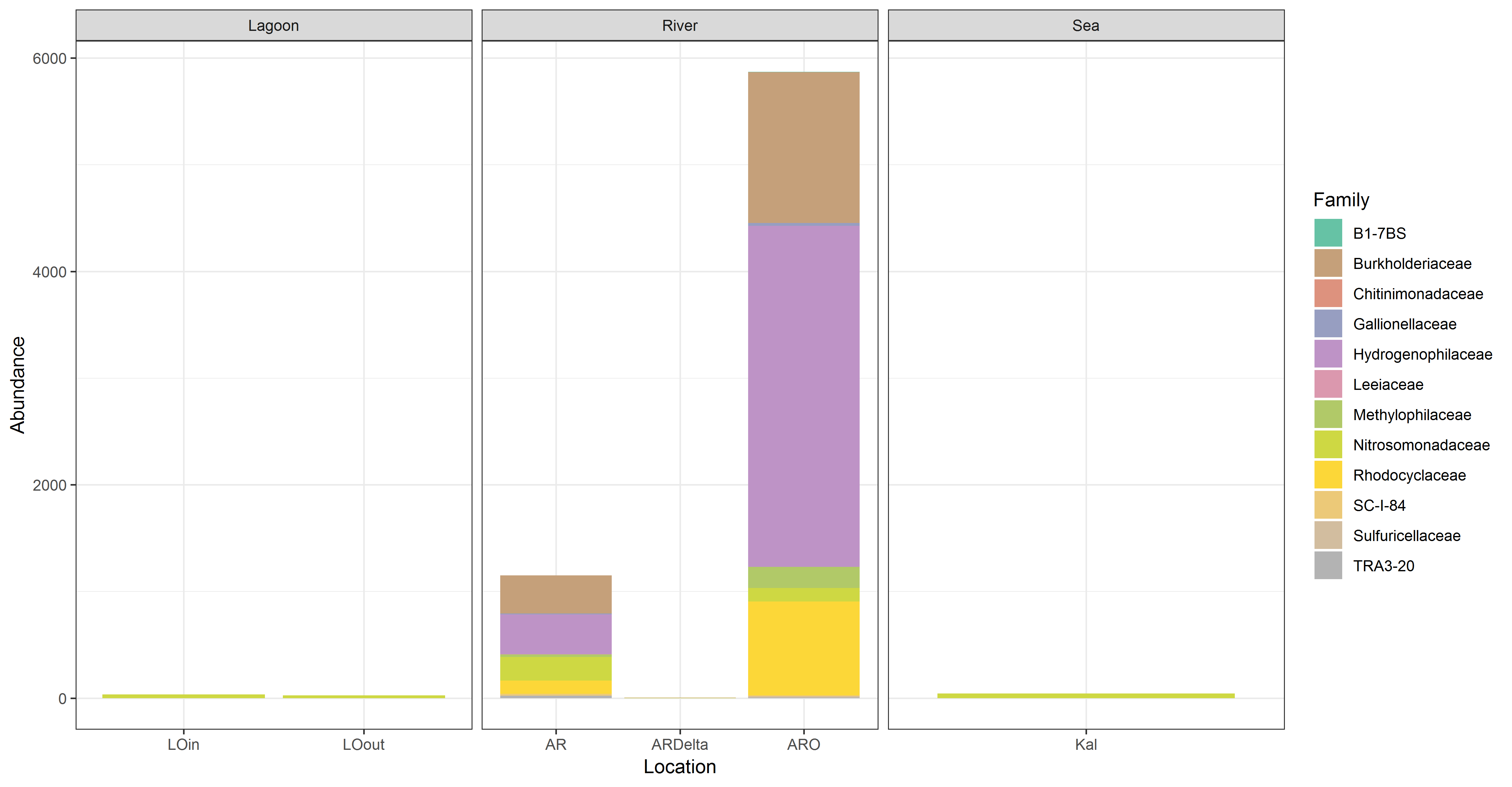

Supplement: giaa022_Supplement_Files [file giaa022_supplement_files.zip › Additional file 11_Figure S7.png]

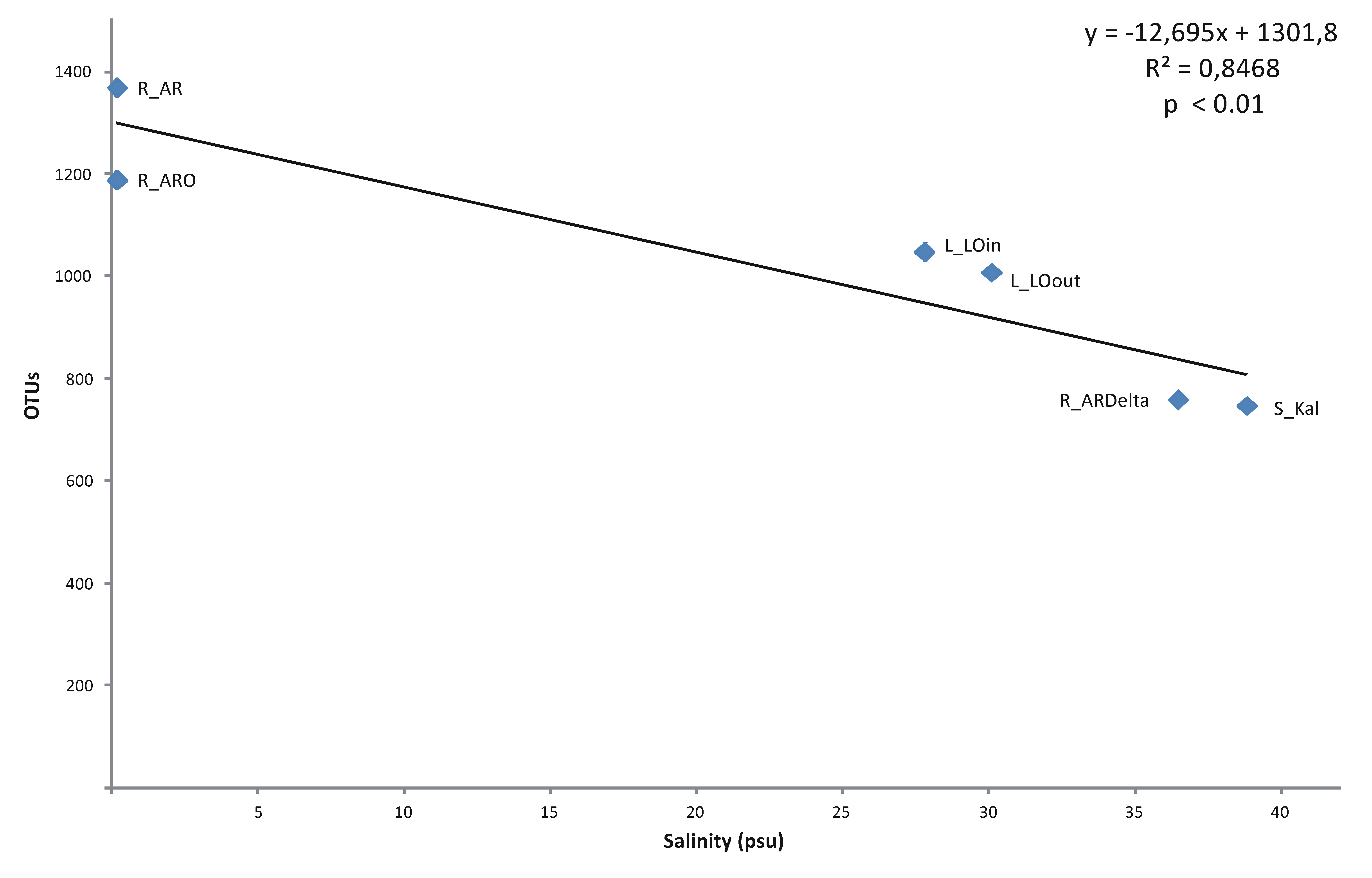

Supplement: giaa022_Supplement_Files [file giaa022_supplement_files.zip › Additional file 5_ Figure S1.png]

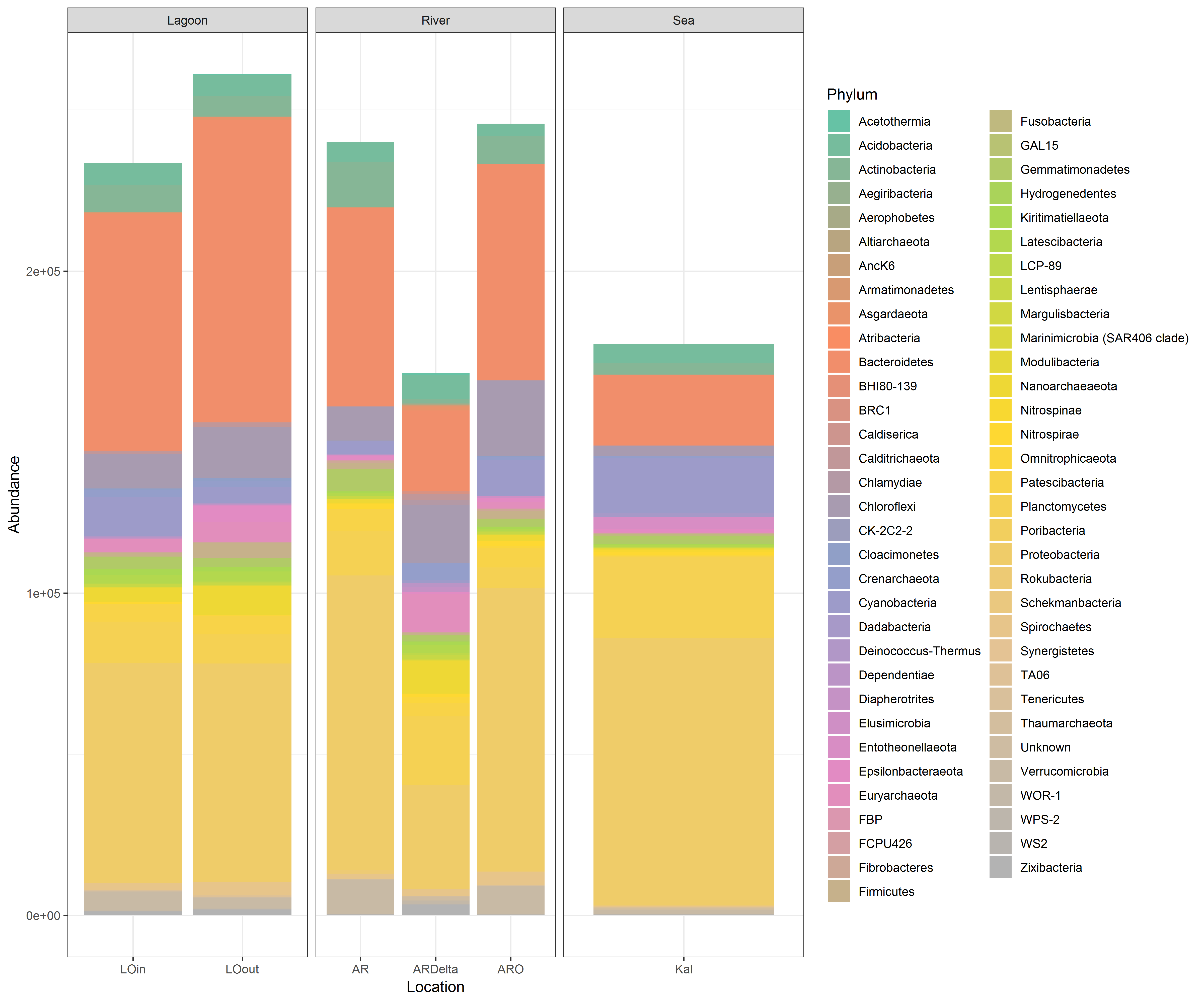

Supplement: giaa022_Supplement_Files [file giaa022_supplement_files.zip › Additional file 6_Figure S2.png]

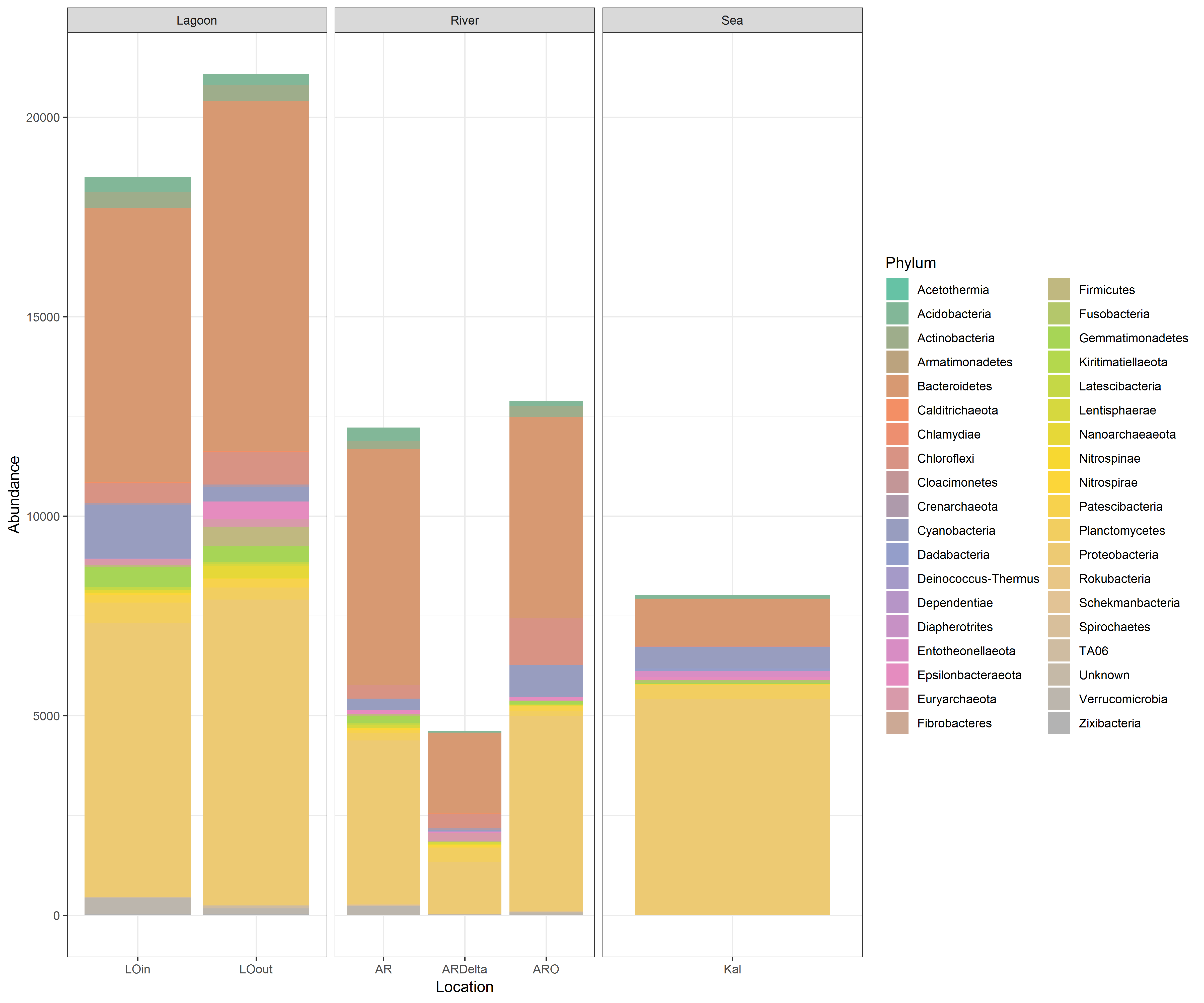

Supplement: giaa022_Supplement_Files [file giaa022_supplement_files.zip › Additional file 7_Figure S3.png]

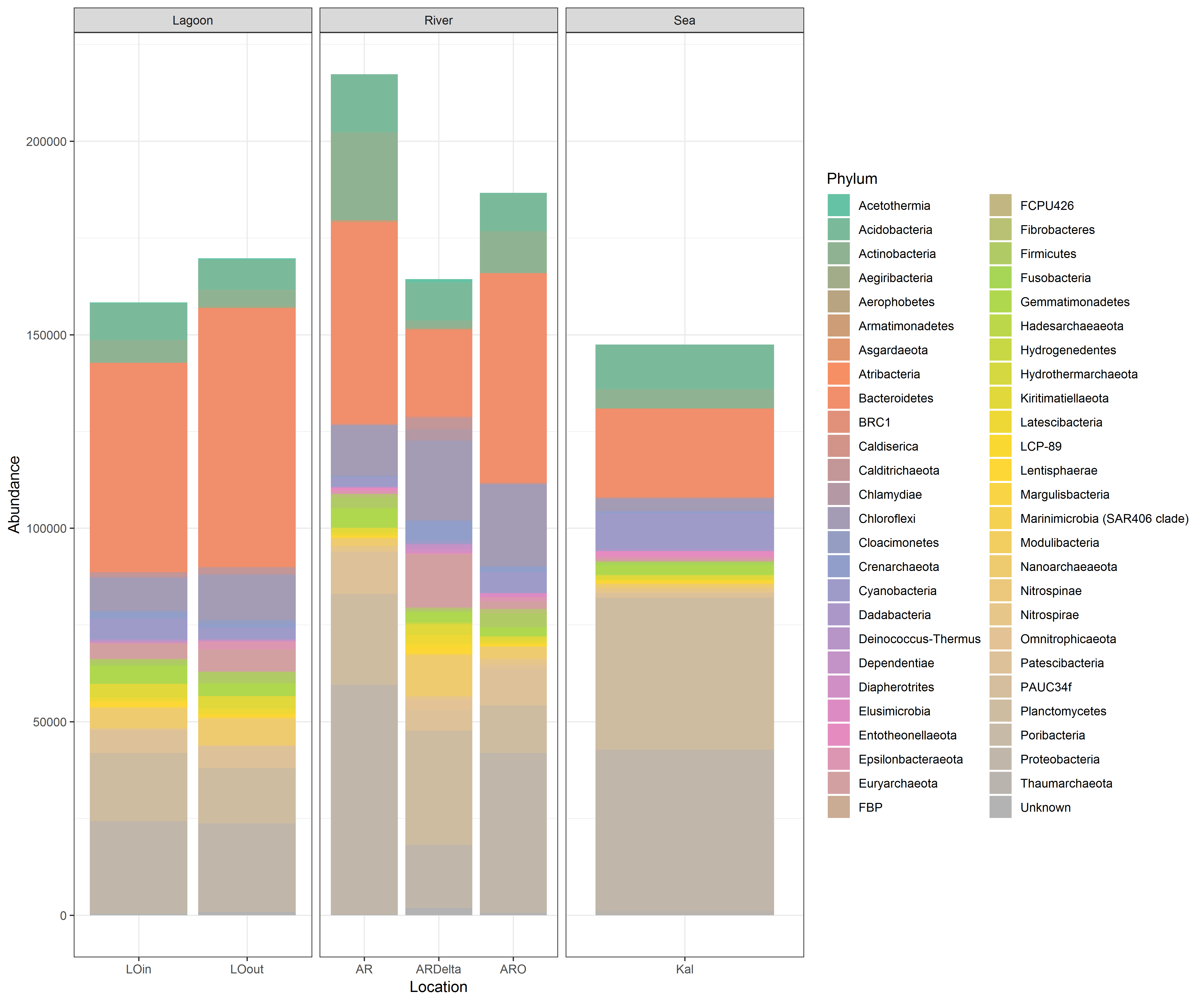

Supplement: giaa022_Supplement_Files [file giaa022_supplement_files.zip › Additional file 8_Figure S4.png]

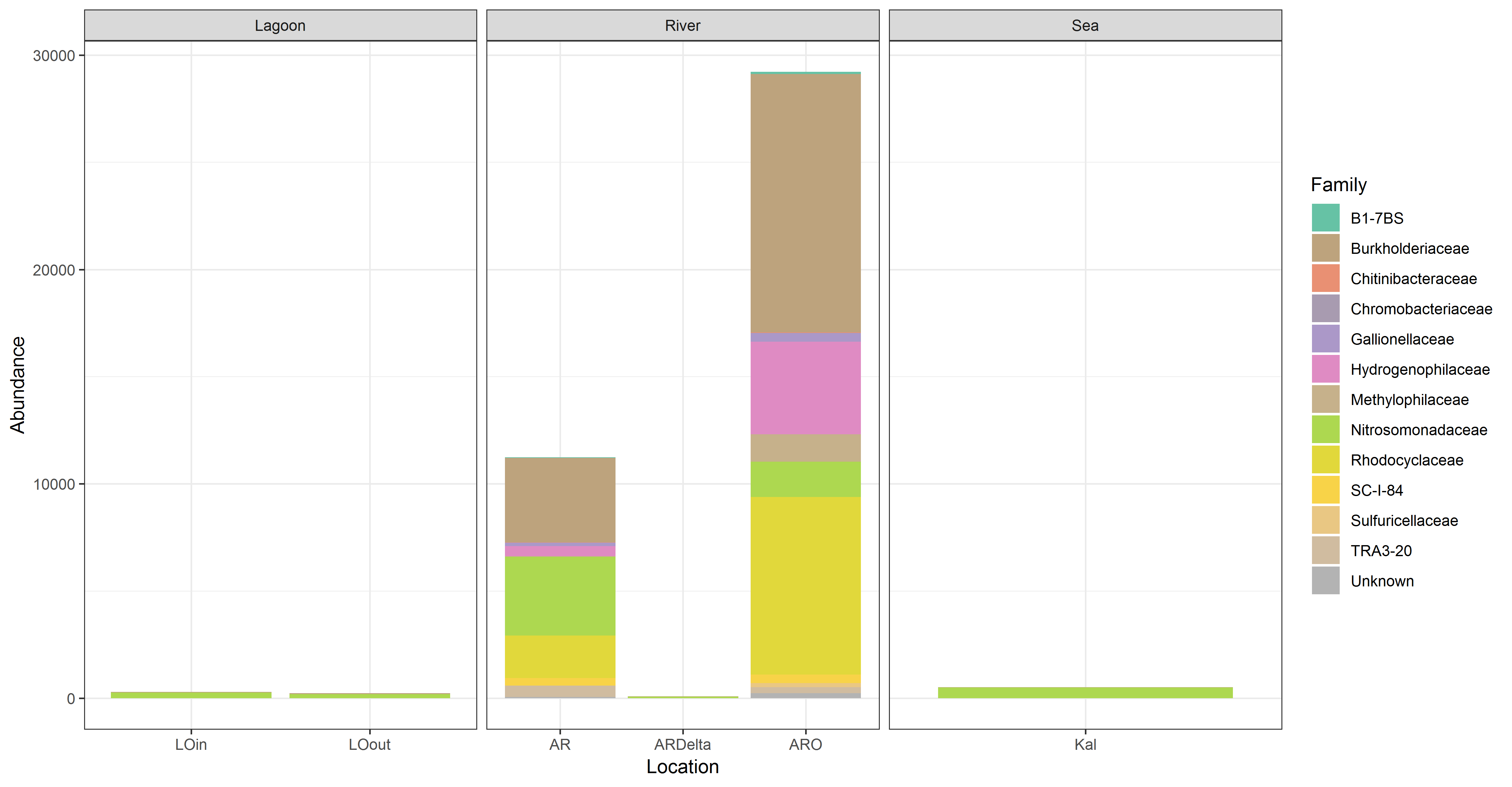

Supplement: giaa022_Supplement_Files [file giaa022_supplement_files.zip › Additional file 9_Figure S5.png]
