# Supplementary material for: PEMA: a flexible Pipeline for Environmental DNA Metabarcoding Analysis of the 16S/18S ribosomal RNA, ITS, and COI marker genes
Source: Gigascience. 2020 Mar 12;9(3):giaa022. doi: 10.1093/gigascience/giaa022 (PMC7066391; doi:10.1093/gigascience/giaa022)
Supplement: giaa022_Response_to_Reviewer_Comments_Original_Submission [file giaa022_response_to_reviewer_comments_original_submission.pdf]

Author's Response To Reviewer Comments

Close

Response to the editor’s and reviewer’s comments

We would like to kindly thank the reviewer for the time he spent to thoroughly read our manuscript. We sincerely appreciate all accurate comments made by the reviewer, which helped the improvement of our manuscript. In the revised version, we have addressed all of the reviewer’s comments and suggestions and where necessary we have incorporated changes and made amendments and alterations to the manuscript. The changes and the new sections of the manuscript are written in blue. Below we cite our detailed answers (in blue) to the editor and reviewers’ comments and suggestions (italic).

Editor’s comment #1
In particular, the section on comparing the tool with existing pipelines needs more attention with respect to the way this comparison is presented in the manuscript. As the reviewer says, "PEMA allows to switch from one (existing) tool to another at each step of the filtering process, therefore differences are the result of the combination of tools decided by the user, not of PEMA itself."
Switching tools without having to re-run an analysis from scratch, is one of PEMA’s main advantages. Providing the user the ability for a great number of tests, while tuning numerous parameters of the tools selected, PEMA allows for a thorough benchmarking for each and every study. Regarding the way that the comparison between PEMA and other metabarcoding pipelines is presented, please see Editor’s comment #2 and Reviewer’s comment #2.

Editor’s comment #2
I do feel that comparisons to similar tools are an important aspect for our Technical Notes, but please make sure that the reader is not getting potentially misleading impressions.
A paragraph explaining both the purpose and the limitations of comparing pipelines has been added as a “preface” in the “Evaluation on real datasets and against other tools” section (lines 345-352 of the revised version of the manuscript). Certain changes have been made in the manuscript to clarify any potential misconception regarding this issue (e.g. lines 389-390).

Editor’s comment #3
If you have not yet done so, please register your new software application in the bio.tools and SciCrunch.org databases to receive RRID (Research Resource Identification Initiative ID) and biotoolsID identifiers, and include these in your manuscript. This will facilitate tracking, reproducibility and re-use of your tool.
PEMA was not registered in bio.tools or SciCrunc.org databases until the last submission. Now it has been registered in both and has the unique ids: “PEMA” (in biotools) and “SCR_017676” (RRID in SciCrunch.org). They have both been included in the revised PEMA manuscript.

Reviewer’s comment #1
I have reviewed for the second time the manuscript from Zafeiropoulos et al. "PEMA: a flexible Pipeline for Environmental DNA Metabarcoding Analysis of the 16S/18S rRNA, ITS and COI marker genes". First, I would like to acknowledge the major improvements implemented by the authors in their pipeline since the first submission, namely: the extension of the functionality to two other marker genes (18S rRNA and ITS) and the inclusion of tools for inferring ASV. This is undoubtedly a great set of additional tools, and the pipeline now covers four of the most common markers for eDNA studies (but not all marker genes as mentioned in the responses). That said, attending that similar pipelines already exist (see PipeCraft for another example; Anslan et al. 2017 Molecular Ecology), I think the main strength of this pipeline is in flexibility, time efficiency and ability to handle large datasets.
Thank you for your patience for a second review. It is the most appreciated.
With respect to PipeCraft, unfortunately we did not achieve to test it. In their paper, Anslan et al (2017)

write that it is available through PlutoF system (download link https://plutof.ut.ee/#/datacite/10.15156%2FBIO%2F587450), however the link does not work. In addition, PipeCraft is not registered in any other repository, making it impossible for end-users to reach it.

Reviewer's comment #2
I appreciate the inclusion of mock community analyses that, contrarily to real eDNA dataset, allow the reader to have an idea of what to expect and easily compare outputs of the pipeline with the biological reality (although this section could gain in readability by avoiding study-specific details). However, I am still very puzzled by the comparisons with other software. I agree with the authors when they say that "it is important to assess the variability that different tools introduce in the produced outputs of each study", and PEMA allows that by offering different options at every step of the pipeline, but it seems abusive to say that results from PEMA outperforms those of other pipelines (e.g. lines 370-372). Comparing pipelines make sense when there is algorithm development but here PEMA allows to switch from one (existing) tool to another at each step of the filtering process, therefore differences are the result of the combination of tools decided by the user, not of PEMA itself.
In my opinion, these comparisons can appear as misleading, and make the paper more complicated and longer than needed.
Regarding the sentence in lines 370-372 (previous version of the manuscript), it has been rephrased (lines 389-390 of the revised version of the manuscript), hopefully not causing misconceptions. In addition, in the paragraph added (preface) in this section, an extended explanation of the rationale of this comparison clarifies this issue (lines 345-352).
However, we would like to specify our point-of-view on this issue. The comparison with other software is one of the prerequisite criteria of the journal's author guidelines for "Technical Notes" (https://academic.oup.com/gigascience/pages/technical_note). As mentioned there: "The tool or method needs to have been tested, and properly compared to any existing tools or methods used by the community. It does not necessarily have to outperform existing approaches, but it should show innovation in the approach, implementation, or have added benefits that have been needed in this arena." That is the reason we thought about adding the section "Evaluation on real datasets and against other tools" and run these tests in the first place.
According to the reviewer: "PEMA allows to switch from one (existing) tool to another at each step of the filtering process, therefore differences are the result of the combination of tools decided by the user, not of PEMA itself". PEMA does not, indeed, contain novel algorithm development. However, being able to select among a richer pool of tools so as to perform a high quality metabarcoding analysis is the very essence of PEMA. PEMA users being able to combine third-party tools (out of a rich set) in an easy way constitutes an "added benefit that has been needed in this arena" (based on the GigaScience journal aim mentioned above). It is this easiness along with more of PEMA distinctive features (such as checkpoints and partial workflow re-execution) that justify the flexibility claimed in the manuscript title "PEMA: a flexible Pipeline for Environmental DNA Metabarcoding".
By comparing metabarcoding pipelines we cannot argue in a straightforward way that one is better than the other; it is definitely not the aim of this section. By performing these analyses we intend to present the potentials of each of those pipelines as well as their computational needs in each case. More specifically, you cannot say that PEMA is generally faster than any other pipeline; for example, if the CROP algorithm is selected, then for sure the analysis will take a long time. However, a more general view of such analyses does provide the reader with important information and, in all cases, it highlights the importance of its own role when setting the parameters.

Reviewer's comment #3
Finally, I am still not convinced by the usefulness of Figs. 3, 4 and 5 in the main text. This is a technical note and, without any direct comparison, they do not bring much information. I would suggest to either make a synthetic figure or to move them to supplementary material (but there are already many supplementary files).
The main aim of the figures mentioned is to visualize the findings described in the "Evaluation on real datasets and against other tools". We agree with the reviewer's comment that in a technical note they could be avoided and it is our belief that we should remove Figures 3 and 4 at all. However, it is our belief that Figure 5 (Figure 3 in the revised version of the manuscript) should be kept as an example of the visualization that PEMA supports.

Reviewer's comment #4
In further communication, please add line number in the responses to editor/reviewers to indicate where changes have been made.
We have followed the recommendation of the reviewer.

Reviewer’s comment #5
Line 74-78: please reformulate, the current definition of metabarcoding is quite vague.
The definition of metabarcoding has been rephrased (lines 76-79 of the revised version of the manuscript).

Reviewer’s comment #6
Line 78: it is rather a "potential holistic approach"
The sentence was rephrased (line 81 of the revised version of the manuscript).

Reviewer’s comment #7
Line 82-88: for each marker, please explain what taxonomic group(s) it targets. Also, authors could make explicit that any primer pairs amplifying one of these regions can be used as long as paired-end reads can be merged successfully.
With respect to the taxonomic groups, we have followed the recommendation of the reviewer (see lines 76-79). Regarding the primer pairs, it is our belief that as this is a background paragraph describing the metabarcoding method, it would be better not to include it. That is because metabarcoding studies may occur with single end reads as well.

Reviewer’s comment #8
Line 87-88: there are already some pipelines for this.
An example of a pipeline for the ITS marker gene has been added (line 90 of the revised version of the manuscript). The main point of this sentence is to underlie the need for a fast and flexible pipeline for those two marker genes (COI and ITS) too.

Reviewer’s comment #9
Line 112-115: this paragraph could place later to increase readability (e.g. after the next paragraph or in the discussion)
It is our belief that this paragraph should remain in its current place in the manuscript (lines 117-120 in the revised version of the manuscript). Its aim is to justify what comes to the exact next paragraph; PEMA supports both OTU clustering and ASV inference because of the fact that “The use of ASVs instead of OTUs has been suggested [14], however the choice for which approach to use should rely on each study’s objective(s) [15].” Furthermore, we consider that a definition of the ASVs is needed here.

Reviewer’s comment #10
Line 137-138: What about samples with a low number of reads? This could be part of the initial quality check.
We have followed the recommendation of the reviewer and changed the sentence accordingly (lines 144-145).

Reviewer’s comment #11
Line 164-165: Is there two chimera removal steps: Vsearch in Part 1 and later step in part 3? Or is it only when using Swarm? Can you please explain.
The chimera removal step occurs only once in all cases, as it is also shown in the figure describing the pipeline (Figure 1). What changes is the order of the steps, depending on the algorithms selected by the user. If the Swarm v2 algorithm has been selected, then the chimera removal takes place after the ASV inference. In all other cases, the chimera removal step occurs before the OTU clustering. We added a sentence at the end of the previous section (“Part 1: Quality control and pre-processing of raw data”) to clarify this (lines 153-155).

Reviewer’s comment #12
Lines 238-241: Please reformulate
We have followed the recommendation of the reviewer and rephrased the sentence (lines 247-255).

Reviewer’s comment #13
Line 249 and additional file 2: The description of the tools and parameters used for each dataset (as well as the rationales for choosing them) would be welcomed here.
As mentioned in the manuscript, the tools and the parameters used in each run can be found in the “Additional file 2: Mock communities”. More specifically, a separate sheet for each marker gene can be found in this document, where PEMA's output as long as the corresponding statistics are shown for each tool and parameter set. Regarding the rationales for choosing those mock communities, a paragraph has been added in the “Additional file 2: Mock communities” on the "datasets" sheet.

Reviewer’s comment #14
Line 258-317: This section could be reduced by removing too species-specific details (e.g. lines 283-285).
As we share the common belief for a not excessively long manuscript, we removed any species-specific and mock-community-specific details for the manuscript as possible (e.g. lines 283-285 of the previous version of the manuscript). However, as it is of great importance to distinguish PEMA’s false positives and true negatives from those that occur due to the datasets’ features we kept such details when considered that is needed to that end.

Reviewer’s comment #15
Line 270-273: This paragraph should be moved elsewhere as it is valid for all markers.
We have followed the recommendation of the reviewer. The paragraph has been moved at the end of the previous section (lines 272-276).

Reviewer’s comment #16
Lines 311-314: unclear, please reformulate.
We have followed the recommendation of the reviewer and reformulated the sentence (lines 322-327).

Reviewer’s comment #17
Lines 429-440: References would be welcomed here.
We have followed the recommendation of the reviewer (lines 446-458).

Reviewer’s comment #18
Line 437-440: Please explain more in details what you mean here. I am not sure I fully agree with this statement.
We have followed the recommendation of the reviewer (lines 455-458). The point of this paragraph is to discuss that by making use of ASVs, especially in the case of microbial communities, we might end up with a vast number of sequences, both because of high alpha- (intra-sample variation) and beta (inter-sample variation) diversity. This complicates even more downstream statistical analyses and the drawing of conclusions about the dynamics of the communities under study.

Reviewer’s comment #19
Table 5: This table does not seem necessary now that authors added mock community analyses, especially if the original community is unknown. It seems redundant.
The aim of this study is to show PEMA’s performance from a biological point-of-view in real data, compared to those of Barque. As mentioned in the table’s label, the comparison is against the initial study’s positive controls. Thus, the output of two different pipelines can be compared as the composition of those samples is known. For the case of the Pavloudi et al. dataset, there is no such positive controls and that is why there is not an equivalent table presented.

Reviewer’s comment #20
Table S2 (previously Table 5): I still do not understand what the authors mean by "N = total microbial relative abundance". It seems to me that these numbers represent the number of reads? If so, the term "relative abundance" is inappropriate and confusing (one would expect a percentage).
High throughput sequencing can only provide relative estimates of abundances. Absolute abundances can be provided by other methods, such as quantitative PCR (Q-PCR). Therefore, it is well established that high throughput sequencing can only result in relative abundances of taxa.
“N” has been used extensively among biodiversity scientists to denote the “total number of individuals” of a given taxon, therefore its “total abundance” (for example, see: 10.1016/S0022-0981(98)00028-8). Thus, in our case, as well as in other studies where it has been used to describe metabarcoding data, it denotes the “total microbial relative abundance values” (see: 10.7717/peerj.3687).

For example, consider the example below, of a classic abundance table (taxa per stations):

,Station A,Station B
Taxon 1,15,22
Taxon 2,0,52
Taxon 3,2,34

In this example case, when one would calculate the classic biodiversity indices, the result would be the

following:

,S (number of taxa),N (total abundance)
Station A,2,17
Station B,3,108
SUM,5,125

If these indices were to be presented as percentages, the result would be:

,Percentage of each station’s number of taxa to the sum of the number of taxa found in all the stations of the study,Percentage of each station’s total abundance of taxa to the sum of the total abundances of taxa found in all the stations of the study
Station A,40 %,13.6 %
Station B,60 %,86.4 %

The logic is the same, however we cannot use the term “total abundance”. These numbers do not just represent the number of reads, since the reads have undergone all the necessary processing (which is thoroughly explained in the manuscript) in order to derive to the final (M)OTU/ASV table (the equivalent of the classic abundance table in the case of eDNA metabarcoding). The number of sequences, i.e. reads) after each pre-processing step are shown in Additional file 3: Table S1.

Close
