# Supplementary material for: PEMA: a flexible Pipeline for Environmental DNA Metabarcoding Analysis of the 16S/18S ribosomal RNA, ITS, and COI marker genes
Source: Gigascience. 2020 Mar 12;9(3):giaa022. doi: 10.1093/gigascience/giaa022 (PMC7066391; doi:10.1093/gigascience/giaa022)
Supplement: giaa022_Reviewer_1_Report_Original_Submission — Johan Andre Pansu -- 12/17/2019 Reviewed [file giaa022_reviewer_1_report_original_submission.pdf]

Reviewer Report

Title: PEMA: a flexible Pipeline for Environmental DNA Metabarcoding Analysis of the 16S/18S rRNA, ITS and COI marker genes

Version: Original Submission **Date:** 12/17/2019

Reviewer name: Johan Andre Pansu

Reviewer Comments to Author:

I have reviewed for the second time the manuscript from Zafeiropoulos et al. "PEMA: a flexible Pipeline for Environmental DNA Metabarcoding Analysis of the 16S/18S rRNA, ITS and COI marker genes". First, I would like to acknowledge the major improvements implemented by the authors in their pipeline since the first submission, namely: the extension of the functionality to two other marker genes (18S rRNA and ITS) and the inclusion of tools for inferring ASV. This is undoubtedly a great set of additional tools, and the pipeline now covers four of the most common markers for eDNA studies (but not all marker genes as mentioned in the responses). That said, attending that similar pipelines already exist (see PipeCraft for another example; Anslan et al. 2017 Molecular Ecology), I think the main strength of this pipeline is in flexibility, time efficiency and ability to handle large datasets.
I appreciate the inclusion of mock community analyses that, contrarily to real eDNA dataset, allow the reader to have an idea of what to expect and easily compare outputs of the pipeline with the biological reality (although this section could gain in readability by avoiding study-specific details). However, I am still very puzzled by the comparisons with other software. I agree with authors when they say that "it is important to assess the variability that different tools introduce in the produced outputs of each study", and PEMA allows that by offering different options at every step of the pipeline, but it seems abusive to say that results from PEMA outperforms those of other pipelines (e.g. lines 370-372). Comparing pipelines make sense when there is algorithm development but here PEMA allows to switch from one (existing) tool to another at each step of the filtering process, therefore differences are the result of the combination of tools decided by the user, not of PEMA itself. I appreciate the inclusion of mock community analyses that, contrarily to real eDNA dataset, allow the reader to have an idea of what to expect and easily compare outputs of the pipeline with the biological reality (although this section could gain in readability by avoiding study-specific details). However, I am still very puzzled by the comparisons with other software. I agree with authors when they say that "it is important to assess the variability that different tools introduce in the produced outputs of each study", and PEMA allows that by offering different options at every step of the pipeline, but it seems abusive to say that results from PEMA outperforms those of other pipelines (e.g. lines 370-372). Comparing pipelines make sense when there is algorithm development but here PEMA allows to switch from one (existing) tool to another at each step of the filtering process, therefore differences are the result of the combination of tools decided by the user, not of PEMA itself. In my opinion, these comparisons can appear as misleading, and make the paper more complicated and longer than needed.
Finally, I am still not convinced by the usefulness of Figs. 3, 4 and 5 in the main text. This is a technical note and, without any direct comparison, they do not bring much information. I would suggest to either

make a synthetic figure or to move them to supplementary material (but there are already many supplementary files).
In further communication, please add line number in the responses to editor/reviewers to indicate where changes have been made.
Minor comments:
Line 74-78: please reformulate, the current definition of metabarcoding is quite vague.
Line 78: it is rather a "potential holistic approach"
Line 82-88: for each marker, please explain what taxonomic group(s) it targets. Also, authors could make explicit that any primer pairs amplifying one of these regions can be used as long as paired-end reads can be merged successfully.
Line 87-88: there are already some pipelines for this.
Line 112-115: this paragraph could place later to increase readability (e.g. after the next paragraph or in the discussion)
Line 137-138: What about samples with a low number of reads? This could be part of the initial quality check.
Line 164-165: Is there two chimera removal steps: Vsearch in Part 1 and later step in part 3? Or is it only when using Swarm? Can you please explain.
Lines 238-241: Please reformulate
Line 249 and additional file 2: The description of the tools and parameters used for each dataset (as well as the rationales for choosing them) would be welcomed here.
Line 258-317: This section could be reduced by removing too species-specific details (e.g. lines 283-285).
Line 270-273: This paragraph should be moved elsewhere as it is valid for all markers.
Lines 311-314: unclear, please reformulate.
Lines 429-440: References would be welcomed here.
Line 437-440: Please explain more in details what you mean here. I am not sure I fully agree with this statement.
Table 5: This table does not seem necessary now that authors added mock community analyses, especially if the original community is unknown. It seems redundant.
Table S2 (previously Table 5): I still do not understand what authors mean by "N = total microbial relative abundance". It seems to me that these numbers represent the number of reads? If so, the term "relative abundance" is inappropriate and confusing (one would expect a percentage).

Level of Interest

Please indicate how interesting you found the manuscript: Choose an item.

Quality of Written English

Please indicate the quality of language in the manuscript: Choose an item.

Declaration of Competing Interests

Please complete a declaration of competing interests, considering the following questions:

- Have you in the past five years received reimbursements, fees, funding, or salary from an organisation that may in any way gain or lose financially from the publication of this manuscript, either now or in the future?
- Do you hold any stocks or shares in an organisation that may in any way gain or lose financially from the publication of this manuscript, either now or in the future?
- Do you hold or are you currently applying for any patents relating to the content of the manuscript?
- Have you received reimbursements, fees, funding, or salary from an organization that holds or has applied for patents relating to the content of the manuscript?
- Do you have any other financial competing interests?
- Do you have any non-financial competing interests in relation to this paper?

If you can answer no to all of the above, write 'I declare that I have no competing interests' below. If your reply is yes to any, please give details below.

I declare that I have no competing interests'

I agree to the open peer review policy of the journal. I understand that my name will be included on my report to the authors and, if the manuscript is accepted for publication, my named report including any attachments I upload will be posted on the website along with the authors' responses. I agree for my report to be made available under an Open Access Creative Commons CC-BY license (http://creativecommons.org/licenses/by/4.0/). I understand that any comments which I do not wish to be included in my named report can be included as confidential comments to the editors, which will not be published.

Choose an item.

To further support our reviewers, we have joined with Publons, where you can gain additional credit to further highlight your hard work (see: https://publons.com/journal/530/gigascience). On publication of this paper, your review will be automatically added to Publons, you can then choose whether or not to claim your Publons credit. I understand this statement.

Yes Choose an item.
